# Effectiveness and safety of eleven Chinese patent medicines combined with atorvastatin in the treatment of hyperlipidemia: a network meta-analysis of randomized controlled trials

**DOI:** 10.3389/fendo.2025.1523553

**Published:** 2025-03-24

**Authors:** Zeyang Shi, Wei Zheng, Zhijun Bu, Xian Zhou, Xuefeng Wang, Yike Song, Jialin Sun, Jian-Ping Liu, Zhao-lan Liu

**Affiliations:** ^1^ Centre for Evidence-Based Chinese Medicine, Beijing University of Chinese Medicine, Beijing, China; ^2^ Dongzhimen Hospital, Beijing University of Chinese Medicine, Beijing, China; ^3^ NICM Health Research Institute, Western Sydney University, Sydney, NSW, Australia; ^4^ School of Traditional Chinese Medicine, Beijing University of Chinese Medicine, Beijing, China

**Keywords:** Chinese patent medicine, atorvastatin, hyperlipidemia, network meta-analysis, randomized controlled trial

## Abstract

**Background:**

Randomized controlled trials (RCTs) suggested that combining Chinese patent medicines with atorvastatin exhibited superior effectiveness in treating hyperlipidemia with reduced adverse reactions. However, the evidence regarding the clinical effectiveness and safety was not assessed to make informed decision in clinical practice.

**Objective:**

To evaluate the clinical effectiveness and safety of combined Chinese patent medicines with atorvastatin.

**Methods:**

Eight databases (CNKI, Wanfang, VIP, SinoMed, PubMed, Embase, Web of Science, and Cochrane Library) were searched from inception to April 2024. The risk of bias (ROB) of the included RCTs was assessed using the ROB 2.0 tool recommended by the Cochrane Handbook. The surface under the cumulative ranking curve (SUCRA) probability values were used to rank the treatment measures, and the Confidence in Network Meta-Analysis (CINeMA) software was used to assess the grading of evidence.

**Results:**

A total of 23 RCTs involving 2184 patients with hyperlipidemia were included. Hedan (tablets or capsules) combined with atorvastatin showed the highest clinical effectiveness with an RR of 1.58 (95% CI: [1.14, 2.12]) (SUCRA: 80.36%), the lowest post-treatment low-density lipoprotein cholesterol (LDL-C) level with an MD of -8.13(95% CI: [-9.70, -6.57]) (SUCRA: 3.37%), and the lowest post-treatment triglyceride (TG) level with an MD of -6.43(95% CI: [-7.71, -5.16]) (SUCRA: 0.33%). Dantian Jiangzhi Granules demonstrated the lowest post-treatment total cholesterol (TC) level with an MD of -2.22(95% CI: [-2.60, -1.83]) (SUCRA: 12.6%), while Xuezhikang Capsules displayed the highest post-treatment high-density lipoprotein cholesterol (HDL-C) level with an MD of 1.61(95% CI: [0.82, 2.39]) (SUCRA: 79.87%). However, it is important to note that most of the included studies showed “some concerns” regarding the risk of bias based on ROB 2.0. According to CINeMA, most confidence rating results were classified as “low”.

**Conclusion:**

Compared to atorvastatin alone, the combination of Chinese patent medicines with atorvastatin demonstrated superior effectiveness in treating hyperlipidemia. Among these, Hedan (tablets or capsules) exhibited the greatest overall benefit, significantly reducing TG and LDL-C levels. Dantian Jiangzhi Granules had the most pronounced effect in lowering TC, while Xuezhikang was most effective in improving HDL-C level. Although Xuezhikang is well-documented as lipid-lowering agent, the findings of this study suggest that hyperlipidemia treatment should be tailored to individual blood lipid profiles. Additionally, for drugs with limited evidence of efficacy and safety, larger randomized controlled trials and further pharmacological studies are necessary to valid these results.

**Systematic review registration:**

https://www.crd.york.ac.uk/prospero/, identifier CRD42024573421.

## 1 Introduction

Hyperlipidemia represents a pathological state of metabolic disorder, characterized primarily by elevated levels of serum total cholesterol, triglyceride, and low-density lipoprotein cholesterol, or decreased levels of high-density lipoprotein cholesterol (1). It arises from lipid metabolism defects on the surface of apolipoprotein C-II, lipoprotein lipase activity deficiencies, as well as genetic, dietary, and environmental factors. Hyperlipidemia is a common clinical condition and serves as an independent risk factor for cardiovascular and cerebrovascular diseases such as coronary atherosclerotic heart disease, hypertension, and cerebral infarction (2). Recent reviews (3–5) and the 2019 Global Burden of Disease(GBD) report (https://vizhub.healthdata.org/gbd-results/) indicated that globally, 3.78 million deaths from ischemic heart disease (IHD) can be attributed to high LDL-C level, accounting for 44.3% of all IHD deaths, a 46.2% increase from 1990. Additionally, 0.61 million deaths from ischemic stroke are attributed to high LDL-C, representing 22.4% of all ischemic stroke deaths, with a 47.8% increase since 1990. Furthermore, from 1990 to 2017, deaths due to non-HDL-C more than doubled from 0.25 million to 0.86 million, with a nearly two-fold increase in Southeast Asia from 0.11 million to 0.31 million. Small increases in non-HDL-C-related deaths were also observed in Latin America, Central Asia, and Africa. Moreover, a survey from the Million Person Project for Early Screening and Comprehensive Intervention of High-Risk Cardiovascular Disease in China from 2014-2019 (6) revealed prevalence rates of high TC, high LDL-C, low HDL-C, and high TG to be 6.9%, 8.1%, 20.4%, and 13.8%, respectively. A study on the leading causes of death among US adults (7) demonstrated that patients with hyperlipidemia have approximately twice the risk of cardiovascular disease compared to those with normal total cholesterol levels. Statins slow down endogenous cholesterol synthesis by inhibiting the rate-limiting enzyme of cholesterol synthesis, namely HMG-CoA reductase, and increase the expression of LDL receptor on the surface of cell membrane, reactively increase the uptake of LDL in blood by hepatocytes and accelerate the clearance of LDL in blood. At the same time, it reduces the level of very low density lipoprotein (VLDL) by reducing the synthesis of apolipoprotein B100 (ApoB100) in the liver, and ultimately reduces the levels of VLDL, triglyceride (TG), ApoB100, etc., and increases the level of high density lipoprotein cholesterol (HDL-C) (2). According to the Chinese Guidelines for the Prevention and Treatment of Dyslipidemia in Adults (8) and A Report of the American College of Cardiology/American Heart Association Task Force on Clinical Practice Guidelines (9), we chose atorvastatin as a western medicine treatment. However, it has shown in clinical practice that long-term use of statins can lead to varying degrees of adverse reactions, including elevated liver enzymes, muscle pain, rhabdomyolysis, and hyperglycemia (10).

Traditional Chinese medicine (TCM) plays an important role in the treatment of hyperlipidemia. It can not only reduce the adverse reactions induced by lipid-lowering drugs such as statins, but also assist in lipid-lowering, thereby maintaining blood lipids at a relatively safe and stable range (11). Clinical practices have proved (12, 13) that compared with simple western medicine, the combined treatment of TCM and western medicine can not only strengthen the lipid-lowering effect, but also tailor treatments according to patient’s individual conditions, alleviate adverse reactions caused by western medicine, improve the patient’s medication experience and satisfaction, ultimately achieving safe lipid-lowering. At present, the integration of TCM and western medicine for the prevention and treatment of hyperlipidemia has gained wider acceptance. *Xu (* 14) treated patients with hyperlipidemia of phlegm-stasis type using western medicine combined with a self-prescribed Xiaozhuo Decoction; *Zhang (* 15) employed simvastatin in conjunction with modified Taohong Siwu Decoction and Xiaoyao Powder for hyperlipidemia patients; *Wang (* 16) used Jiangzhi Tongluo Soft Capsule combined with western medicine to treat patients with mixed hyperlipidemia Through clinical trials, it was found that not only did the treatment groups receiving combined Chinese and western medicine exhibit stronger lipid-lowering effects than the control groups receiving western medicine alone, but also alleviated other discomforting symptoms. According to the commonly used Chinese patent medicines recommended in the past (17) and the Chinese patent medicines with lipid-lowering effects in the Chinese medical insurance catalogue, eleven kinds of Chinese patent medicines were selected for research. The latest Chinese medical insurance catalogue is available on (https://www.nhsa.gov.cn/art/2023/12/13/art_104_11673.html/).

In the view of TCM, hyperlipidemia is a pathological state of lipid metabolism disorder caused by multiple factors. The pathogenic factors include improper diet, excessive intake of fatty food and lack of physical activity, deficiency of essence and qi, spleen deficiency and physical decline, emotional disharmony, spleen qi stagnation caused by overthinking, disharmony between liver and spleen. The primary locations of hyperlipidemia in the body according to TCM are the spleen, liver and kidney. The pathogenesis is spleen qi deficiency, dysfunction of spleen in transportation, and disharmony between liver and spleen (18). According to TCM syndrome differentiation and treatment principles, different therapeutic drugs should be selected for different symptoms. From the perspective of phlegm-dampness, prescriptions like Shenling Baizhu Powder and Erchen Decoction can be prescribed (19); In cases involving phlegm-blood stasis, a combination of Banxia Baizhu Tianma Decoction and Taohong Siwu Decoction may be used (20). For those with spleen deficiency, different prescriptions are also available (21). Moreover, for patients with qi deficiency and blood stasis, Buyang Huanwu Decoction can be prescribed, while for those with blood turbidity, a combination of Huazhuo Xingxue Decoction and Hedan Tablets may be suitable (21).

Network meta-analysis is a new statistical analysis method of evidence-based medicine that combines direct and indirect evidence to evaluate a variety of treatment methods in a single analysis and expands the principle of traditional meta-analysis (22, 23). NMA is capable of simultaneously assessing various interventions, even when direct comparisons are not feasible, thereby providing valuable information for clinical decision-making. In addition, NMA allows to rank each intervention based on its effectiveness and probability of becoming the best treatment (24). Previous systematic reviews have proved the significant clinical effectiveness of atorvastatin combined Chinese patent medicines in treating hyperlipidemia, with minimal adverse reactions. However, there are some problems such as lack of comprehensiveness, rigor, and objectivity, as well as the safety of Chinese patent medicines remains to be further assessed. For this reason, this study rigorously screened and organized the published relevant clinical research literature and used NMA method to comprehensively and systematically evaluate the clinical effectiveness and safety of atorvastatin combined with Chinese patent medicine in treating hyperlipidemia, aiming to provide reliable evidence-based support for clinical practice, guide clinical decision-making and offer reference for clinicians in medication decisions.

## 2 Methods

The 2020 Preferred Reporting Items for Systematic Review (PRISMA) guidelines (25) and the Cochrane Intervention System Review Manual (26)were used to report this systematic review. In **Supplementary Material 1**, a list of PRISMA was provided. We also have registered an agreement in PROSPERO with the registration number CRD42024573421.

### 2.1 Search strategy

We applied the search strategy to eight databases: China Knowledge Infrastructure (CNKI), Wanfang Data, China Science Journal Database(VIP), SinoMed, PubMed, Cochrane Library, Excerpt Medica Database (Embase) and Web of Science (WOS). Medical subject headings (MeSH) and free text words were merged. **Supplementary Material 2** lists the search strategy of the corresponding database. Only Chinese and English studies are included.

### 2.2 Eligibility criteria

1. Study type: Published RCTs

2. Participants: According to the *Guidelines for Prevention and Treatment of Dyslipidemia in Chinese Adults* (8) and the *Guiding Principles for Clinical Research of New Traditional Chinese Medicines (* 27), patients with hyperlipidemia were clinically diagnosed, regardless of age, sex or race.

3. Intervention group: Patients with hyperlipidemia treated with atorvastatin combined with Chinese patent medicine.

4. Outcome: The main outcomes were the levels of triglyceride, cholesterol, HDL-C and LDL-C after treatment. The secondary outcomes included clinical effectiveness and adverse reactions. Among them, the clinical effectiveness is based on the *Guidelines for Prevention and Treatment of Dyslipidemia in Chinese Adults* (8) or the *Guiding Principles for Clinical Research of New Traditional Chinese Medicines* (27), both of which have different classification criteria for clinical effectiveness. *The Guiding Principles for Clinical Research of New Traditional Chinese Medicines* adopt a four-level effectiveness evaluation standard of marked improvement, improvement, ineffectiveness, and deterioration. Marked improvement is defined as a≥20% decrease in TC, or a≥40% decrease in TG, or an increase in HDL-C≥0.26 mmol/L. Improvement is defined as a 10% to 20% decrease in TC, or a 20% to 40% decrease in TG, or an increase in HDL-C>0.10 to 0.26 mmol/L. Ineffectiveness is defined as blood lipid levels not meeting the above criteria. Deterioration is defined as a≥10% increase in TC, or a≥10% increase in TG, or a≥0.18 mmol/L decrease in HDL-C. According to the *Guiding Principles for Clinical Research of New Traditional Chinese Medicines*, the clinical effectiveness is judged as follows: (1) Clinical control: all blood lipid indicators return to normal levels. (2) Marked improvement: ≥1 of the following conditions is met: TC decreases by≥20%, TG decreases by≥40%, LDL-C decreases by≥20%, or HDL-C increases by≥0.26 mmol/L. (3) Improvement: ≥1 of the following conditions is met: TC decreases by≥10% but<20%, TG decreases by≥20% but<40%, LDL-C decreases by≥10% but<20%, or HDL-C increases by≥0.10 mmol/L but<0.26 mmol/L. (4) Ineffectiveness: blood lipid levels do not decrease to the above standards. The clinical effectiveness rates for the two guidelines are calculated as ((marked improvement + improvement)/total number of cases) and ((clinical control + marked improvement + improvement)/total number of cases), respectively.

### 2.3 Study selection

Two reviewers independently read the title, abstract, and full text to identify appropriate studies and extract data from eligible studies. A third reviewer examined the database if there was any discrepancy in the data extraction, the third reviewer would assist to reach a consensus. The data we extracted were as follows: the first author’s name, publication year, sample size (number of patients with hyperlipidemia in the intervention group and control group), mean age (mean age of the intervention group and control group), treatment method (the treatment mode of the intervention group and control group), drug dose, drug usage frequency of the intervention group and control group, treatment duration, and outcome measurements.

### 2.4 Risk of bias and evidence quality assessment

Two reviewers evaluated the bias of the included studies using the Cochrane bias risk tool. After the evaluation, if there was any disagreement, the third reviewer was consulted, and the three reviewers jointly determines the final bias evaluation results. We evaluated the quality of the included studies as follows (28): (a) bias in the randomization process, (b) bias in the expected intervention, (c) bias in missing outcome data, (d) bias in selective outcome reporting and (e) bias of selective outcome reporting. Each aspect was evaluated according to the level of (a) low risk; (b) unknown risk; (c) high risk.

### 2.5 Statistical analysis

Bayesian NMA’s R 4.3.1 gemtc package was used for analysis. We used relative risk (RR) and 95% CI as a measure of binary outcome, and mean difference (MD) and 95% CI as a measure of continuous variables. We set the number of pre-iterations and iterations to 2000 and 50000, respectively. Based on the trajectory map, density map and Brooks-Gelman-Rubin diagnostic map, we determined whether a satisfactory degree of convergence had been achieved. If randomized controlled trials showed good homogeneity in article design, intervention details, control details, and outcomes, a random-effects model was used for analysis. If there was heterogeneity between the results(I^2^>50% or P<0.1), further subgroup analysis was performed. In addition, based on R 4.3.1 software and Stata/SE 18.0 software, data processing, network evidence map, heterogeneity analysis and forest map were completed in turn. We calculated the lower surface of the cumulative ranking curve (SUCRA) values for each outcome measurement and different interventions and used a line chart to reflect the level of blood fats after different treatments. STATA 18.0 was used to detect publication bias. We used funnel plots to assess potential publication bias and used the Egger’s test for validation. In all included studies, there was no closed loop between intervention and control measures.

## 3 Results

### 3.1 Research selection

**Figure 1:**
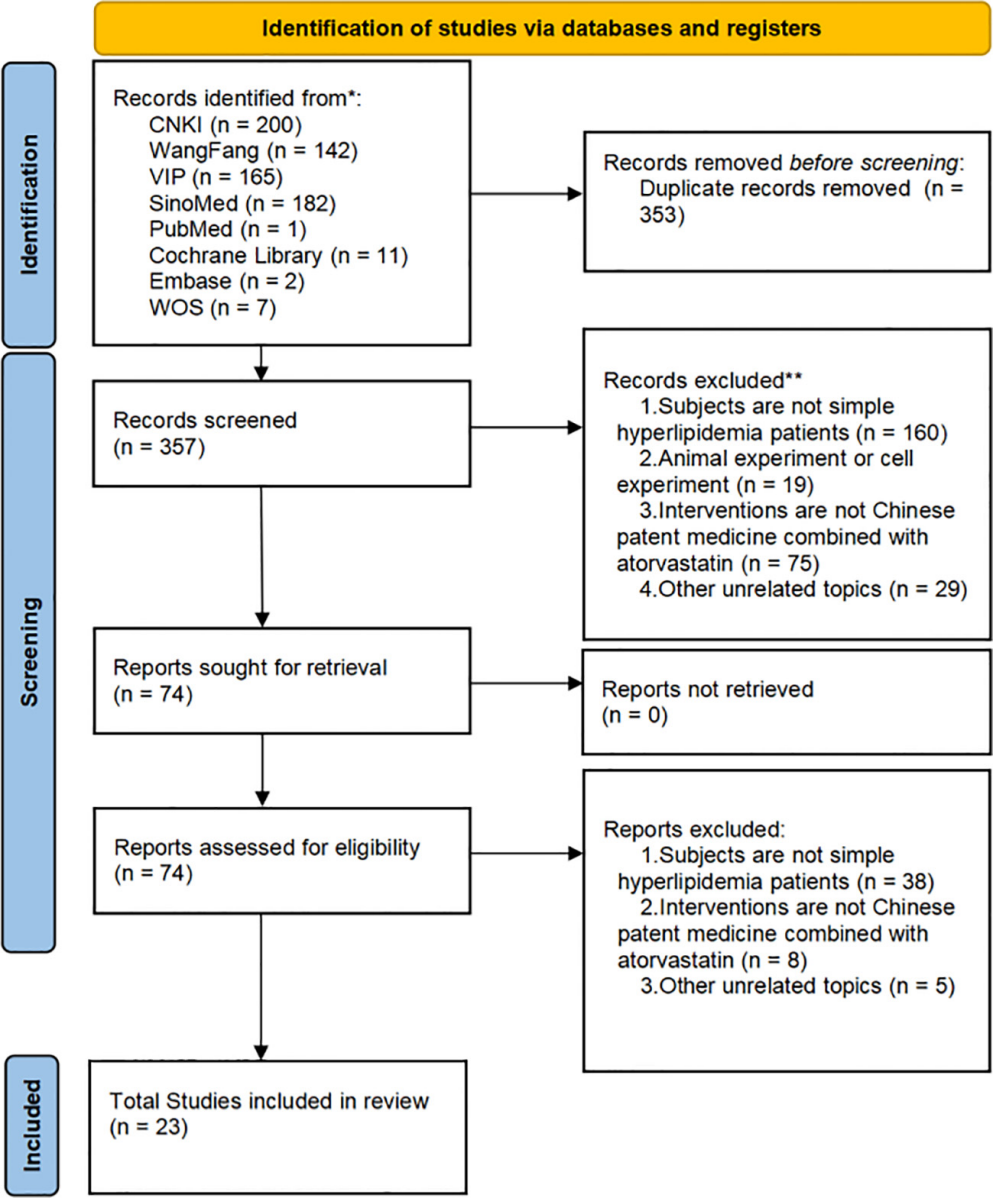
Flowchart of the search for eligible studies. CNKI, China National Knowledge Infrastructure; SinoMed, the Chinese Biomedical Literature Database; WanFang, the WanFang Database; VIP, the Chinese Science Journals Full-Text Database; Embase Database, Excerpta Medica Database; WOS Database, Web of Science Database.

We finally identified 710 studies from eight databases. Among them, 335 duplicates were found using Noteexpress 4.0.0.9788, and an additional 18 studies were excluded. The full text of 357 reservation studies was screened, and 23 studies were identified for inclusion in our review. The flow diagram is shown in **Figure 1**.

### 3.2 Study characteristics

The 23 included studies reported 2184 patients with hyperlipidemia, and the pathological diagnosis of these patients was hyperlipidemia. Among them, 1093 cases in the intervention group and 1091 cases in the control group. A total of 11 different Chinese patent medicine were used in these 23 studies, including Zhibitai Capsules, Jiangzhi Tongluo Soft Capsules, Jiangzhi Tongmai Capsules, Pushen Capsules, Xuezhikang Capsules, Yindan Xinnaotong Soft Capsules, Ginkgo Leaf Tablets, Songling Xuemaikang Capsules, Hedan Tablets, Danxiang Qingzhi Granules, Dantian Jiangzhi Pills. All Chinese patent medicine and their composition information are listed on the website of the *China National Medical Products Administration* (https://www.nmpa.gov.cn) and the *2020 Edition of the Pharmacopoeia of the People’s Republic of China* (https://ydz.chp.org.cn/# main). In addition, classificational verification of the components of all Chinese patent medicine has been conducted on the following three websites (http://mpns.kew.org/mpns-portal/, http://www.plantsoftheworldonline.org, https://www.catalogueoflife.org/). In **Supplementary Material 3**, we have described the detailed information of the components, indications, adverse reactions, contraindications, and precautions of these Chinese patent medicines. Among the 23 included studies, 14 evaluated clinical effectiveness. Specifically, 22 studies assessed TC levels before and after treatment, 23 studies assessed TG levels before and after treatment, 20 studies assessed LDL-C levels before and after treatment, and 21 studies assessed HDL-C levels before and after treatment. In addition, in terms of adverse events, due to significant differences in reporting between studies and the failure of a few studies to separately report adverse reactions in the treatment and control groups, the adverse reactions of each study were reported separately, as shown in **Table 1**. The analysis of the 23 studies identified a prevalent atorvastatin treatment regimen, which specifies that patients should take 10mg or 20mg of atorvastatin orally daily, with the dosage adjusted based on actual blood lipid levels. **Table 2** displays the basic characteristics of the included studies.

**Table 1:** Adverse drug reactions.

Study ID	Adverse reaction (Treatment group)	Adverse reaction (Control group)
Li J 2022 (29)	Gastrointestinal reaction:1 case	Muscle pain:2 cases
Yuan Y 2023 (30)	Nausea and vomiting:1 case Diarrhea:1 case	Rash:1 case Nausea and vomiting:1 case Elevated transaminase:1 case
Chen YY 2020 (31)	Nausea:1 case Loss of appetite:1 case	Nausea:1 case Abdominal distension:1 case Loss of appetite:1 case
Bao FY 2009 (32)	Adverse reactions: 6 cases	Adverse reactions:8 cases
Xie Y 2014 (33)	Mild elevation of ALT:3 cases Mild elevation of AST:1 case Mild elevation of CK:1 case Abdominal discomfort:2 cases: (1 case withdrew from the study)	Mild elevation of ALT:5 cases Mild elevation of AST:4 cases Mild elevation of CK:1 case Abdominal discomfort:2 cases: (1 case withdrew from the study) Upper limb swelling:1 case: (1 case withdrew from the study)
Yin GY 2017 (34)	Abdominal discomfort and upper limb swelling: 2 cases Mild elevation of AST:1 case	
Liu FG 2011 (35)	No adverse reaction	No adverse reaction
You XM 2015 (36)	No adverse reaction	No adverse reaction
Chen X 2016 (37)	Mild elevation of AST and ALT:2 cases	Mild elevation of AST and ALT:2 cases
Cao CY 2014 (38)	Mild twitch of gastrocnemius muscle:1 case	Lower limb weakness:1 case
Yu Q 2021 (41)	Joint swelling:1 case	Skeletal muscle pain:2 cases Joint swelling:3 cases Muscle fatigue:1 case
Hu GY 2015 (42)	Nausea,Abdominal distension and other gastrointestinal symptoms:6 cases	Abdominal distension, fatigue, diarrhea and other symptoms:7 cases
Sun B 2014 (44)	Abdominal distension:1 case Constipation:1 case	Abdominal pain:1 case Dyspepsia:2 cases Abdominal distension:2 cases Constipation:1 case
Wang YF 2015 (45)	Nausea:4 cases Abdominal distension:2 cases Diarrhea:2 cases Headache:1 case	Nausea:11 cases Abdominal distension:4 cases Diarrhea:5 cases Headache:3 cases
Lin FF 2011 (46)	No adverse reaction	No adverse reaction
Yang FM 2017 (48)	No adverse reaction	Upper abdominal discomfort:1 case Nausea:1 case Headache:1 case Abnormal liver function:2 cases (alanine aminotransferase < 80 U/L)
Wu XJ 2009 (49)	No adverse reaction	No adverse reaction

**Table 2:** Characteristics of included studies.

Study ID	Gender (male/ female)	Sample size (I/C)	Mean/Median Age(I/C)	Treatment (Intervention vs Control)	Intervention Details	Control Details	Outcome Details
Li J 2022 (29)	74/46	120 (60/60)	I:79.1 ± 3.8 C:78.2 ± 3.6	M versus.T	M,0.24 g,bid, Course =12 weeks T,10 mg,qd, Course =12 weeks	T,20 mg, qd, Course =12 weeks	2, 3, 4, 5, 6
Yuan Y 2023 (30)	27/31	58 (29/29)	I:52.46 ± 3.89 C:52.24 ± 3.75	M versus.T	M,0.24 g, bid, Course =2 months T,20 mg, qd, Course =2 months	T,20 mg, qd, Course =2 months	1, 2, 3, 4, 5, 6
Chen YY 2020 (31)	64/62	126 (63/63)	I:70.1 ± 7.6 C:71.2 ± 8.1	M versus.T	M,0.24 g,bid, Course =12 weeks T,10 mg, qd, Course =12 weeks	T,10 mg, qd, Course =12 weeks	1, 2, 3, 4, 5, 6
Bao FY 2009 (32)	63/49	112(56/56)	51.1 ± 7.3	D versus.T	D,100 mg, tid, Course =16 weeks T,20 mg,qd, Course =16 weeks	T,20 mg, qd, Course =16 weeks	1, 2, 3, 4
Xie Y 2014 (33)	73/65	138(69/69)	I:55.16 ± 9.77 C:53.72 ± 10.62	D versus.E	D,100 mg,tid, Course =8 weeks T,20 mg,qd, Course =8 weeks	E,100 mg,tid, Course =8 weeks T,20 mg, qd, Course =8 weeks	2, 3, 4, 5, 6,
Yin GY 2017 (34)	70/58	128(64/64)	I:54.49 ± 9.32 C:55.08 ± 9.27	D versus.T	D,100 mg, tid, Course =8 weeks T,20 mg, qd, Course =8 weeks	T,20 mg, qd, Course =8 weeks	2, 3, 4, 5, 6
Liu FG 2011 (35)	34/26	60(30/30)	52.0 ± 9.6	F versus.T	F,1 g,tid, Course =30 days T,20 mg, qd, Course =30 days	T,20 mg, qd, Course =30 days	2, 3, 4, 5, 6
You XM 2015 (36)	35/25	60(30/30)	I:52.7 ± 1.5 C:53.2 ± 1.8	F versus.T	F, Course =8 weeks T,20 mg, qd, Course =8 weeks	T,20 mg, qd, Course =8 weeks	1, 2, 3, 4, 5, 6
Chen X 2016 (37)	26/14	40(20/20)	61.54 ± 10.65	G versus.T	G,0.25 g*4, tid, Course =8 weeks T,20 mg,qd, Course =8 weeks	T,20 mg, qd, Course =8 weeks	2, 3, 4, 5, 6
Cao CY 2014 (38)	57/27	84(42/42)	I:58 C:59	G versus.T	G,0.25 g*4,tid, Course =8 weeks T,20 mg, qd, Course =8 weeks	T,20 mg, qd, Course =8 weeks	1, 2, 3, 4, 5, 6
Wei HW 2015 (39)	80/80	160(80/80)	63.24 ± 11.73	J versus.T	J, 300 mg, qd, Course =30 days T,10 mg, qd, Course =30 days	T,10 mg,qd, Course =30 days	1, 2
Wang ZZ 2013 (40)	30/24	54(27/27)	44.2 ± 4.9	J versus.T	J,0.3 g*2, bid, Course =12 weeks T,20 mg, qd, Course =12 weeks	T,20 mg, qd, Course =12 weeks	1, 2, 3, 4, 5
Yu Q 2021 (41)	38/38	76(38/38)	I:58.69 ± 6.44 C:58.46 ± 6.28	J versus.T	J,0.6 g,bid, Course =3 months T,10 mg, qd, Course =3 months	T,10 mg, qd, Course =3 months	2, 3, 4, 5, 6
Hu GY 2015 (42)	63/57	120(60/60)	I:53.1 C:57.5	J versus.T	J,0.6 g, bid, Course =8 weeks T,10 mg, qd, Course =8 weeks	T,10 mg, qd, Course =8 weeks	1, 2, 3, 4, 6
Chen B 2016 (43)	54/30	84(42/42)	I:62.5 ± 5.6 C:63.5 ± 6.2	M versus.T	M,0.24 g, bid, Course =8 weeks T,10 mg, qd, Course =8 weeks	T,10 mg, qd, Course =8 weeks	1, 2, 3, 4, 5
Sun B 2014 (44)	37/29	66(33/33)	I:72.11 ± 0.23 C:71.65 ± 0.58	M versus.T	M,0.24 g, bid, Course =12 weeks T,10 mg, qd, Course =12weeks	T,10 mg, qd, Course =12weeks	1, 2, 3, 4, 5, 6
Wang YF 2015 (45)	109/78	187(94/93)	I:51.2 ± 4.7 C:50.7 ± 5.1	K versus.T	K,0.4 g*3 ~0.4 g*4, tid, Course =30 days T,20 mg,qd, Course =30 days	T,20 mg,qd, Course =30 days	1, 2, 3, 4, 5, 6
Lin FF 2011 (46)	35/29	64(32/32)	I:51.4 ± 10.8 C:50.1 ± 12.2	L versus.T	L,80 mg,tid, Course =6 weeks T,10 mg,qd, Course =6 weeks	T,10 mg,qd, Course =6 weeks	2, 3, 4, 5
Chen XJ 2016 (47)	53/47	100(50/50)	I:45.6 ± 1.9 C:46.2 ± 2.5	H versus.T	H,tid, Course =6 months T,40 mg,qd, Course =6 months	T,40 mg,qd, Course =6 months	1, 2, 3, 4, 5
Yang FM 2017 (48)	38/22	60(30/30)	NM	C versus.T	C,0.73 g*2,tid, Course =8 weeks T,20 mg,qd, Course =8 weeks	T,20 mg,qd, Course =8 weeks	1, 2, 3, 4, 5, 6
Wu XJ 2009 (49)	36/24	60(30/30)	50 ± 10.5	B versus.T	B,10 g,bid, Course =30 days T,20 mg,qd, Course =30 days	T,20 mg,qd, Course =30 days	2, 3, 4, 5
Zhong YY 2011 (50)	29/30	59(30/29)	I:52.3 ± 11.9 C:51.4 ± 10.1	L versus.T	L,80 mg,tid, Course =6 weeks T,10 mg,qd, Course =6 weeks	T,10 mg,qd, Course =6 weeks	2, 3, 4, 5
Chen JR 2019 (51)	88/80	168(84/84)	I:57.65 ± 6.31 C:58.11 ± 5.29	A versus.T	A,40 mg*50, bid, Course =4 weeks T,20 mg,bid, Course =6 weeks	T,20 mg,bid, Course =6 weeks	1, 2, 3, 5

I, intervention group, C, control group, NM, not mentioned, qd, one time a day, bid, two times a day, tid, three times a day. The specific meaning of treatment column, A, dantian jiangzhi pill, B, danxiang qingzhi granules, C, hedan tablet, D, jiangzhi tongluo soft capsule, E, Jiangzhitongluo soft capsule simulant, F, Jiangzhi Tongmai Capsule, G, pushen capsule, H, songling xuemaikang capsule, J, xuezhikang capsule, K, yindan xinnaotong soft capsule, L, ginkgo leaf tablet, M, Zhibitai Capsule, T, atorvastatin. The meaning of the number represented in the Outcome details, ①, Clinical effectiveness. ②, Triglyceride (TG). ③, Total Cholesterol (TC). ④, high density lipoprotein-cholesterol (HDL-C). ⑤, low-density lipoprotein cholesterol (LDL-C). ⑥, Adverse Reaction.

### 3.3 Risk of bias of included studies

**Figure 2:**
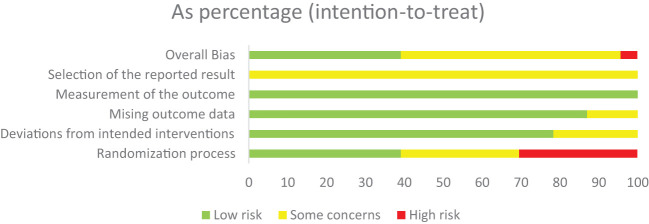
Overall summary risk of bias.

In terms of bias in the randomization process, we found that 9 studies accurately described the generation of random allocation sequences, of which 8 studies used a random number table method and 1 study used a lottery method. Fourteen studies only mentioned randomization, without further explanation of the method. There was no baseline difference between all 23 studies. Only 1 of the 23 studies explicitly stated the use of blinding. The allocation concealment methods of all studies are unclear. In terms of the bias of outcome measurements, only one study had incomplete outcome measurements, mainly due to the dropout of patients in the study. No studies had selectively reporting of outcomes and other risk of bias. All in all, the quality of all included studies was low. The risk of bias and overall risk of bias for each study are shown in **Figure 2**.

### 3.4 Pairwise meta-analysis

We performed a pairwise meta-analysis of all interventions with a total of 5 outcomes. Forest plot and heterogeneity analysis for paired meta-analysis of results are described in **Supplementary Material 4**. In terms of clinical effectiveness, we found that compared with atorvastatin alone, the RR of adding Chinese patent medicine was 1.18(95%CI: [1.13,1.23], Z=7.517, P=0.000), while the TG level after treatment was MD=-1.35(95%CI: [-1.70,-1.00], Z=-7.572, P=0.000), TC level MD=-1.04(95%CI: [-1.44, -0.64], Z=-5.110, P=0.000). HDL-C level MD=0.78(95%CI: [0.44,1.11], Z=4.521, P=0.000), LDL-C level MD=-1.11(95%CI: [-1.55, -0.67], Z=-4.914, P=0.000). All pairwise comparisons between Chinese patent medicine combined with atorvastatin and atorvastatin alone were statistically significant. The results of heterogeneity analysis showed that most combinations of Chinese patent medicine and atorvastatin regimen had high heterogeneity except for clinical effective rate (I^2^ = 0%, P=0.453). Considering the high heterogeneity that may be caused by different interventions, we conducted a subgroup analysis of different interventions. The specific results are shown in **Supplementary Material 4**. After subgroup analysis, we found that there was still significant heterogeneity between pushen capsule, xuezhikang capsule, ginkgo leaf tablet, zhibitai Capsule combined with atorvastatin and atorvastatin alone under TC outcome measurement, which were 90.4%, 94.1%, 87.5% and 94.0%, respectively. Under the LDL-C outcome measurement, there was significant heterogeneity between xuezhikang capsule, ginkgo leaf tablet, zhibitai Capsule combined with atorvastatin and atorvastatin alone, which were 85.2%, 69.5% and 94.4%, respectively. Under the HDL-C outcome index, there was significant heterogeneity between pushen capsule, xuezhikang capsule, ginkgo leaf tablet, zhibitai Capsule combined with atorvastatin and atorvastatin alone, which were 96.9%, 85.4%, 96.4%, and 77.8%, respectively. Under the TG outcome index, there was significant heterogeneity between pushen capsule, xuezhikang capsule, zhibitai Capsule combined with atorvastatin and atorvastatin alone, which were 94.2%, 81.0%, and 94.2%, respectively. A comparative analysis of the thirteen studies involved showed that in the two studies involving pushen capsule combined with atorvastatin intervention, patients in the study of Chen X et al. continued to take the original drugs, while Cao CY et al.’s study did not use other medications, which may lead to an overestimation of the effectiveness of pushen capsule combined with atorvastatin treatment. Among the 4 studies involving xuezhikang capsule combined with atorvastatin intervention, the treatment durations, interventions, and dosages and frequencies of the control measures used varied between studies, which may be the source of heterogeneity. In the two studies involving ginkgo leaf tablet combined with atorvastatin intervention, there may be some methodological heterogeneity due to the lack of blinding. Finally, among the 5 studies involving zhibitai Capsule combined with atorvastatin intervention, there were certain differences in the intervention duration and dosages of traditional Chinese and western medicines used between studies, which may be the source of heterogeneity. Especially in the study of Sun B et al., the maximum dose of atorvastatin in the control group was 80 mg according to the actual situation, which may lead to underestimation of the effectiveness of the intervention group. Due to the large differences between the studies, they cannot be divided into subgroups. However, the combined effect size is more in line with the effectiveness of the real world. Because TCM pays attention to individualized diagnosis and treatment, there are often differences in course of treatment and drug dose for the severity of the disease combined with the individual condition of the patient.

Finally, we conducted a sensitivity analysis of all studies and found that the results were robust and reliable. The results of the specific sensitivity analysis can be seen in **Supplementary Material 5**.

### 3.5 Network meta-analysis

**Figure 3:**
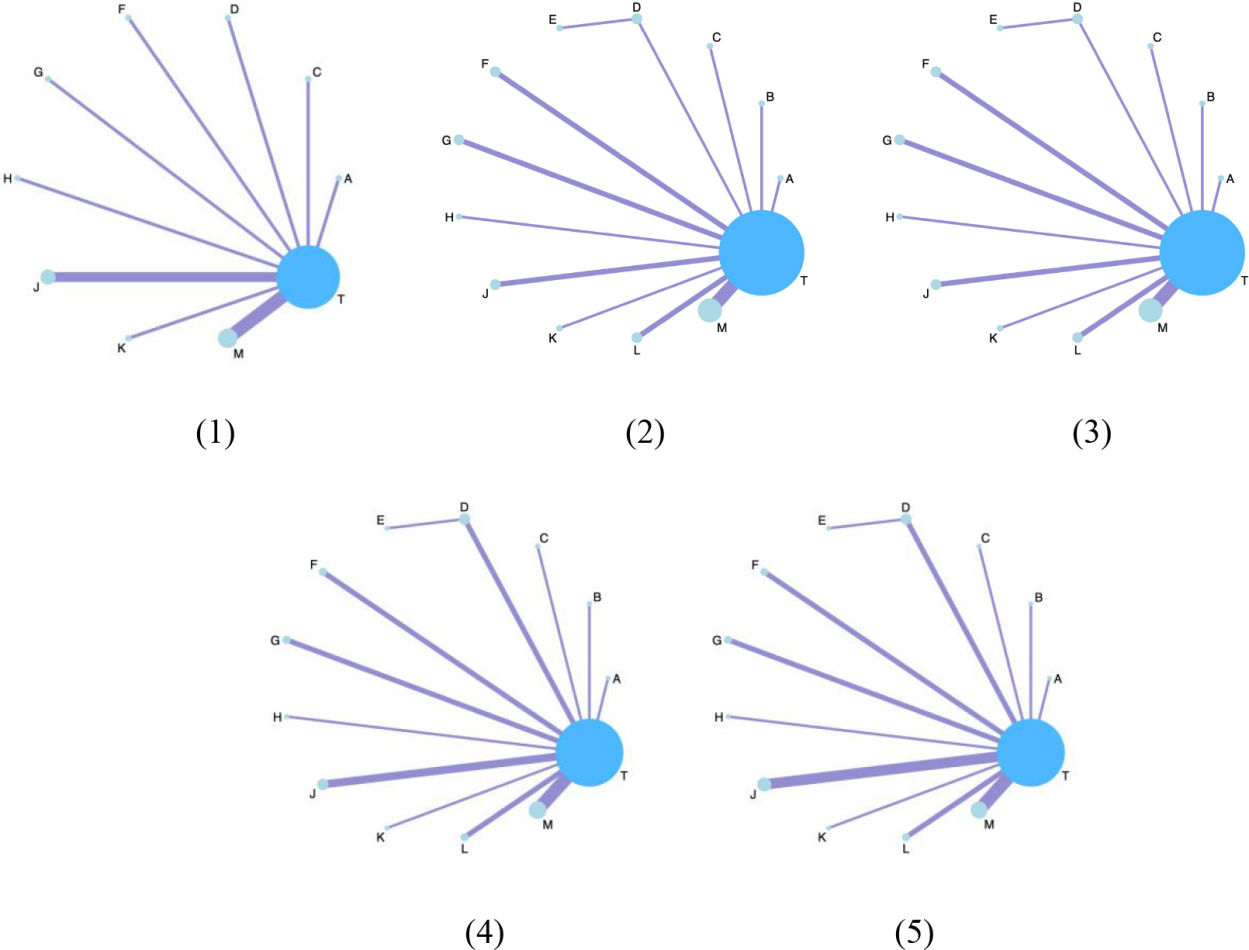
The network structure diagram for outcome measures. Each circle on the diagram represents a treatment, and its size reflects the number of studies evaluating that treatment. The lines connecting the circles indicate direct comparisons between treatments. The figure features five subfigures, each representing a different outcome: (1) Clinical effectiveness; (2) HDL-C; (3) LDL-C; (4) TC; (5) TG. Additionally, each specific Chinese herbal injection is identified as follows: A, dantian jiangzhi pill, B, danxiang qingzhi granules, C, hedan tablet, D, jiangzhi tongluo soft capsule, F, Jiangzhi Tongmai Capsule, G, pushen capsule, H, songling xuemaikang capsule, J, xuezhikang capsule, K, yindan xinnaotong soft capsule, L, ginkgo leaf tablet, M, Zhibitai Capsule, T, atorvastatin.

**Figure 4:**
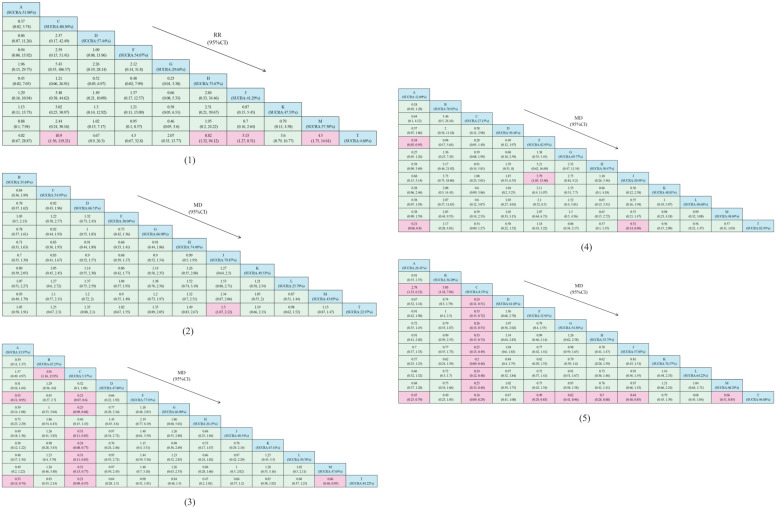
Relative effect analysis of outcomes. Cells filled with blue in the table represent intervention measures, while cells filled with pink indicate statistical significance. (1) Clinical effectiveness; (2) HDL-C; (3) LDL-C; (4) TC; (5) TG.A, dantian jiangzhi pill, B, danxiang qingzhi granules, C, hedan tablet, D, jiangzhi tongluo soft capsule, F, Jiangzhi Tongmai Capsule, G, pushen capsule, H, songling xuemaikang capsule, J, xuezhikang capsule, K, yindan xinnaotong soft capsule, L, ginkgo leaf tablet, M, Zhibitai Capsule, T, atorvastatin.

**Figure 5:**
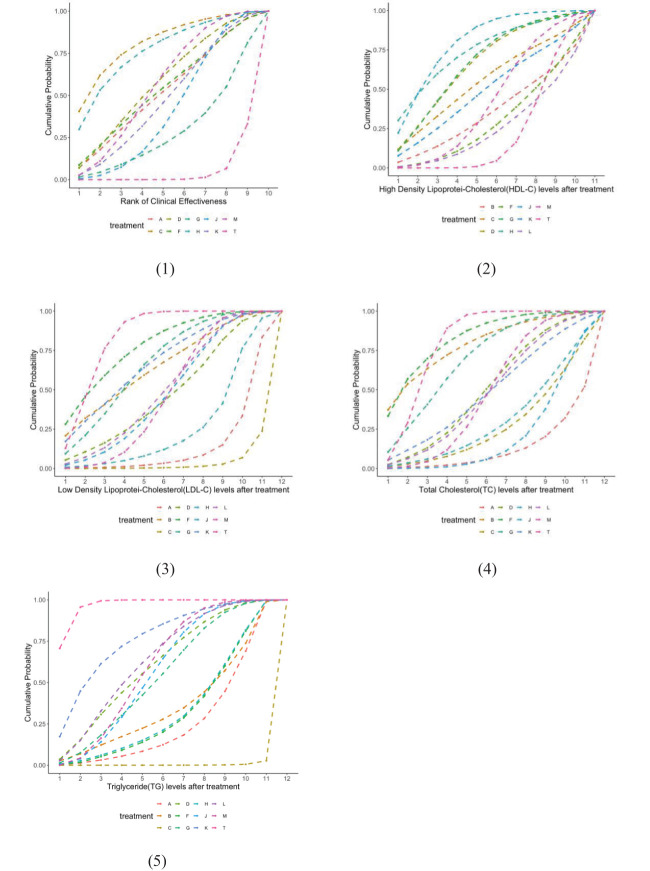
Cumulative probability of ranking blood fat treatment measures. The higher the ranking, the higher the blood fat level.(1) Clinical effectiveness; (2) HDL-C; (3) LDL-C; (4) TC; (5) TG. A, dantian jiangzhi pill, B, danxiang qingzhi granules, C, hedan tablet, D, jiangzhi tongluo soft capsule, F, Jiangzhi Tongmai Capsule, G, pushen capsule, H, songling xuemaikang capsule, J, xuezhikang capsule, K, yindan xinnaotong soft capsule, L, ginkgo leaf tablet, M, Zhibitai Capsule, T, atorvastatin.

The network structure of all results is shown in **Figure 3**. We tested the model convergence of all outcomes. It can be seen from the trajectory diagram and density diagram in **Supplementary Material 6** that all chains overlap with each other and cannot intuitively identify the iterative process of each chain. All curves are close to normal distribution, and the bandwidth value is stable. From the Brooks-Gelman-Rubin diagnostic diagram in **Supplementary Material 7**, it can be seen that the median and 97.5% values of the reduction factor tend to be 1.PSRF values are all 1.Therefore, all the result models have good convergence. The relative effect analysis of the results is shown in **Figure 4**. We calculated the SUCRA values of the five outcomes of different interventions, and used the area under the cumulative curve to reflect the ranking of different treatments, as shown in **Figure 5**.

#### 3.5.1 Main outcome

##### 3.5.1.1 TG

A total of 23 studies reported TG(blood lipid) levels, including 1 involving dantian Jiangzhi Granules,1 involving danxiang qingzhi granules, 1 involving hedan tablet, 3 involving jiangzhi tongluo soft capsule, 2 involving jiangzhi Tongmai Capsule, 2 involving pushen capsule, 1 involving songling xuemaikang capsule, 4 involving xuezhikang capsule, 1 involving yindan xinnaotong soft capsule, 2 involving ginkgo leaf tablet, and 5 involving zhibitai Capsule. Except for one study involving Jiangzhitongluo soft capsule simulant combined with atorvastatin as a control, we combined the effect sizes of a total of 22 studies and separately reported the results of the one uncombined study. Please refer to **Supplementary Document 8**. Compared with atorvastatin alone, A’s MD= -2.24(95%CI: [-2.62, -1.85]), B’s MD= -0.70(95%CI: [-1.22, -0.18]), C’s MD= -6.43(95%CI: [-7.71, -5.16]), D’s MD= -0.38(95%CI: [-0.67, -0.09]), F’s MD= -0.71(95%CI: [-1.08, -0.34]). G’s MD= -1.36(95%CI: [-3.28, 0.57]), H’s MD= -2.33(95%CI: [-2.84, -1.82]), J’s MD= -1.15(95%CI: [-1.66, -0.65]), K’s MD= -1.19(95%CI: [-1.50, -0.88]), L’s MD= -0.96(95%CI: [-1.34, -0.59]), M’s MD= -1.44 (95%CI: [-2.32, -0.55]). Except for G, other research results showed statistical significance. Among them, the use of C may have the greatest impact on reducing TG indicators, and the area under the triglyceride level curve after treatment(SUCRA: 0.326%), while the effect of atorvastatin alone is minimal(SUCRA: 96.88%). From the numerical results of SUCRA, the intervention order of TG index improvement from high to low is: C(SUCRA: 0.33%) > A(SUCRA: 26.41%) > F(SUCRA: 32.91%) > H(SUCRA: 33.75%) > B(SUCRA: 36.20%) > G(SUCRA: 54.36%) > J(SUCRA: 57.03%) > M(SUCRA: 60.29%) > D(SUCRA: 61.03%) > L(SUCRA: 64.22%) > K(SUCRA: 76.57%) > T(SUCRA: 96.88%)

##### 3.5.1.2 TC

A total of 22 studies reported TC(blood lipid) levels, including one involving dantian Jiangzhi Granules, one involving danxiang qingzhi granules, one involving hedan tablet, three involving jiangzhi tongluo soft capsule, two involving jiangzhi Tongmai Capsule, two involving pushen capsule, one involving songling xuemaikang capsule, three involving xuezhikang capsule, one involving yindan xinnaotong soft capsule, two involving ginkgo leaf tablet, and five involving zhibitai Capsule. We combined the effect sizes of 21 studies. Compared with atorvastatin alone, A’s MD= -2.22 (95%CI: [-2.60, -1.83]), B’s MD= 0.13 (95%CI: [-0.38, 0.63]), C’s MD= -2.05(95%CI: [-2.68, -1.42]), D’s MD= -0.48(95%CI: [-0.74, -0.22]), F’s MD= 0.14(95%CI: [-0.21, 0.50]). G’s MD= -0.85(95%CI: [-2.19, 0.49]), H’s MD= -3.92(95%CI: [-4.60, -3.25]), J’s MD= -1.47(95%CI: [-2.70, -0.25]), K’s MD= -0.90(95% CI: [-1.20, -0.60]), L’s MD= -0.64(95%CI: [-1.69, 0.40]), M’s MD= -1.02(95%CI: [-1.85, -0.19]). Except for B, F, G, L, other research results showed statistical significance. Among them, the use of A may have the greatest impact on reducing TC indicators, and the area under the cholesterol level curve after treatment(SUCRA: 12.60%), while the effect of atorvastatin alone is minimal(SUCRA: 80.60%). From the numerical results of SUCRA, the intervention order of TC index improvement from high to low is: A(SUCRA: 12.60%) > J(SUCRA: 20.94%) > C(SUCRA: 27.15%) > H(SUCRA: 30.47%) > L(SUCRA: 48.68%) > M(SUCRA: 48.69%) > K(SUCRA: 48.81%) > D(SUCRA: 50.44%) > G(SUCRA: 69.77%) > B(SUCRA: 78.92%) > T(SUCRA: 80.60%) > F(SUCRA: 82.93%)

##### 3.5.1.3 LDL-C

A total of 20 studies reported LDL-C (blood lipid) levels, containing one involving dantian Jiangzhi Granules, one involving danxiang qingzhi granules, one involving hedan tablet, two involving jiangzhi tongluo soft capsule, two involving jiangzhi Tongmai Capsule, two involving pushen capsule, one involving songling xuemaikang capsule, two involving xuezhikang capsule, one involving yindan xinnaotong soft capsule, two involving ginkgo leaf tablet, and five involving zhibitai Capsule. We combined the effect sizes of 19 studies. Compared with atorvastatin alone, A’s MD= -1.90(95%CI: [-2.27, -1.54]), B’s MD= -0.18(95%CI: [-0.68, 0.33]), C’s MD= -8.13(95%CI: [-9.70, -6.57]), D’s MD= -0.56(95%CI: [-0.92, -0.21]), F’s MD= -0.02(95%CI: [-0.38,0.34]). G’s MD= -0.43 (95% CI: [-0.79, -0.08]), H’s MD= -3.14(95%CI: [-3.73, -2.55]), J’s MD= -0.94(95%CI: [-1.92, 0.04]), K’s MD= -0.29(95%CI: [-0.58, -0.00]), L’s MD= -0.56(95%CI: [-1.22, 0.09]), M’s MD= -1.15(95%CI: [-2.02, -0.28]). Except for B, F, J, L, other research results showed statistical significance. Among them, the use of C may have the greatest impact on reducing LDL-C indicators, and the area under the LDL-C level curve after treatment (SUCRA: 3.37%), while the effect of atorvastatin alone is minimal (SUCRA: 84.22%). From the numerical results of SUCRA, the intervention order of LDL-C index improvement from high to low is: C(SUCRA: 3.37%) > A(SUCRA: 13.97%) > H(SUCRA: 26.15%) > M(SUCRA: 47.64%) > D(SUCRA: 47.86%) > J(SUCRA: 48.54%) > L(SUCRA: 50.76%) > B(SUCRA: 65.25%) > G(SUCRA: 66.90%) > K(SUCRA: 67.41%) > F(SUCRA: 77.91%) > T(SUCRA: 84.22%).

##### 3.5.1.4 HDL-C

A total of 21 studies reported HDL-C (blood lipid) levels, including 1 study using danxiang qingzhi granules, 1 study using hedan tablet, 3 studies using jiangzhi tongluo soft capsule, 2 studies using jiangzhi Tongmai Capsule, 2 studies using pushen capsule, 1 study using songling xuemaikang capsule, 3 studies using xuezhikang capsule, 1 study using yindan xinnaotong soft capsule, 2 studies using ginkgo leaf tablet, and 5 studies using zhibitai Capsule. We combined the effect sizes of 20 studies. Compared with atorvastatin alone, B’s MD= 0.19(95%CI: [-0.31,0.70]), C’s MD= 0.56(95%CI: [0.04, 1.07]), D’s MD= 0.58(95%CI: [0.32, 0.84]), F’s MD= 0.08(95%CI: [-0.28, 0.44]), G’s MD= 1.31(95%CI: [-1.11, 3.74]), H’s MD= 2.53(95%CI: [2.00, 3.06]). J’s MD= 1.61(95%CI: [0.82, 2.39]), K’s MD= 0.70(95%CI: [0.41, 1.00]), L’s MD= 0.07(95%CI: [-1.93, 2.06]), M’s MD= 0.53(95%CI: [0.12, 0.94]). Except for B, F, G, L, other research results showed statistical significance. Among them, the use of J may have the greatest impact on the increase of HDL-C index, and the area under the curve of HDL-C level after treatment (SUCRA: 79.87%), while the effect of atorvastatin alone is the smallest(SUCRA: 22.97%). From the numerical results of SUCRA, the intervention order of HDL-C index improvement from high to low is: J(SUCRA: 79.87%) > H(SUCRA: 74.48%) > G(SUCRA: 66.98%) > D(SUCRA: 66.51%) > C(SUCRA: 54.95%) > K(SUCRA: 49.53%) > M(SUCRA: 43.03%) > B(SUCRA: 35.84%) > F(SUCRA: 30.04%) > L(SUCRA: 25.79%) > T(SUCRA: 22.97%).

#### 3.5.2 Secondary outcome

##### 3.5.2.1 Clinical effectiveness

There were 14 studies with clinical effectiveness as the secondary outcome, including one involving Dantian Jiangzhi Granules, one involving hedan tablet, one involving jiangzhi tongluo soft capsule, one involving jiangzhi Tongmai Capsule, one involving pushen capsule, one involving songling xuemaikang capsule, three involving xuezhikang capsule, one involving yindan xinnaotong soft capsule, and four involving zhibitai Capsule. Compared with using atorvastatin alone, the combined effect of Chinese patent medicine combined with atorvastatin intervention was better (RR= 1.18, 95%CI: [1.13, 1.23]). The combination of these Chinese patent medicines and atorvastatin could improve the clinical effectiveness of patients with hyperlipidemia, and the difference was statistically significant. From the numerical results of SUCRA, the order of clinical effectiveness improvement from high to low is: C(SUCRA: 80.36%) > H(SUCRA: 75.67%) > D(SUCRA: 57.44%) > M(SUCRA: 57.38%) > F(SUCRA: 54.07%) > A(SUCRA: 51.96%) > K(SUCRA: 47.53%) > J(SUCRA: 41.29%) > G(SUCRA: 29.64%) > T(SUCRA: 4.66%).

##### 3.5.2.2 Adverse reactions

Adverse reaction data were identified for a total of 17 studies. However, due to the combined reporting of adverse events from both the treatment and control groups, or unclear reporting of specific adverse events, we were unable to perform a NMA. Instead, all studies that reported adverse reactions are summarized in **Table 1**. In studies that differentiated adverse reactions between the treatment group and the control group, 40 cases of adverse reactions were observed in the treatment group, with an incidence rate of 5.59%. In the control group, 79 cases of adverse reactions occur, with an incidence rate of 11.05%. These results suggest that the combination of Chinese patent medicine with atorvastatin led to a lower incidence of adverse reactions, approximately half the rate, compared to atorvastatin alone.

### 3.6 Publication bias

**Figure 6:**
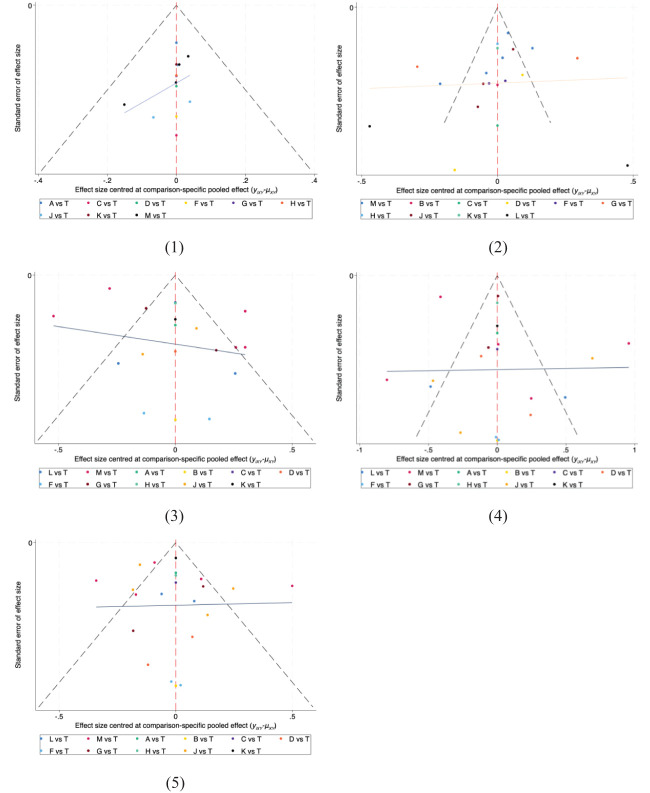
The funnel plot for all outcomes. (1) Clinical effectiveness; (2) HDL-C; (3) LDL-C; (4) TC; (5) TG. A, dantian jiangzhi pill, B, danxiang qingzhi granules, C, hedan tablet, D, jiangzhi tongluo soft capsule, F, Jiangzhi Tongmai Capsule, G, pushen capsule, H, songling xuemaikang capsule, J, xuezhikang capsule, K, yindan xinnaotong soft capsule, L, ginkgo leaf tablet, M, Zhibitai Capsule, T, atorvastatin.

The funnel plots for all results are displayed in **Figure 6**. The results of the Egger and Begger conducted using STATA were as follows: for clinical effectiveness, the Egger test yielded P=0.0055 and the Begg test P=0.0086; for TC level, the Egger test P=0.0002 and the Begg test P=0.0571; for TG levels, the Egger test P=0.0000 and the Begg test P=0.0040; for HDL-C level, the Egger test P=0.0919 and the Begg test P=0.1834; and for LDL-C level, the Egger test P=0.0000 and the Begg test P=0.0252.

With the exception of HDL-C levels, where P values were greater than 0.05, the rest of the Egger test results indicated P<0.05, suggesting potential publication bias in the study. This bias is likely due to the fact that many of the included studies were small sample studies.

## 4 Discussion

Dyslipidemia is one of the most important pathogenic risk factors in the occurrence and development of atherosclerotic cardiovascular disease (ASCVD) (52, 53). Lipid-regulating drugs play an important role in the primary and secondary prevention of ASCVD. Dyslipidemia is often accompanied by other diseases or pathological conditions, such as diabetes, chronic kidney disease, etc., which means it is necessary to make timely and rational medication. With the changes of dietary structure of Chinese residents, the incidence of hyperlipidemia is on the rise. *Zhao* et al. (54) applied the standard of “*Guidelines for the Prevention and Treatment of Dyslipidemia in Chinese Adults (2016 Revision)* “ to reanalyze the DYSIS-CHINA database. The results (54) showed that the proportion of high-risk and extremely high-risk patients with dyslipidemia in China was as high as 96.6%, and the overall LDL-C compliance rate was ≤ 40%. For patients with dyslipidemia in China, it is difficult to achieve the target value of blood lipids through statin monotherapy alone (55). TCM, characterized by multi-pathway, multi-target, and low toxicity, has unique effectiveness in treating hyperlipidemia and is widely recognized. The combination of TCM and statins can often enhance the therapeutic effect and improve patients’ clinical symptoms.

A network meta-analysis was conducted to evaluate the effectiveness and safety of eleven Chinese patent medicines combined with atorvastatin in patients with hyperlipidemia. The research result demonstrated that this combined medication approach exhibited superior effectiveness compared to atorvastatin alone, while effectively mitigating adverse reactions. This study screened 23 randomized controlled trials from multiple databases, involving 2184 patients with hyperlipidemia.

The study found that the combination of Hedan (whether tablet or capsule) and atorvastatin had the best clinical effectiveness, particularly in reducing low-density lipoprotein cholesterol and triglyceride levels. The SUCRA values for LDL-C and TG levels after treatment were 3.37% and 0.33%, respectively. The research mechanism of Hedan tablets in treating dyslipidemia mainly involves regulating glucose metabolism, regulating fat metabolism, improving blood inflammatory response, alleviating oxidative stress and so on. The active ingredient nuciferine in Hedan tablets can increase the activity of lecithin cholesterol acyltransferase and inhibit the synthesis of cholesterol. Tanshinone IIA can reduce LDL-C synthesis, reduce endogenous cholesterol production. Senna extract can promote gastrointestinal motility, reduce the absorption of glucose in the intestine, reduce blood glucose, reduce body weight, and improve insulin resistance (56). Dantian Jiangzhi Granules had the best effect in reducing total cholesterol, and the SUCRA value of TC level after treatment was 12.6%. Modern pharmacological studies have confirmed that Dantian Jiangzhi Pill can improve lipid metabolism, reduce blood lipids, reduce blood viscosity, soften blood vessels, improve microcirculation, strengthen heart and expand blood vessels, and resist platelet aggregation (57). Xuezhikang capsule can significantly improve the level of HDL cholesterol, and the SUCRA value of HDL-C after treatment was 79.87%. Xuezhikang capsule has a wide range of effects, including resisting vascular inflammation, reducing the expression of cell adhesion molecules, cutting off the adhesion of vascular endothelium and monocytes, so that it can enter the subcutaneous space (58), preventing the formation of foam cells and the release of inflammatory cytokines, and reducing CRP levels (59).

However, it is crucial to acknowledge that current real-world studies on TCM combined with atorvastatin for hyperlipidemia treatment mainly rely on small sample sizes RCTs, which may cause some problems. Firstly, the results from small sample studies may lack stability and are susceptible to randomness. Secondly, such studies may not to adequately represent the treatment response and disease progression in a larger patient population, thereby limiting the universality and representativeness of their findings. Finally, due to the limited sample size, certain potential therapeutic effects or adverse reactions may remain undetected, potentially impacting the refinement and enhancement of treatment strategies. We anticipate that more large-scale real-world studies will emerge in the future to improve the reliability and representativeness of research results. Furthermore, we hope for continuous improvement and innovation in research methodologies, such as incorporating more advanced data analysis and processing tools, to better explore and leverage real-world clinical data. This will further enhance the ability of TCM to treat patients with hyperlipidemia, paving the way for personalized and precision medical services.

This study also has some limitations. In terms of the methodological evaluation of the 23 studies included, we found that most of the studies did not mention whether blinding was used for patients and outcome assessors, which may lead to bias and lack of objectivity. The small sample sizes of some studies on Chinese patent medicine have reduced the stability and accuracy of the results. Furthermore, there is limited significance in the evaluation of comparisons between different TCMs. This may be attributed to the fact that some TCMs are composed of multiple herbal ingredients, with complex interactions and synergistic effects among them. In addition, as traditional Chinese medicine emphasizes syndrome differentiation and individualized treatment, people with different constitutions and different symptoms may not have similar effects using the same Chinese patent medicine, and the evaluation criteria are different, resulting in a lower evaluation significance. What should be mentioned is that compared with RCTs of western medicine, the research methods and evaluation system of Chinese patent medicine are still not fully developed, lacking support from large-scale, high-quality clinical trial data. Therefore, we recommend more methodologically rigorous and multi-centered RCTs of Chinese patent medicines combined with atorvastatin in the treatment of hyperlipidemia should be implemented in the future.

## 5 Conclusion

In summary, the combination of Chinese patent medicines with atorvastatin hold great promise in managing hyperlipidemia, with Hedan tablet/capsule showing significant lipid-lowering effects and overall advantages. However, the study emphasizes the need for larger scale randomized controlled trials and in-depth pharmacological studies to further validate these findings. The integrative approach of combining Chinese patent medicines and Western medicine has gained broad recognition in clinical practice, as it can enhance lipid-lowering effect while tailoring treatment to individual patient conditions. This approach not only optimizes patient outcomes but also reduces the side effects associated with Western medicine.

## Data Availability

The original contributions presented in the study are included in the article/**Supplementary Material**, further inquiries can be directed to the corresponding author.
