# Supplementary material for: Effectiveness and safety of eleven Chinese patent medicines combined with atorvastatin in the treatment of hyperlipidemia: a network meta-analysis of randomized controlled trials
Source: Front Endocrinol (Lausanne). 2025 Mar 24;16:1523553. doi: 10.3389/fendo.2025.1523553 (PMC11973096; doi:10.3389/fendo.2025.1523553)
Supplement: Supplementary file 2 [file DataSheet2.docx]

**Supplement 2**

Table 1,The search strategy for the respective database.

| Database name | Search strategies |
| --- | --- |
| CNKI | TKA%=('高脂血症'+'高胆固醇血症'+'高脂蛋白血症'+'高甘油三酯血症'+'血脂过多'+'高脂血'+'血脂异常') AND TKA%=('血脂康'+'脂必妥'+'脂必泰'+'丹香清脂'+'荷丹'+'化滞柔肝'+'降脂灵'+'降脂通脉'+'绞股蓝总甙'+'绞股蓝总苷'+'壳脂'+'蒲参'+'血脂平'+'血滞通'+'丹田降脂'+'脂康'+'松龄血脉康'+'银丹心脑通'+'降脂通络'+'银杏叶'+'丹灯通脑') AND TKA%=('阿托伐他汀'+'立普妥') AND FT%=('随机'+'对照') |
| Wanfang | 主题:("高脂血症" or "高胆固醇血症" or "高脂蛋白血症" or "高甘油三酯血症" or "血脂过多" or "高血脂" or "血脂异常") and 主题:("血脂康" or "脂必妥" or "脂必泰" or "丹香清脂" or "荷丹" or "化滞柔肝" or "降脂灵" or "降脂通脉" or "绞股蓝总甙" or "绞股蓝总苷" or "壳脂" or "蒲参" or "血脂平" or "血滞通" or "丹田降脂" or "脂康" or "松龄血脉康" or "银丹心脑通" or "降脂通络" or "银杏叶" or "丹灯通脑") and 主题:("阿托伐他汀" or "立普妥") and 全部:("随机" or "对照") |
| VIP | (M=(高脂血症 or 高胆固醇血症 or 高脂蛋白血症 or 高甘油三酯血症 or 血脂过多 or 高血脂 or 血脂异常) OR K=(高脂血症 or 高胆固醇血症 or 高脂蛋白血症 or 高甘油三酯血症 or 血脂过多 or 高血脂 or 血脂异常)) and (M=(血脂康 or 脂必妥 or 脂必泰 or 丹香清脂 or 荷丹 or 化滞柔肝 or 降脂灵 or 降脂通脉 or 绞股蓝总甙 or 绞股蓝总苷 or 壳脂 or 蒲参 or 血脂平 or 血滞通 or 丹田降脂 or 脂康 or 松龄血脉康 or 银丹心脑通 or 降脂通络 or 银杏叶 or 丹灯通脑) OR K=(血脂康 or 脂必妥 or 脂必泰 or 丹香清脂 or 荷丹 or 化滞柔肝 or 降脂灵 or 降脂通脉 or 绞股蓝总甙 or 绞股蓝总苷 or 壳脂 or 蒲参 or 血脂平 or 血滞通 or 丹田降脂 or 脂康 or 松龄血脉康 or 银丹心脑通 or 降脂通络 or 银杏叶 or 丹灯通脑)) and (M=(阿托伐他汀 or 立普妥) OR K=(阿托伐他汀 or 立普妥)) and U=(随机 or 对照) |
| SinoMed | #1：高脂血症[不加权:扩展]  #2：高脂血症[常用字段:智能] OR 高胆固醇血症[常用字段:智能] OR 高脂蛋白血症[常用字段:智能] OR 高甘油三酯血症[常用字段:智能] OR 血脂过多[常用字段:智能] OR 高血脂[常用字段:智能] OR 血脂异常[常用字段:智能]  #3：#1 OR #2  #4：血脂康[常用字段:智能] OR 脂必妥[常用字段:智能] OR 脂必泰[常用字段:智能] OR 丹香清脂[常用字段:智能] OR 荷丹[常用字段:智能] OR 化滞柔肝[常用字段:智能] OR 降脂灵[常用字段:智能] OR 降脂通脉[常用字段:智能] OR 绞股蓝总甙[常用字段:智能] OR 绞股蓝总苷[常用字段:智能] OR 壳脂[常用字段:智能] OR 蒲参[常用字段:智能] OR 血脂平[常用字段:智能] OR 血滞通[常用字段:智能] OR 丹田降脂[常用字段:智能] OR 脂康[常用字段:智能] OR 松龄血脉康[常用字段:智能] OR 银丹心脑通[常用字段:智能] OR 降脂通络[常用字段:智能] OR 银杏叶[常用字段:智能] OR 丹灯通脑[常用字段:智能]  #5：阿托伐他汀[常用字段:智能] OR 立普妥[常用字段:智能]  #6：随机[全部字段:智能] OR 对照[常用字段:智能]  #7：#3 AND #4 AND #5 AND #6 |
| PubMed | #1：(Hyperlipidemias[MeSH Terms]) OR (Hyperlipemia) OR (Hyperlipemias) OR (Hyperlipidemia) OR (Lipidemia) OR (Lipidemias) OR (Lipemia) OR (Lipemias) OR (Hypercholesterolemias) OR (High Cholesterol Levels) OR (Cholesterol Level, High) OR (Cholesterol Levels, High) OR (High Cholesterol Level) OR (Level, High Cholesterol) OR (Levels, High Cholesterol) OR (Elevated Cholesterol) OR (Cholesterol, Elevated) OR (Cholesterols, Elevated) OR (Elevated Cholesterols) OR (Hypercholesteremia) OR (Hypercholesteremias)  #2：(Xuezhikang OR Zhibituo OR Zhibitai OR Danxiangqingzhi OR Hedan OR Huazhirougan OR Jiangzhiling OR Jiangzhitongmai OR gypenosides OR gypenosides OR shell fat OR Pushen OR Xuezhiping OR Dantianjiangzhi OR Songlingxuemaikang OR Yindanxinnaotong OR Jiangzhitongluo OR Yinxingye OR Dandengtongnao)  #3：(Atorvastatin[MeSH Terms]) OR ((3R,5R)-7-(2-(4-Fluorophenyl)-5-isopropyl-3-phenyl-4-(phenylcarbamoyl)-1H-pyrrol-1-yl)-3,5-dihydroxyheptanoic acid) OR (Atorvastatin Calcium) OR (Atorvastatin, Calcium Salt) OR (Liptonorm) OR (Lipitor) OR (Atorvastatin Calcium Hydrate) OR (Atorvastatin Calcium Anhydrous) OR (CI 981) OR (CI-981) OR (CI981) OR (Atorvastatin Calcium Trihydrate)  #4：(Randomized Controlled Trial [Publication Type]) OR (Randomized Controlled Trials as Topic[MeSH Terms]) OR (Randomized Controlled Trials)  #5：#1 AND #2 AND #3 AND #4 |
| Cochrane Library | #1 MeSH descriptor: [Hyperlipidemias] explode all trees  #2 Hyperlipemia OR Hyperlipemias OR Hyperlipidemia OR Lipidemia OR Lipidemias OR Lipemia OR Lipemias OR Hypercholesterolemias OR High Cholesterol Levels OR Cholesterol Level, High OR Cholesterol Levels, High OR High Cholesterol Level OR Level, High Cholesterol OR Levels, High Cholesterol OR Elevated Cholesterol OR Cholesterol, Elevated OR Cholesterols, Elevated OR Elevated Cholesterols OR Hypercholesteremia OR Hypercholesteremias  #3 #1 OR #2  #4 Xuezhikang OR Zhibituo OR Zhibitai OR Danxiangqingzhi OR Hedan OR Huazhirougan OR Jiangzhiling OR Jiangzhitongmai OR gypenosides OR gypenosides OR shell fat OR Pushen OR Xuezhiping OR Dantianjiangzhi OR Songlingxuemaikang OR Yindanxinnaotong OR Jiangzhitongluo OR Yinxingye OR Dandengtongnao  #5 Atorvastatin OR Atorvastatin Calcium OR Atorvastatin, Calcium Salt OR Liptonorm OR Lipitor OR Atorvastatin Calcium Hydrate OR Atorvastatin Calcium Anhydrous OR CI 981 OR CI-981 OR CI981 OR Atorvastatin Calcium Trihydrate  #6 random*  #7 #3 AND #4 AND #5 AND #6 |
| Web of Science | #1：TS=(Hyperlipemia OR Hyperlipemias OR Hyperlipidemia OR Lipidemia OR Lipidemias OR Lipemia OR Lipemias OR Hypercholesterolemias OR High Cholesterol Levels OR Cholesterol Level, High OR Cholesterol Levels, High OR High Cholesterol Level OR Level, High Cholesterol OR Levels, High Cholesterol OR Elevated Cholesterol OR Cholesterol, Elevated OR Cholesterols, Elevated OR Elevated Cholesterols OR Hypercholesteremia OR Hypercholesteremias)  #2：TS=(Xuezhikang OR Zhibituo OR Zhibitai OR Danxiangqingzhi OR Hedan OR Huazhirougan OR Jiangzhiling OR Jiangzhitongmai OR gypenosides OR gypenosides OR shell fat OR Pushen OR Xuezhiping OR Dantianjiangzhi OR Songlingxuemaikang OR Yindanxinnaotong OR Jiangzhitongluo OR Yinxingye OR Dandengtongnao)  #3：TS=(Atorvastatin OR Atorvastatin Calcium OR Atorvastatin, Calcium Salt OR Liptonorm OR Lipitor OR Atorvastatin Calcium Hydrate OR Atorvastatin Calcium Anhydrous OR CI 981 OR CI-981 OR CI981 OR Atorvastatin Calcium Trihydrate)  #4：TS=(randomized controlled trial OR randomized OR placebo OR random OR randomised)  #5：#1 AND #2 AND #3 AND #4 |
| Embase | #1 'hyperlipidemia'/exp  #2 'hyperlipidemia'/exp AND [embase]/lim  #3 'hyperlipaemia':ab,ti OR 'hyperlipemia':ab,ti OR 'hyperlipidaemia':ab,ti OR 'hyperlipidaemia type ii':ab,ti OR 'hyperlipidaemia type iii':ab,ti OR 'hyperlipidaemia type v':ab,ti OR 'hyperlipidaemias':ab,ti OR 'hyperlipidemia type ii':ab,ti OR 'hyperlipidemia type iii':ab,ti OR 'hyperlipidemia type v':ab,ti OR 'hyperlipidemias':ab,ti OR 'hyperlipidemic':ab,ti OR 'lipaemia':ab,ti OR 'lipemia':ab,ti OR 'lipidaemia':ab,ti OR 'lipidemia':ab,ti OR 'hyperlipidemia':ab,ti  #4 #1 OR #2 OR #3  #5 'Xuezhikang':ab,ti OR 'Zhibituo':ab,ti OR 'Zhibitai':ab,ti OR 'Danxiangqingzhi':ab,ti OR 'Hedan':ab,ti OR 'Huazhirougan':ab,ti OR 'Jiangzhiling':ab,ti OR 'Jiangzhitongmai':ab,ti OR 'gypenosides':ab,ti OR 'shell fat':ab,ti OR 'Pushen':ab,ti OR 'Xuezhiping':ab,ti OR 'Dantianjiangzhi':ab,ti OR 'Songlingxuemaikang':ab,ti OR 'Yindanxinnaotong':ab,ti OR 'Jiangzhitongluo':ab,ti OR 'Yinxingye':ab,ti OR 'Dandengtongnao':ab,ti  #6 'Atorvastatin':ab,ti OR 'Atorvastatin Calcium':ab,ti OR 'Atorvastatin, Calcium Salt':ab,ti OR 'Liptonorm':ab,ti OR 'Lipitor':ab,ti OR 'Atorvastatin Calcium Hydrate':ab,ti OR 'Atorvastatin Calcium Anhydrous':ab,ti OR 'CI 981':ab,ti OR 'CI-981':ab,ti OR 'CI981':ab,ti OR 'Atorvastatin Calcium Trihydrate':ab,ti  #7 'randomized controlled trial'/exp  #8 'controlled trial, randomized':ab,ti OR 'randomised controlled study':ab,ti OR 'randomised controlled trial':ab,ti OR 'randomized controlled study':ab,ti OR 'trial, randomized controlled':ab,ti OR 'randomized controlled trial':ab,ti  #9 #7 OR #8  #10 #4 AND #5 AND #6 AND #9 |

CNKI: China National Knowledge Infrastructure; SinoMed: the Chinese Biomedical Literature Database; WanFang: the WanFang Database; VIP: the Chinese Scientific Journals Full-Text Database;Embase Database; Excerpta Medica Database; WOS Database: Web of Science Database;PubMed Database: the PubMed Database.
