# Supplementary material for: Effectiveness and safety of eleven Chinese patent medicines combined with atorvastatin in the treatment of hyperlipidemia: a network meta-analysis of randomized controlled trials
Source: Front Endocrinol (Lausanne). 2025 Mar 24;16:1523553. doi: 10.3389/fendo.2025.1523553 (PMC11973096; doi:10.3389/fendo.2025.1523553)
Supplement: Supplementary file 3 [file DataSheet3.docx]

**Supplement 3**

The detailed information of the components, indications, adverse reactions, contraindications, and precautions of these Chinese patent medicines

| **Medicines vocabularies** | **Component** | **Indication** | **adverse reaction** | **Taboo** | **Notice** |
| --- | --- | --- | --- | --- | --- |
| Zhibitai Capsules | Hawthorn、oriental waterplantain rhizome、largehead atractylodes rhizome、fermented red rice | Phlegm and blood stasis, spleen and stomach. It mainly treats hyperlipidemia caused by phlegm and blood stasis and adverse qi and blood. Symptoms include dizziness, chest tightness, abdominal distension, loss of appetite, fatigue, etc. | No obvious adverse reactions | Disabled for pregnant and lactating women | High-fat diet should be avoided during and after medication, such as fat meat, poultry skin, internal organs, egg yolk, etc. |
| Jiangzhi Tongluo Soft Capsules | turmeric extract | Invigorate the circulation of qi, lipid-lowering turbidity. It is used for hyperlipidemia patients with blood stasis and qi stagnation syndrome. The symptoms include chest pain, chest pain, chest tightness, ecchymosis or ecchymosis at the tip of the tongue, stringy or astringent pulse. | Occasionally bloating, diarrhea | remains unclear | remains unclear |
| Jiangzhi Tongmai Capsules | sickle senna seed、common turmeric rhizome、oriental waterplantain rhizome、root of pseudo-ginseng、bermudagrass herb | Phlegm dampness, blood circulation. For hyperlipidemia caused by phlegm and blood stasis block | remains unclear | contraindication in pregnancy | remains unclear |
| Pushen Capsules | tuber fleeceflower root、longbract cattail pollen、root of red rooted salvia、chuanxiong rhizome、common peony root、hawthorn  、oriental waterplantain rhizome、dangshen | Activating blood circulation and removing blood stasis, nourishing yin and removing turbidity. For blood stasis syndrome of hyperlipidemia. Symptoms are dizziness, head tingling, chest tingling, chest tightness, palpitation, limb numbness ; dark purple tongue or petechiae, pulse is fine and astringent | 1.A small number of patients after medication epigastric discomfort, nausea, abdominal distension, diarrhea, anorexia, dry mouth and so on.  2.During the clinical trial, one patient 's BUN was 7.01 mmol / L before treatment and increased to 9.28 mmol / L after treatment, which may be unrelated to the administration of drugs. | remains unclear | remains unclear |
| Xuezhikang Capsules | fermented red rice | Turbidity lipid-lowering, promoting blood circulation to remove blood stasis, spleen digestion. For hyperlipidemia caused by phlegm obstruction and blood stasis, symptoms of shortness of breath, fatigue, dizziness, headache, chest tightness, abdominal distension, poor appetite, etc. ; it can also be used in the adjuvant treatment of cardiovascular and cerebrovascular diseases caused by hyperlipidemia and atherosclerosis. | 1.Generally well tolerated, most of the side effects are mild and transient.  The common adverse reactions of this product are gastrointestinal discomfort, such as stomach pain, abdominal distension, stomach burning, etc.  3 、 Occasionally cause serum aminotransferase and creatine phosphokinase reversible increase.  4. Rare fatigue, dry mouth, dizziness, headache, myalgia, rash, gallbladder pain, edema, conjunctival congestion and urinary tract irritation symptoms. | 1. Allergic to this product  2. Active hepatitis or unexplained elevated serum aminotransferase is prohibited. | 1.During the period of medication, blood lipids, serum aminotransferase and creatine phosphokinase should be examined regularly ; those with a history of liver disease should pay special attention to the monitoring of liver function.  2.In the course of this product treatment, if the serum aminotransferase increased to 3 times the normal high limit, or the serum creatine phosphokinase increased significantly, the product should be discontinued.  3.Pregnant women and lactating women are not recommended.  4. Diet should be light.  The safety and efficacy of pediatric medication have not yet been determined. |
| Yindan Xinnaotong Soft Capsules | ginkgo leaf、root of red rooted salvia、fleabane、gynostemma、hawthorn  、garlic、root of pseudo-ginseng、borneol | Blood circulation, qi pain, digestion and stagnation. For qi stagnation and blood stasis caused by chest pain, chest tightness, shortness of breath, palpitations, etc. ; coronary heart disease angina pectoris, hyperlipidemia, cerebral arteriosclerosis, stroke, stroke sequelae see the above symptoms. | remains unclear | remains unclear | remains unclear |
| Ginkgo Leaf Tablets | ginkgo biloba leaf extract | Promoting blood circulation to remove blood stasis and dredging collaterals. For chest pain caused by blood stasis, stroke, hemiplegia, tongue strong language Jian ; coronary heart disease stable angina pectoris, cerebral infarction see the above syndromes | remains unclear | remains unclear | remains unclear |
| Songling Xuemaikang Capsules | Fresh pine leaves、lobed kudzuvine root、pearl powder | Pinggan Qianyang, calm the heart and tranquilize the mind. It is used for headache, vertigo, irritability, palpitation and insomnia caused by hyperactivity of liver yang ; hypertension and primary hyperlipidemia see the above syndromes | Some patients may have mild diarrhea and epigastric fullness after taking medicine. Taking medicine after meals can help reduce or improve these symptoms. | remains unclear | 1.This product is easy to absorb moisture, after opening the bottle to take the medicine, the bottle cap should be immediately tightened and sealed.  2.If due to improper preservation, resulting in the product moisture absorption caking shape change, please do not continue to use.  3.Please put this product in a place where children can 't touch and see.  4.It is forbidden for those who are allergic to this product. |
| Hedan Tablets | lotus leaf、root of red rooted salvia、hawthorn  、senna leaf、Salt psoralen | Phlegm turbidity, promoting blood circulation to remove blood stasis. For hyperlipidemia is phlegm turbidity with blood stasis syndrome | Occasionally diarrhea, nausea, dry mouth | Spleen and stomach deficiency cold, loose stools are forbidden to take | Pregnant women forbidden. |
| Danxiang Qingzhi Granules | root of red rooted salvia、chuanxiong rhizome、peach seed、odorate rosewood root-wood、commom burreed rhizome、turmeric rhizome、bitter orange、Wine rhubarb | Promoting blood circulation and removing blood stasis, promoting qi and dredging collaterals. It is used for hyperlipidemia patients with qi stagnation and blood stasis syndrome. | Nausea occurred in some patients after taking the medicine, which could be relieved by themselves. | Prohibited for pregnant women and those with bleeding tendency | Those with weak constitution should be used with caution. |
| Dantian Jiangzhi Pills | root of red rooted salvia、root of pseudo-ginseng、tuber fleeceflower root、ginseng、chuanxiong rhizome、oriental waterplantain rhizome、chinese angelica root、siberian solomonseal rhizome、cinnamon、shorthorned epimedium herb、wujiapi | Blood circulation, spleen and kidney, can reduce serum lipids, improve microcirculation. For hyperlipidemia | remains unclear | remains unclear | remains unclear |
