# Supplementary material for: Effectiveness and safety of eleven Chinese patent medicines combined with atorvastatin in the treatment of hyperlipidemia: a network meta-analysis of randomized controlled trials
Source: Front Endocrinol (Lausanne). 2025 Mar 24;16:1523553. doi: 10.3389/fendo.2025.1523553 (PMC11973096; doi:10.3389/fendo.2025.1523553)
Supplement: Supplementary file 4 [file DataSheet4.docx]

**Supplement 4**

Forest map and subgroup analysis


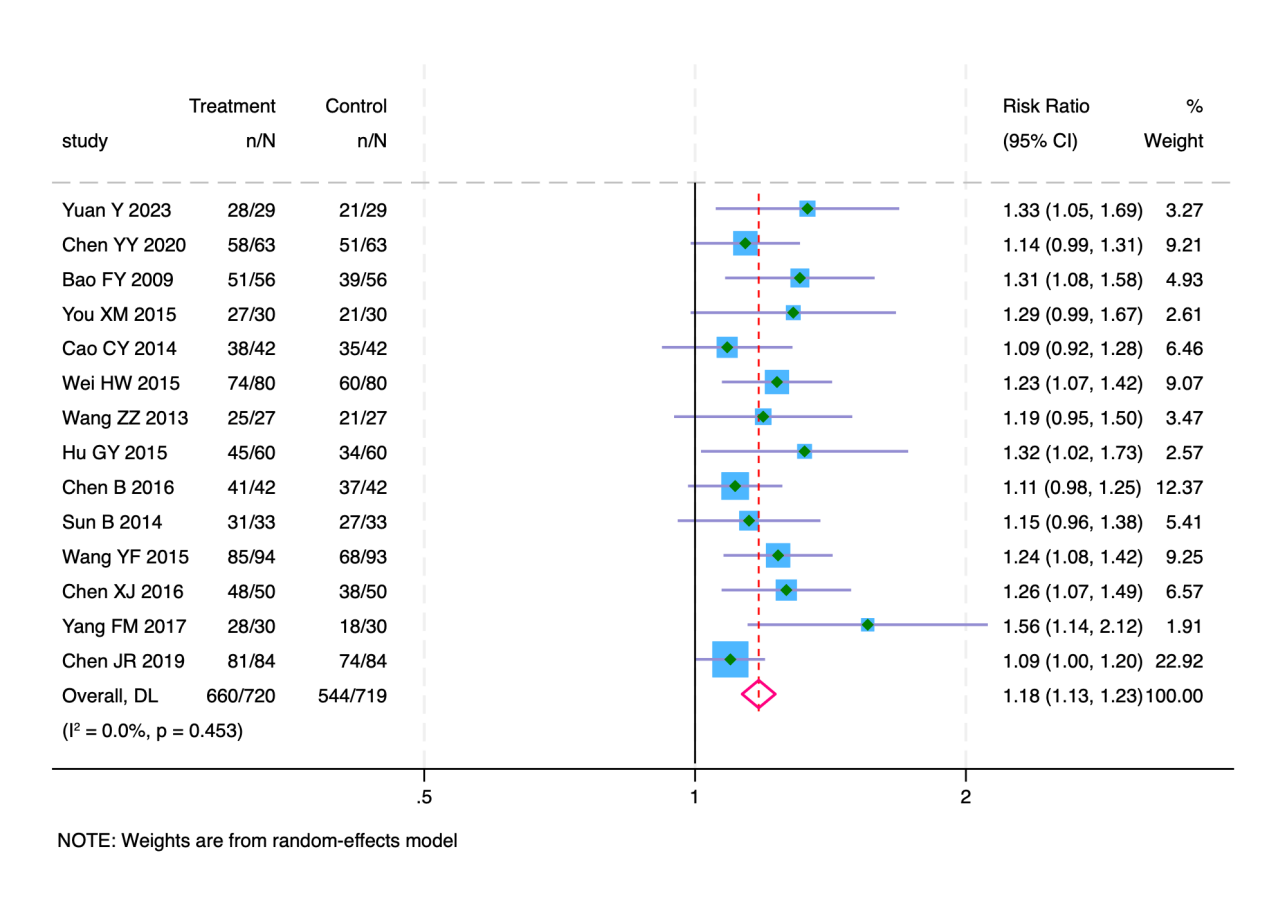


**Figure 1 Forest map and subgroup analysis:Clinical effectiveness**


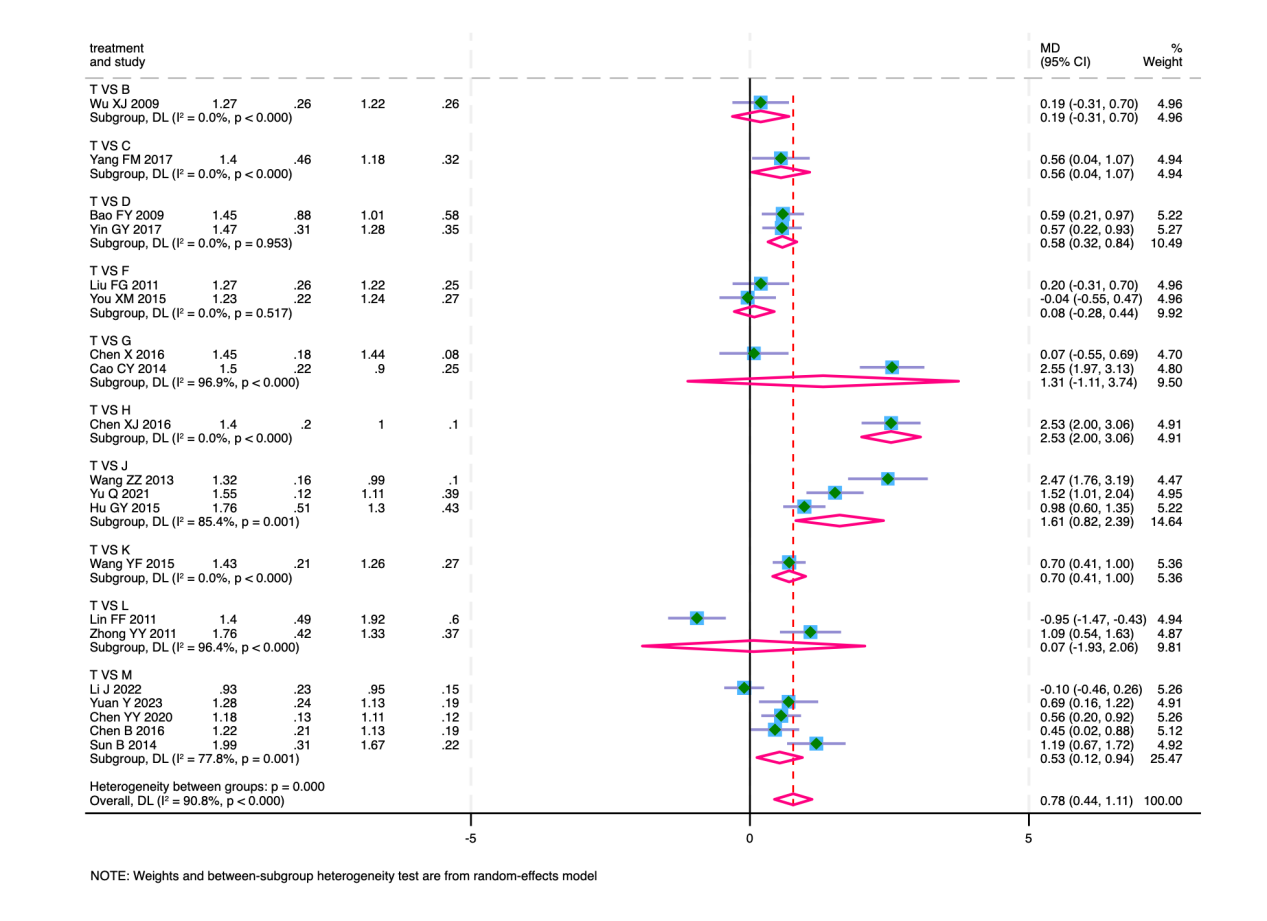
**Figure 2 Forest map and subgroup analysis:HDL-c**


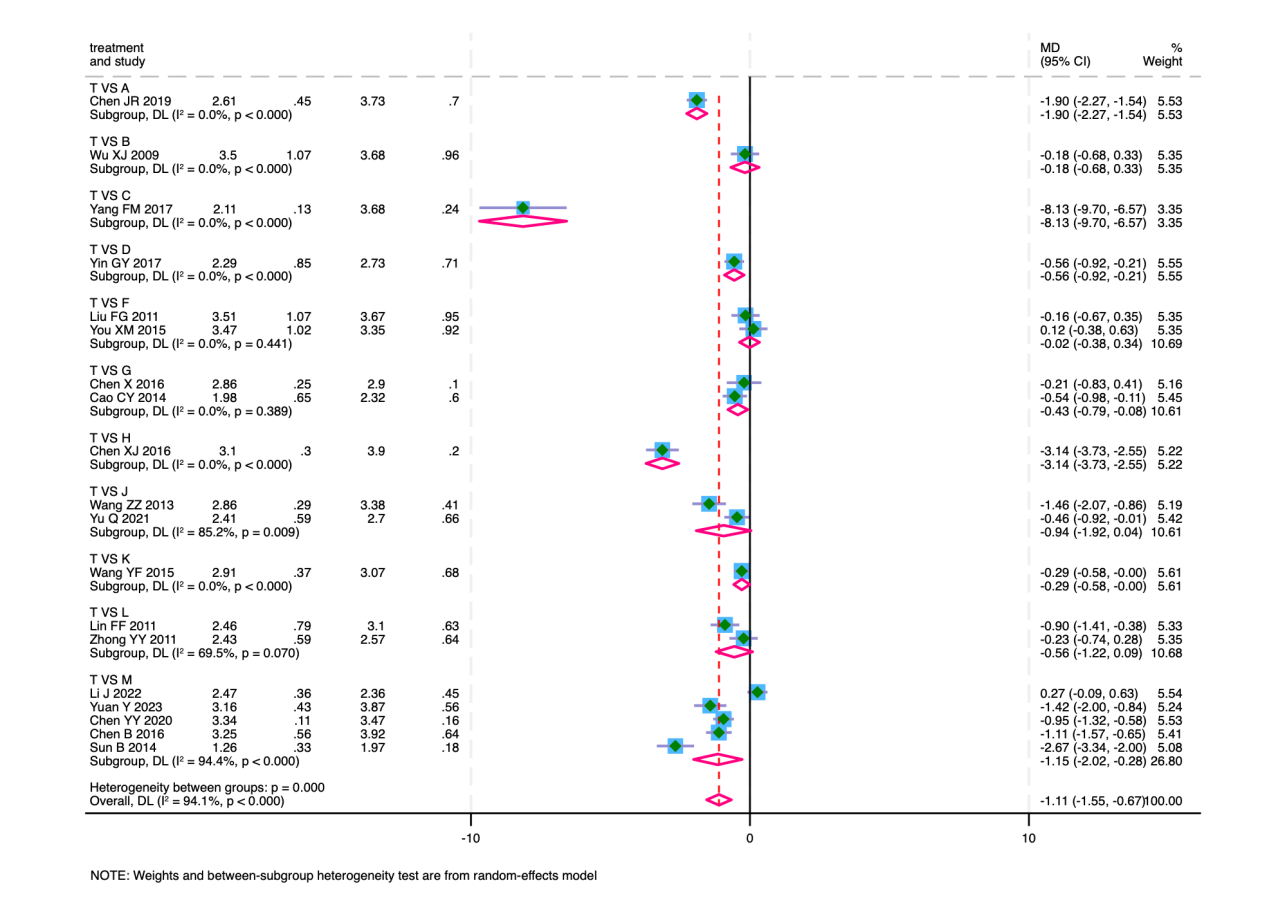


**Figure 3 Forest map and subgroup analysis:LDL-c**


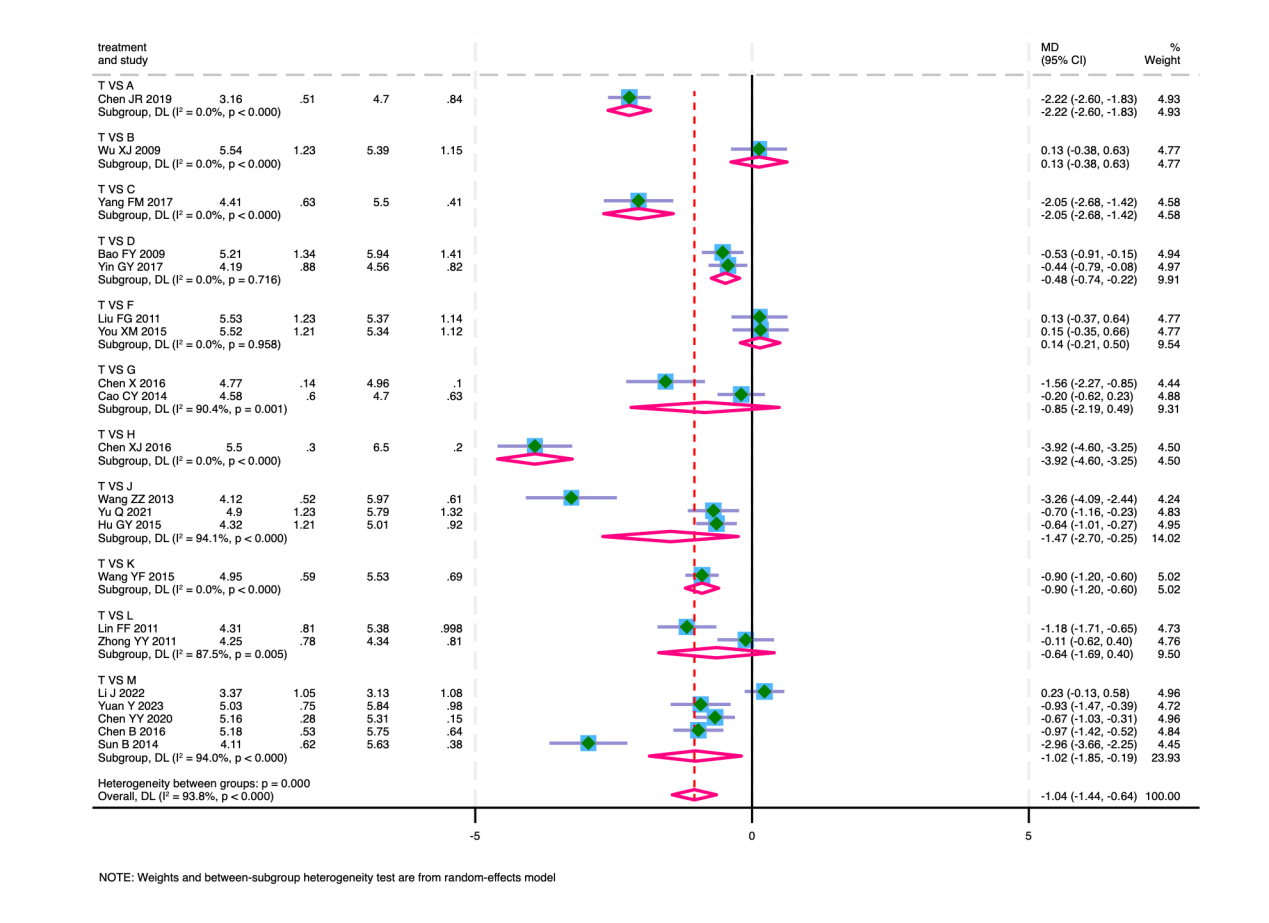


**Figure 4 Forest map and subgroup analysis:TC**


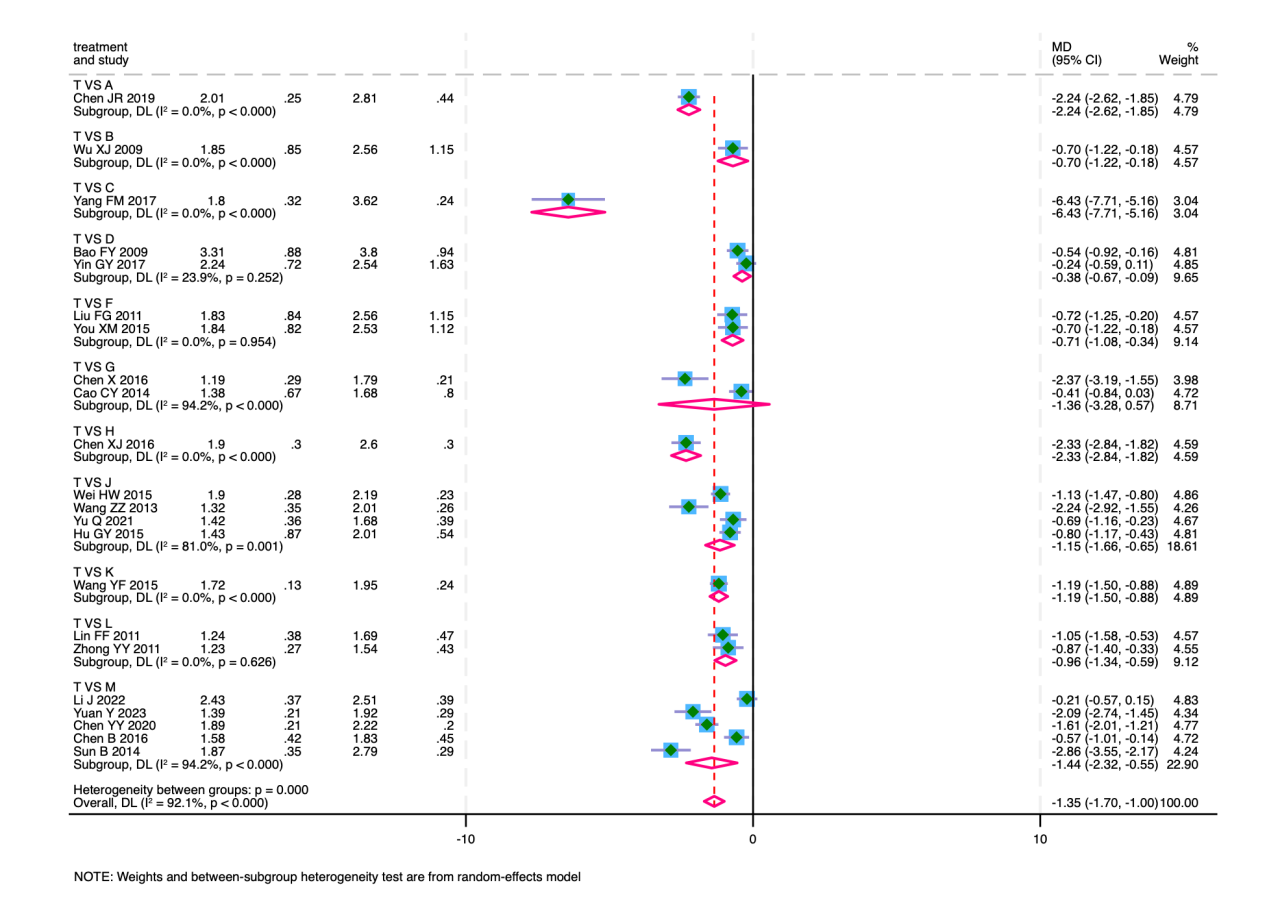
**Figure 5 Forest map and subgroup analysis:TG**
