# Supplementary material for: Effectiveness and safety of eleven Chinese patent medicines combined with atorvastatin in the treatment of hyperlipidemia: a network meta-analysis of randomized controlled trials
Source: Front Endocrinol (Lausanne). 2025 Mar 24;16:1523553. doi: 10.3389/fendo.2025.1523553 (PMC11973096; doi:10.3389/fendo.2025.1523553)
Supplement: Supplementary file 5 [file DataSheet5.docx]

**Supplement 5**

Sensitivity analysis


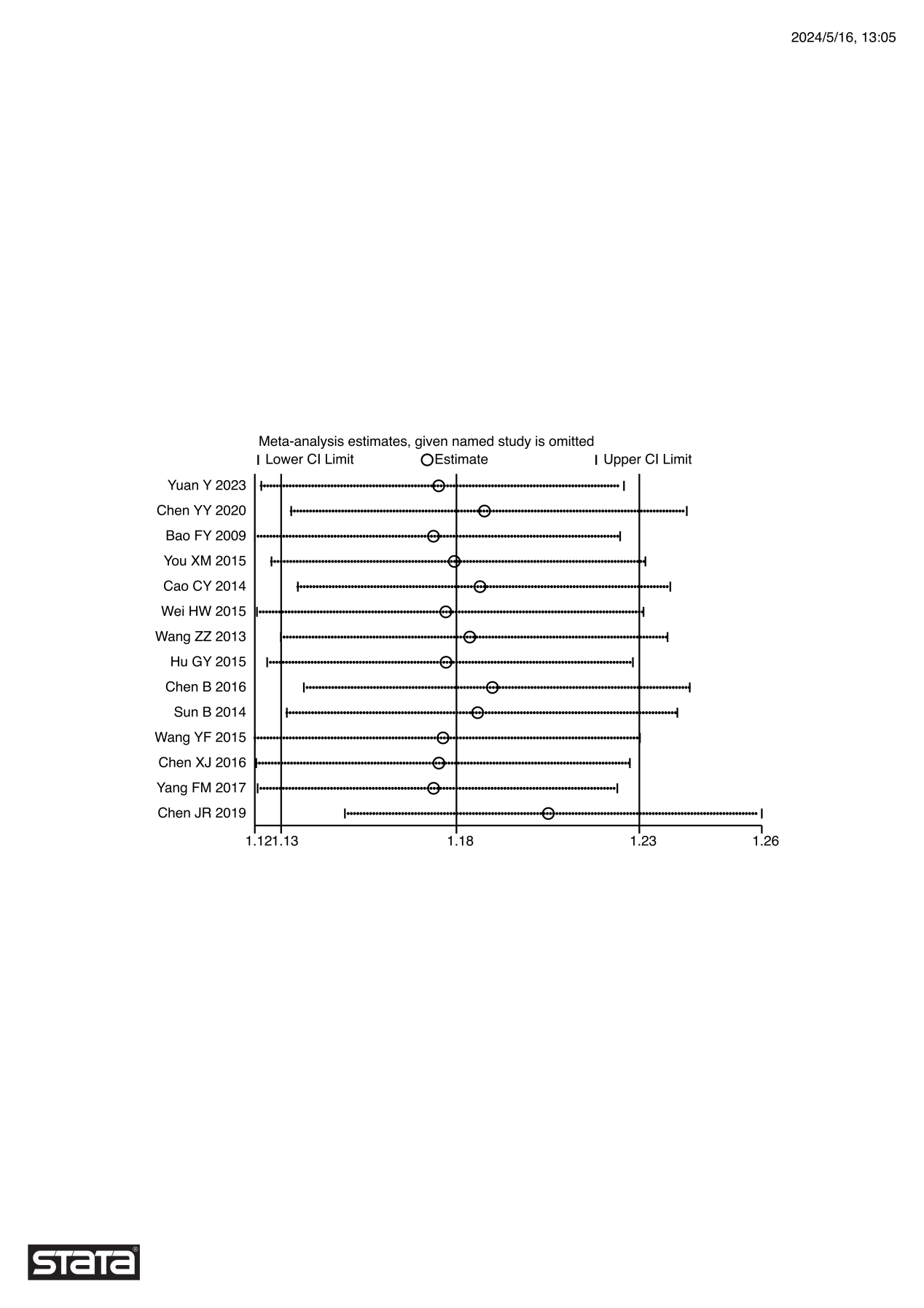


**Figure 1 Sensitivity analysis:Clinical effectiveness**


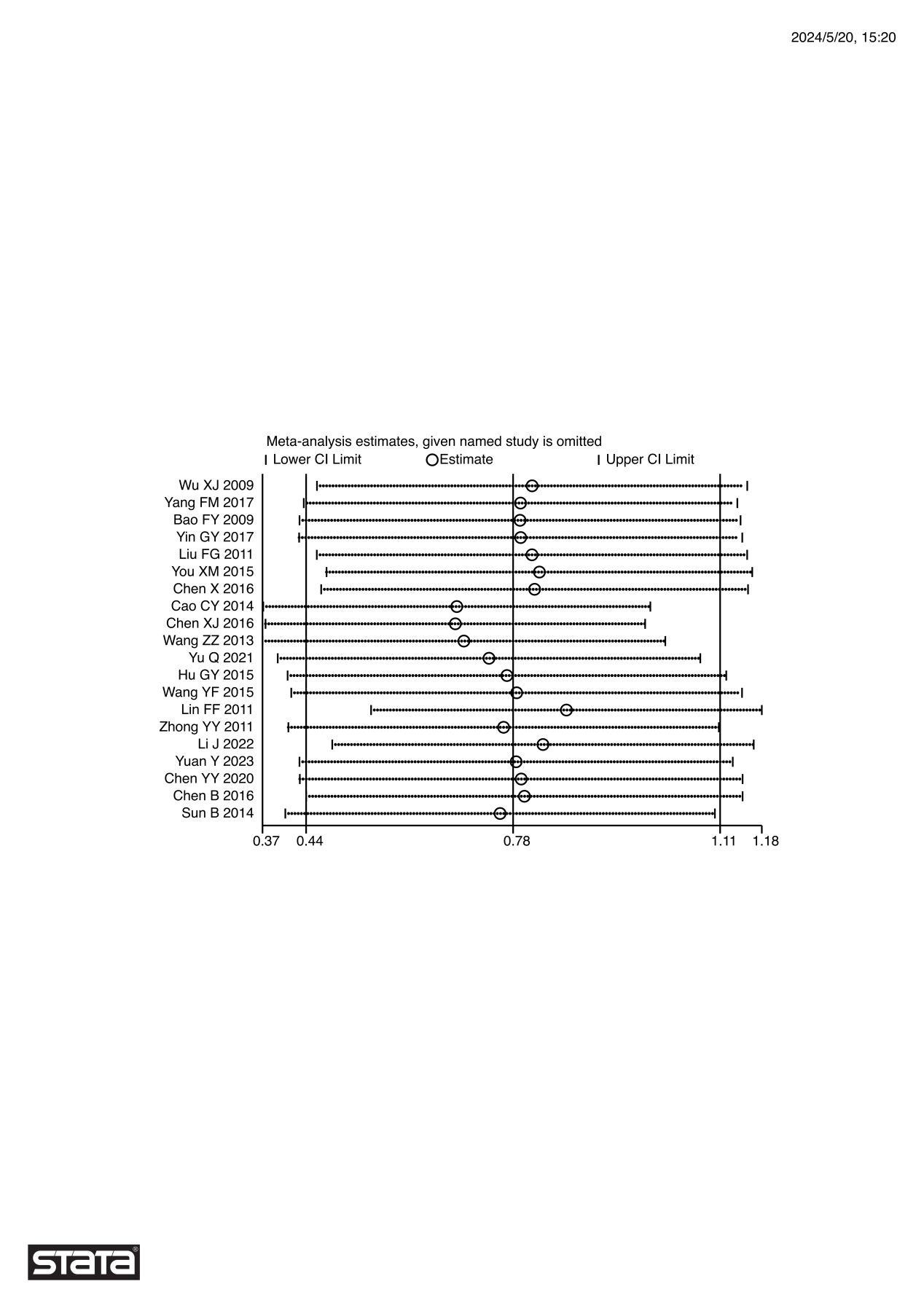


**Figure 2 Sensitivity analysis:HDL-c**


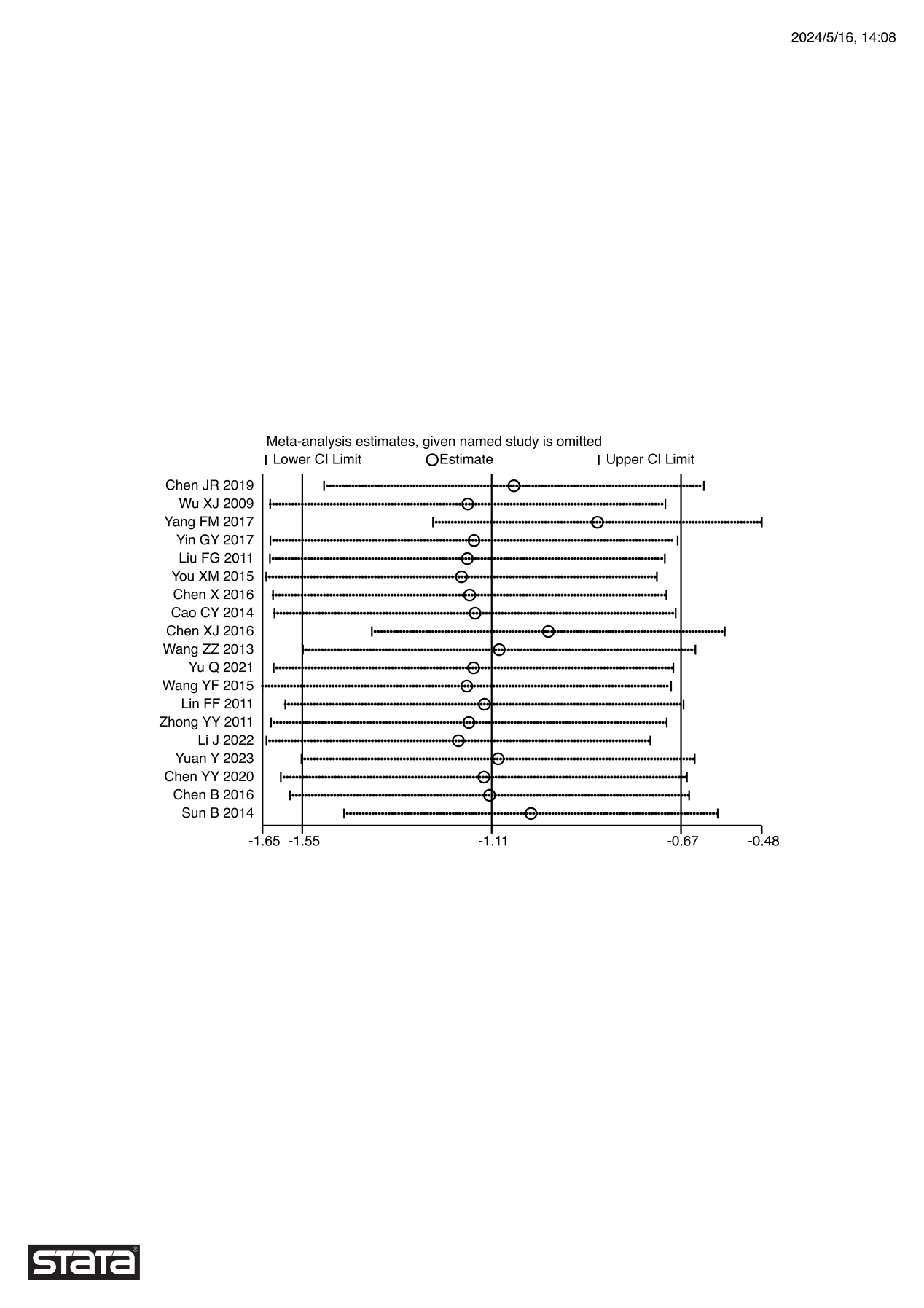


**Figure 3 Sensitivity analysis:LDL-c**


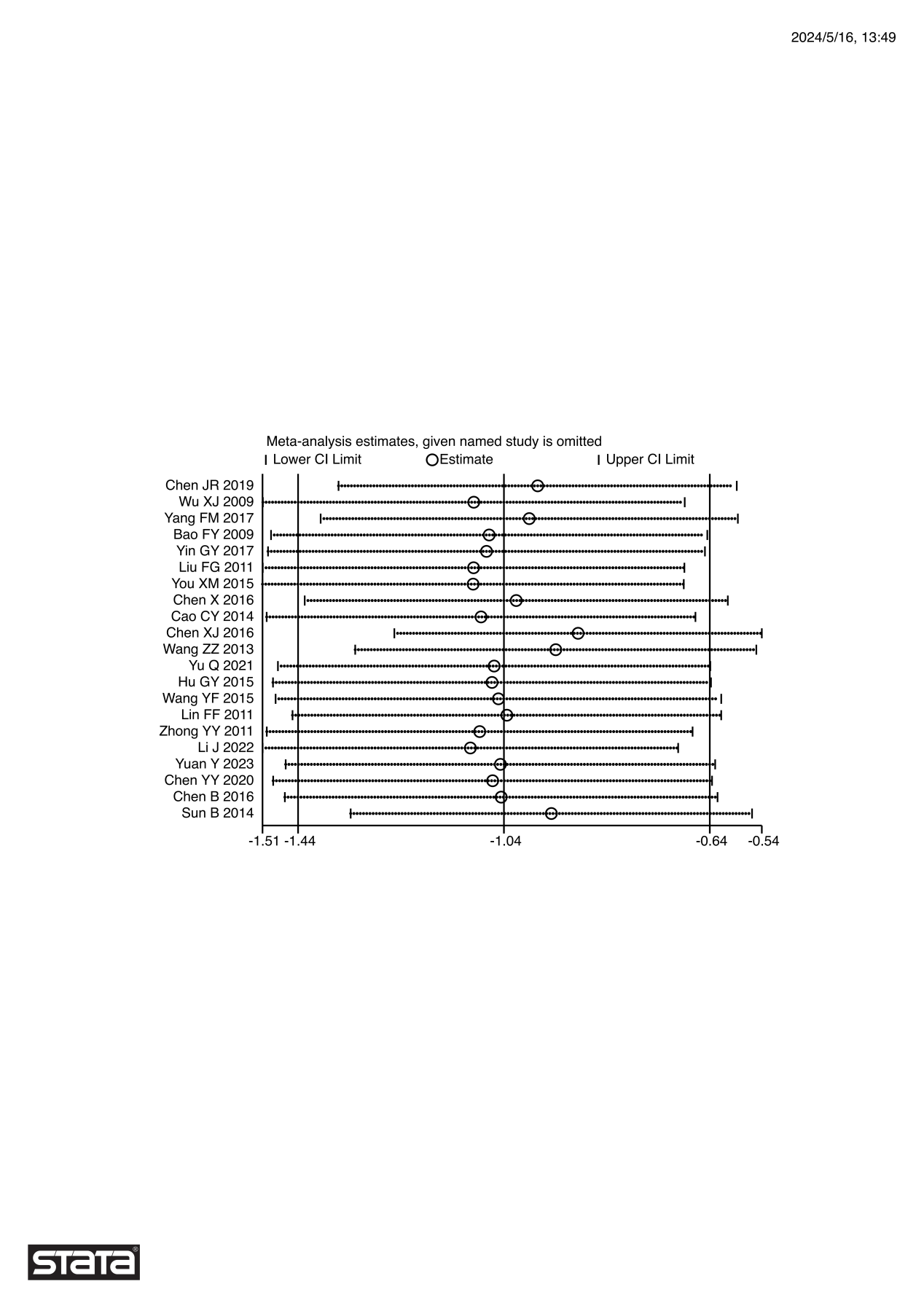


**Figure 4 Sensitivity analysis:TC**


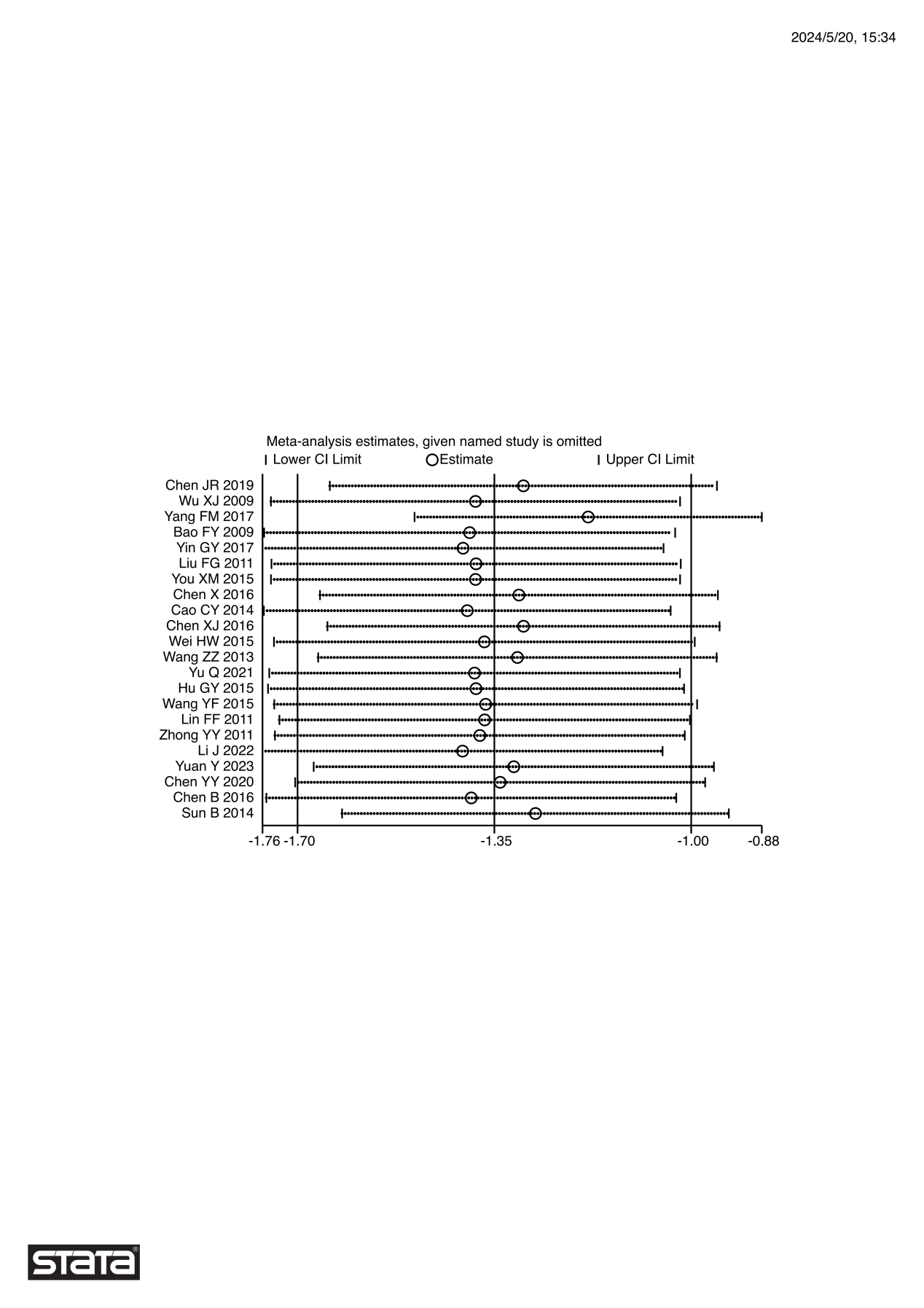


**Figure 5 Sensitivity analysis:TG**
