# Supplementary material for: Effectiveness and safety of eleven Chinese patent medicines combined with atorvastatin in the treatment of hyperlipidemia: a network meta-analysis of randomized controlled trials
Source: Front Endocrinol (Lausanne). 2025 Mar 24;16:1523553. doi: 10.3389/fendo.2025.1523553 (PMC11973096; doi:10.3389/fendo.2025.1523553)
Supplement: Supplementary file 6 [file DataSheet6.docx]

**Supplement 6**

Trajectory diagram and density diagram


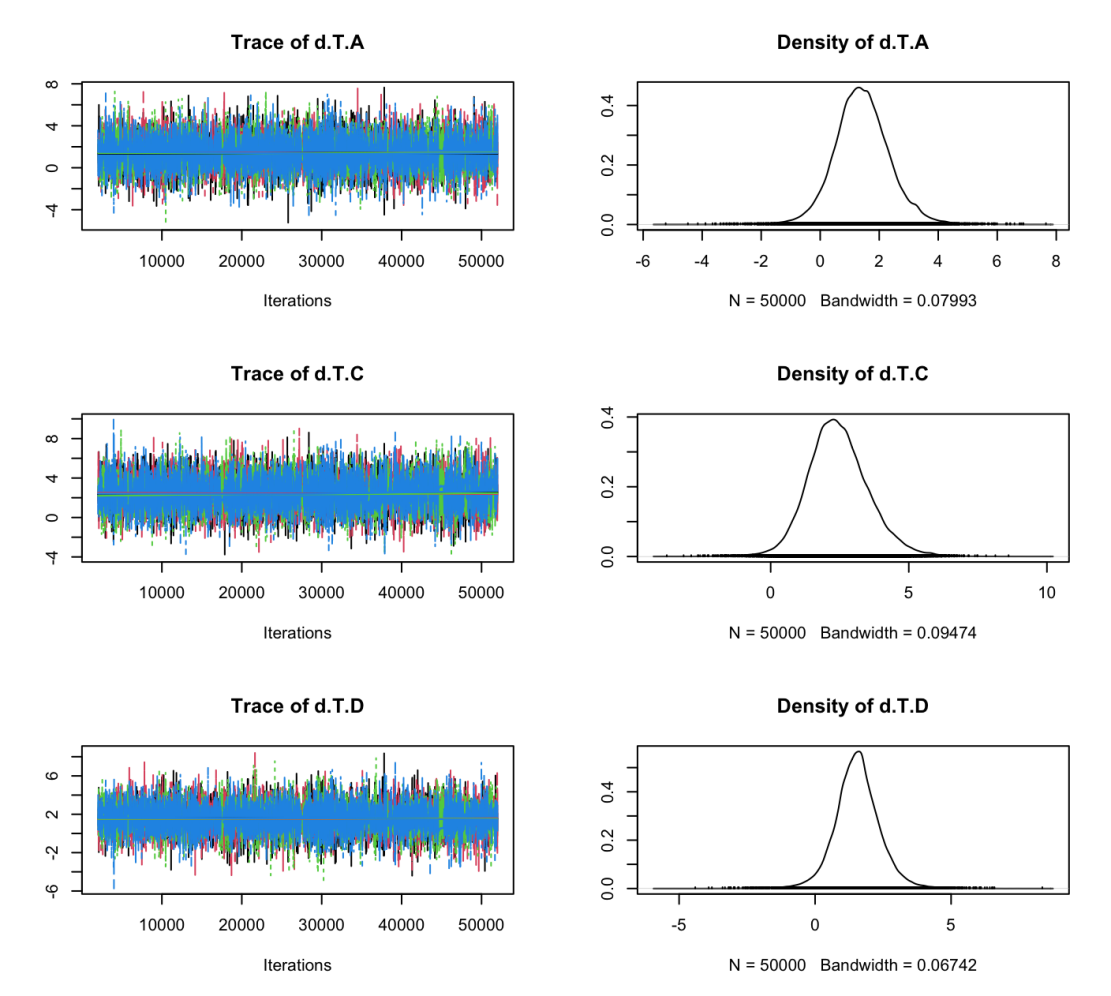

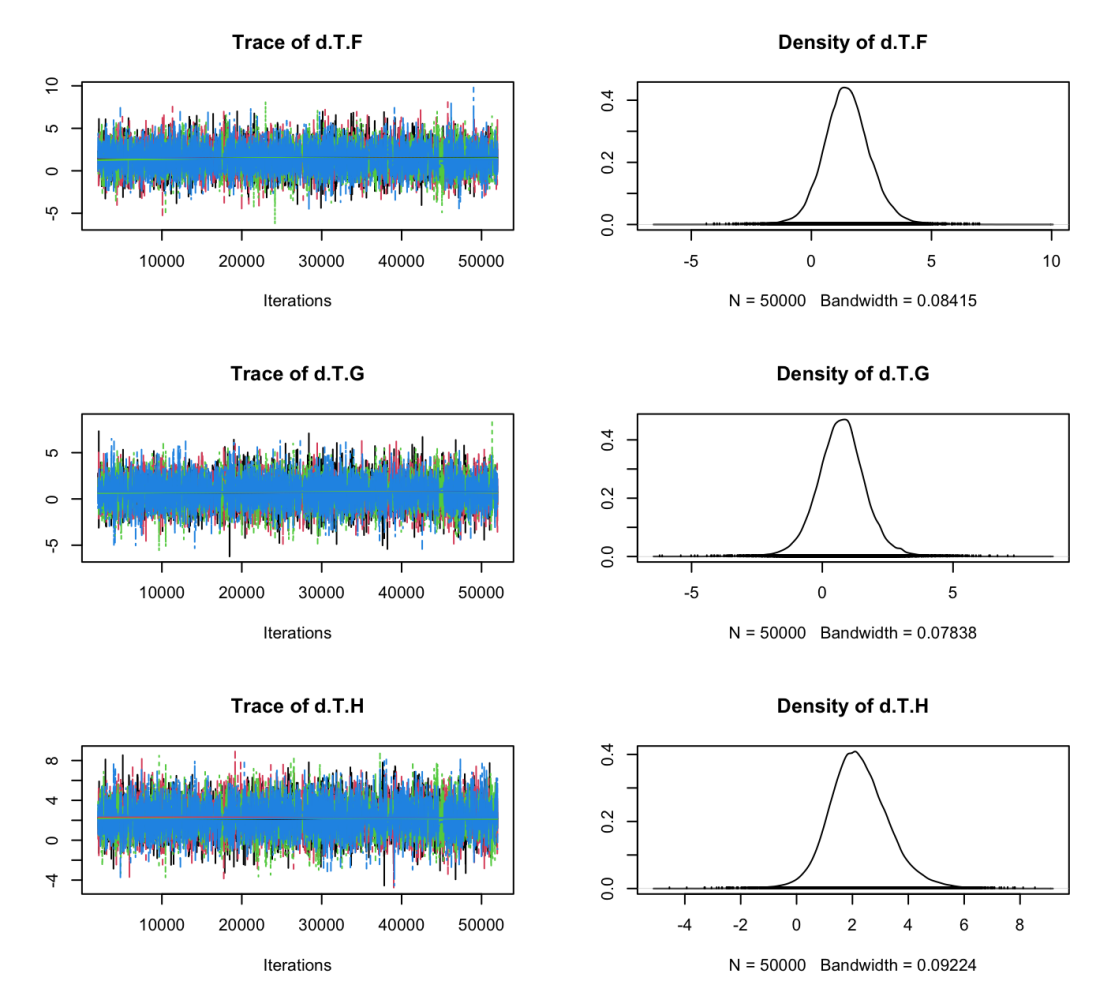


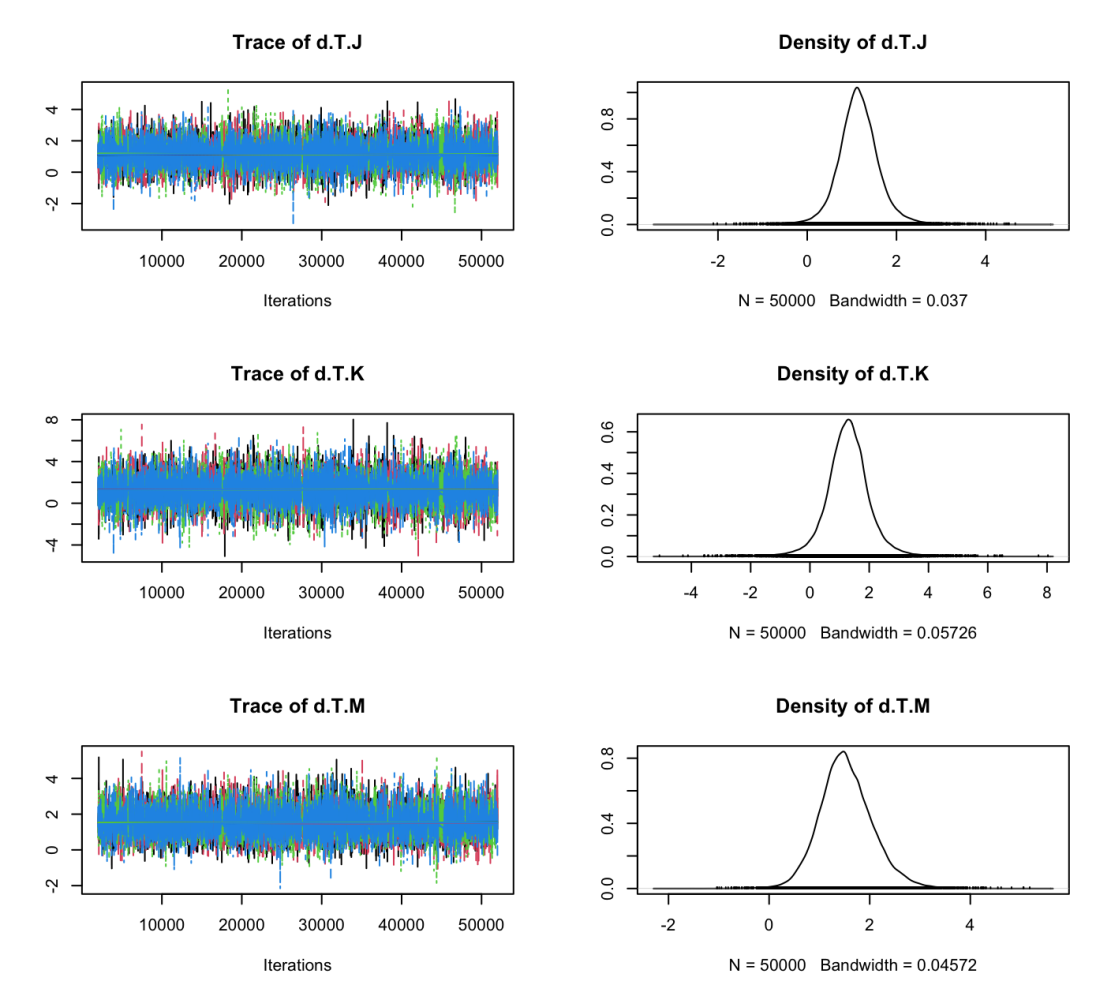


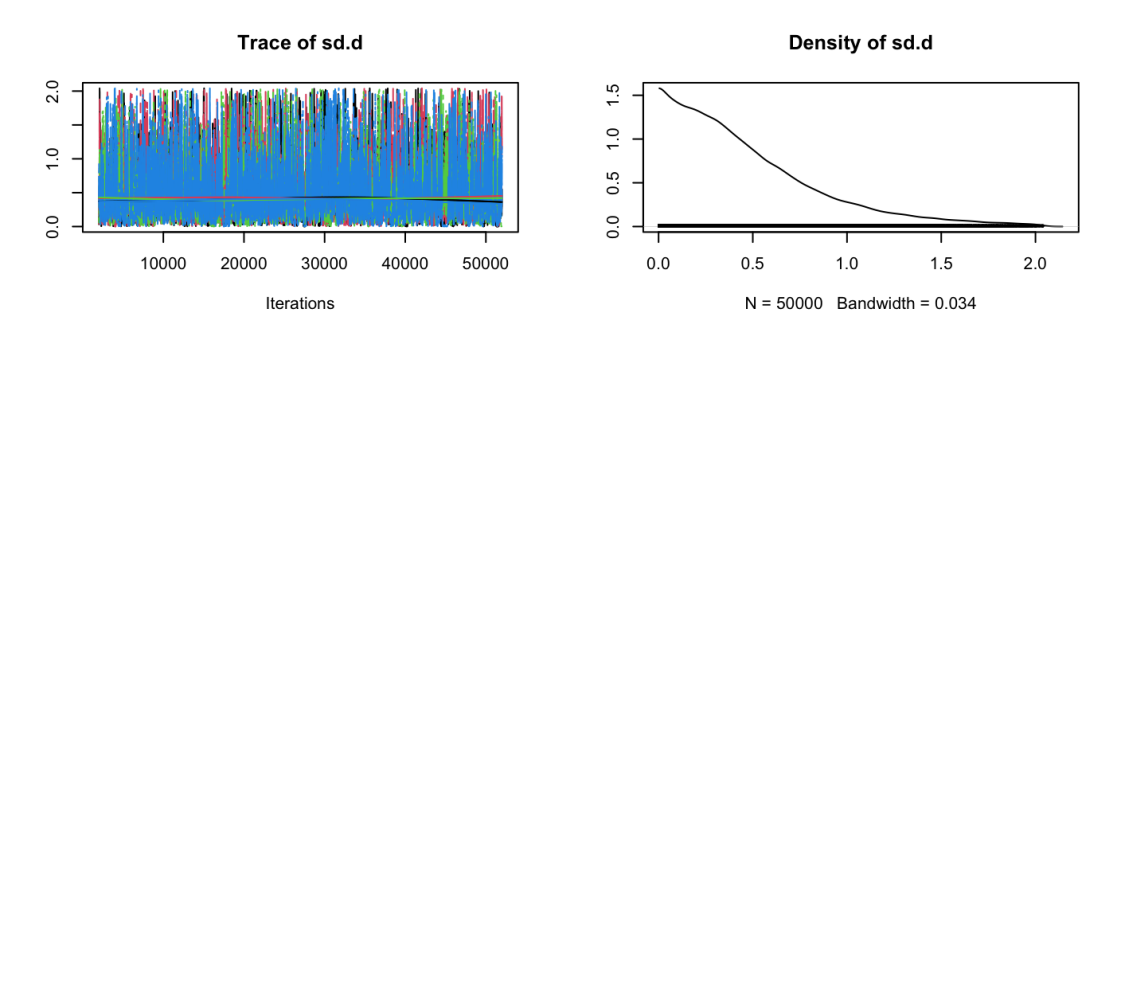


**Figure 1** **Trajectory diagram and density diagram:clinical effectiveness**


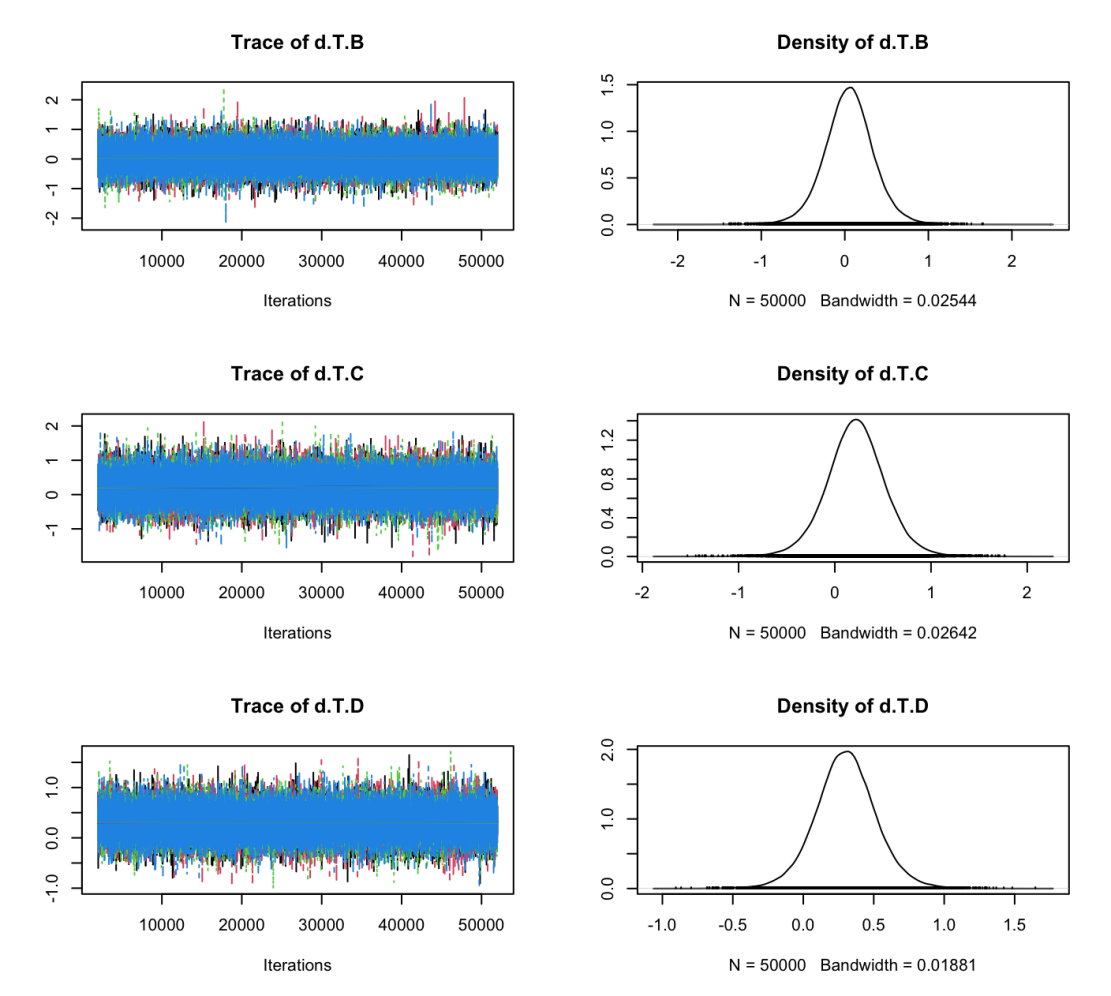


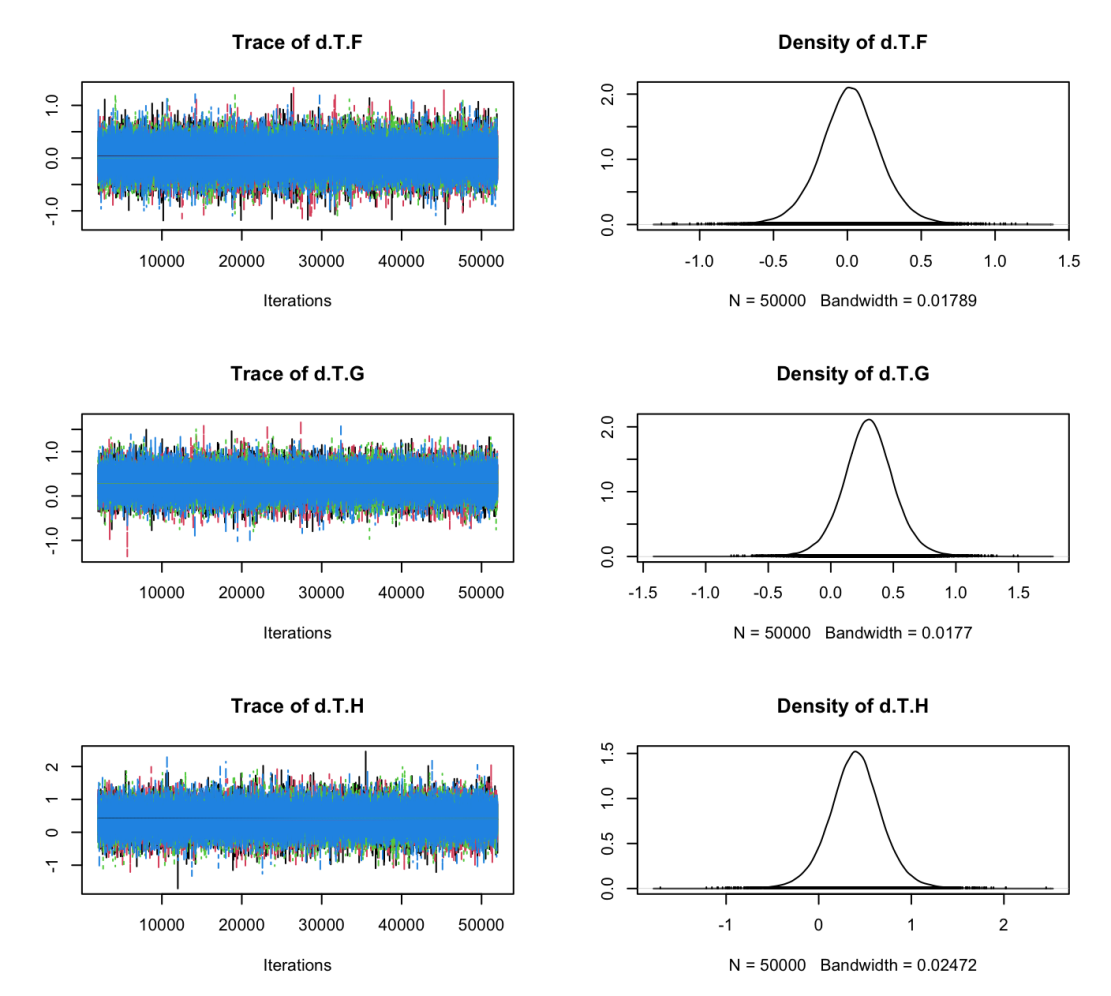


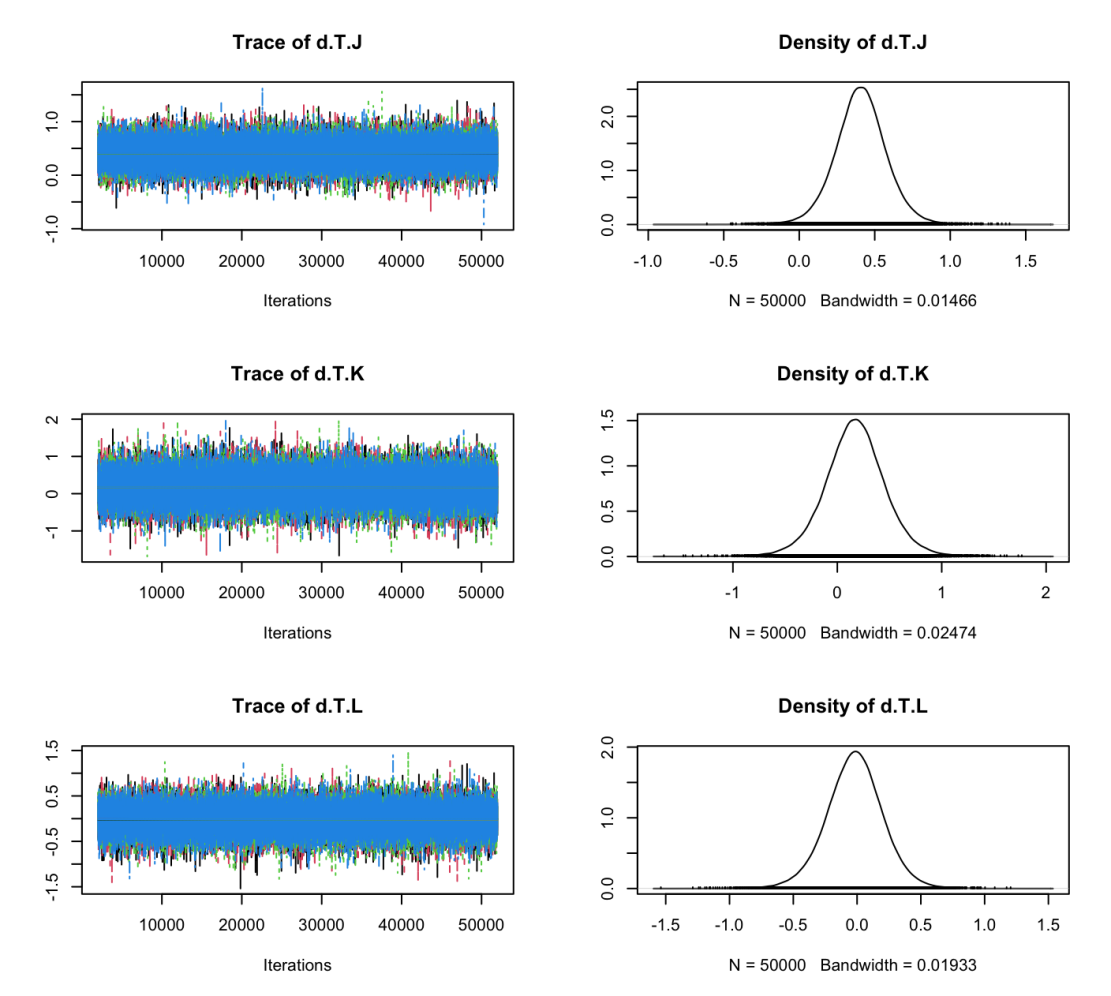


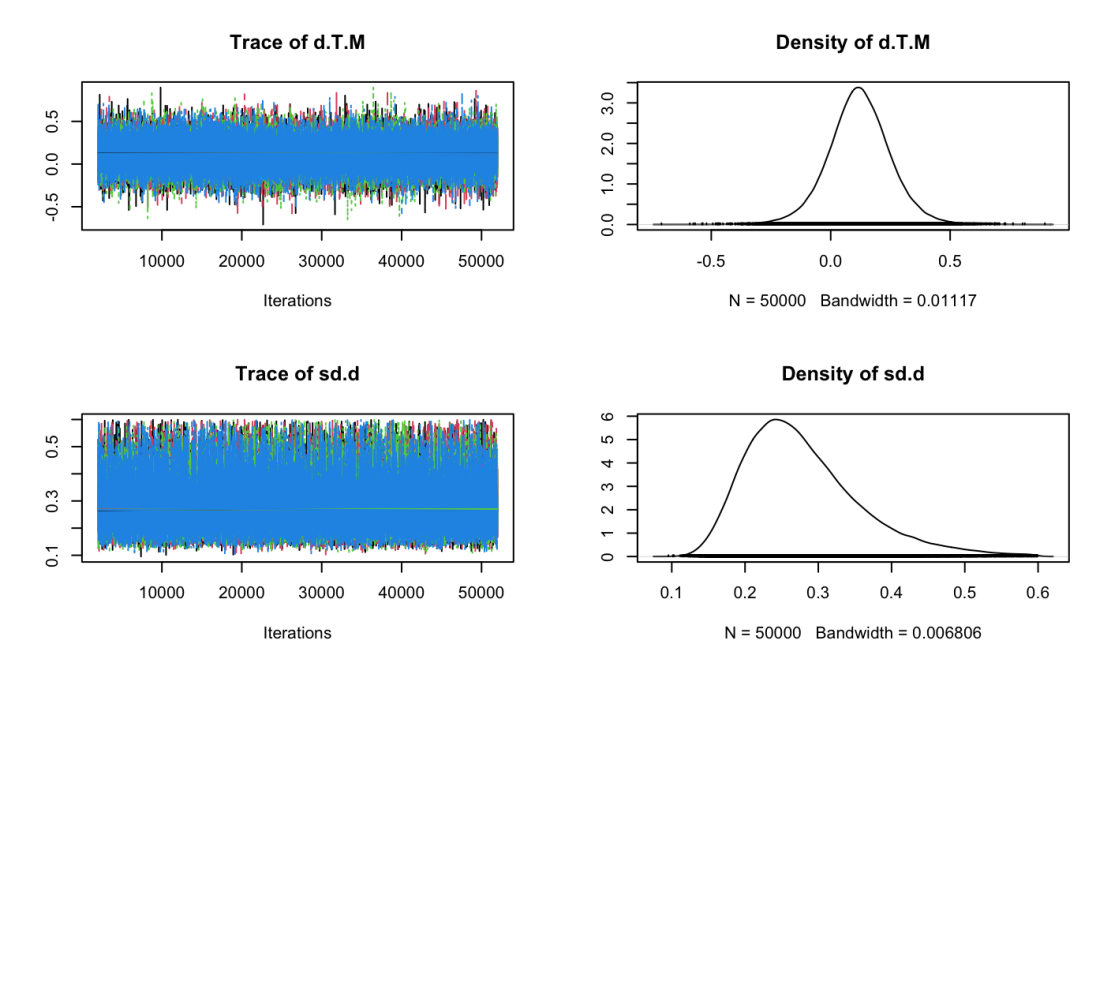


**Figure 2** **Trajectory diagram and density diagram:HDL-c**


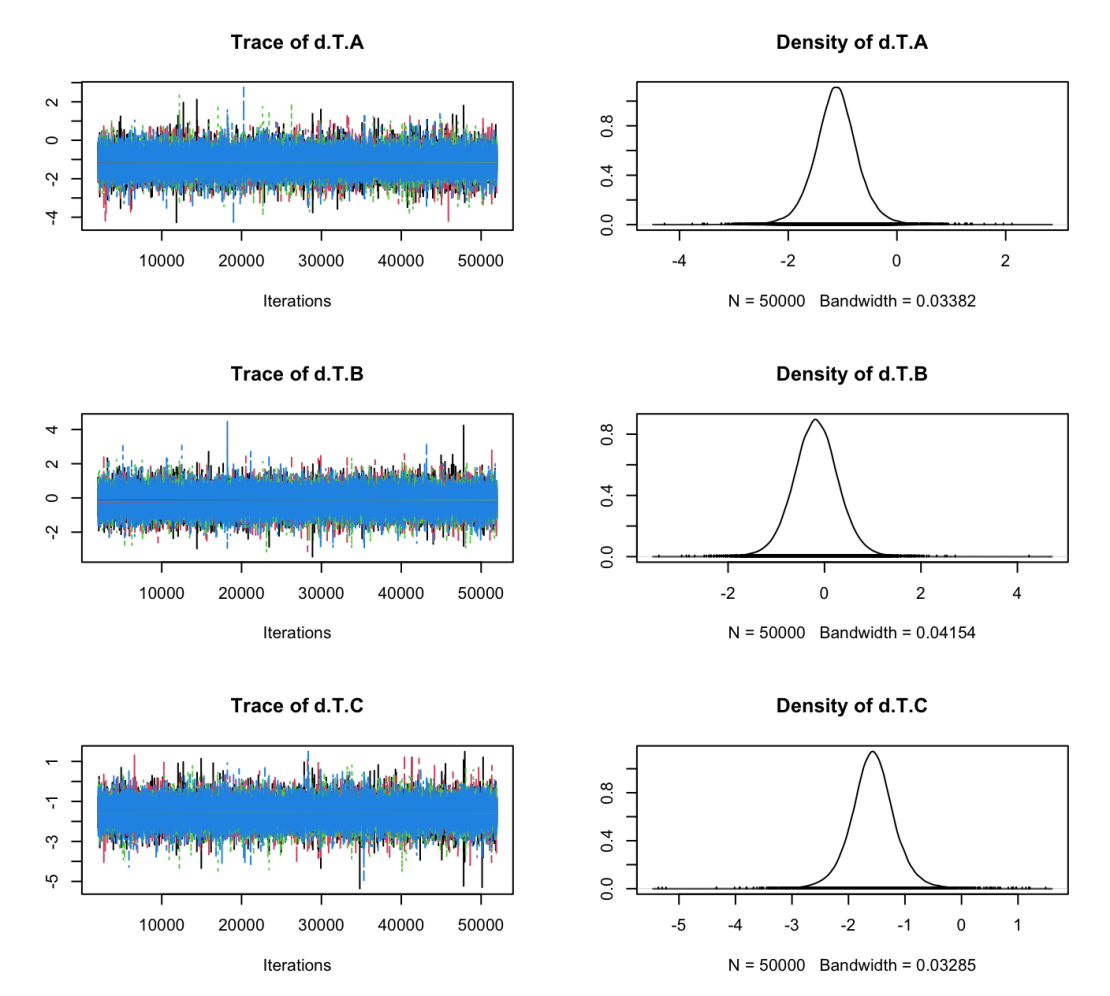


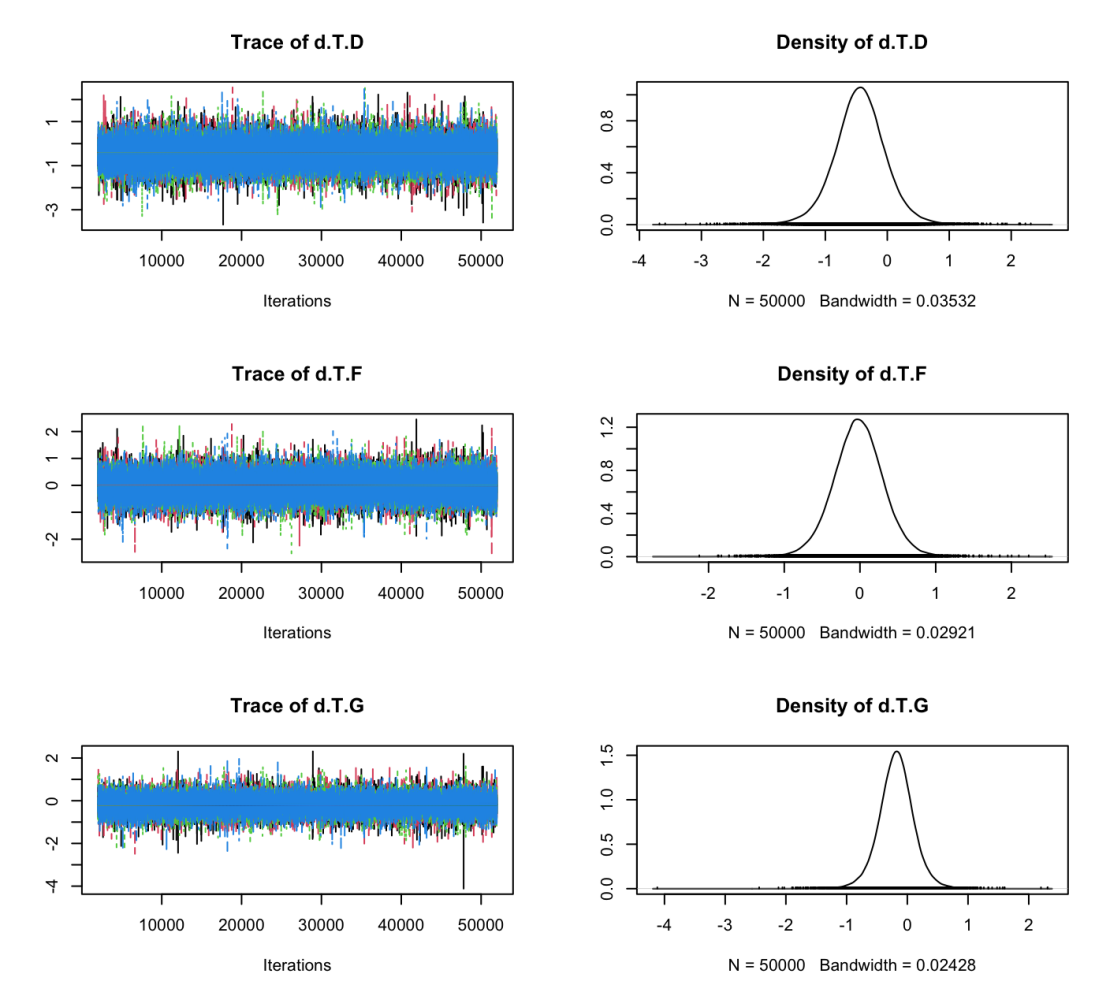


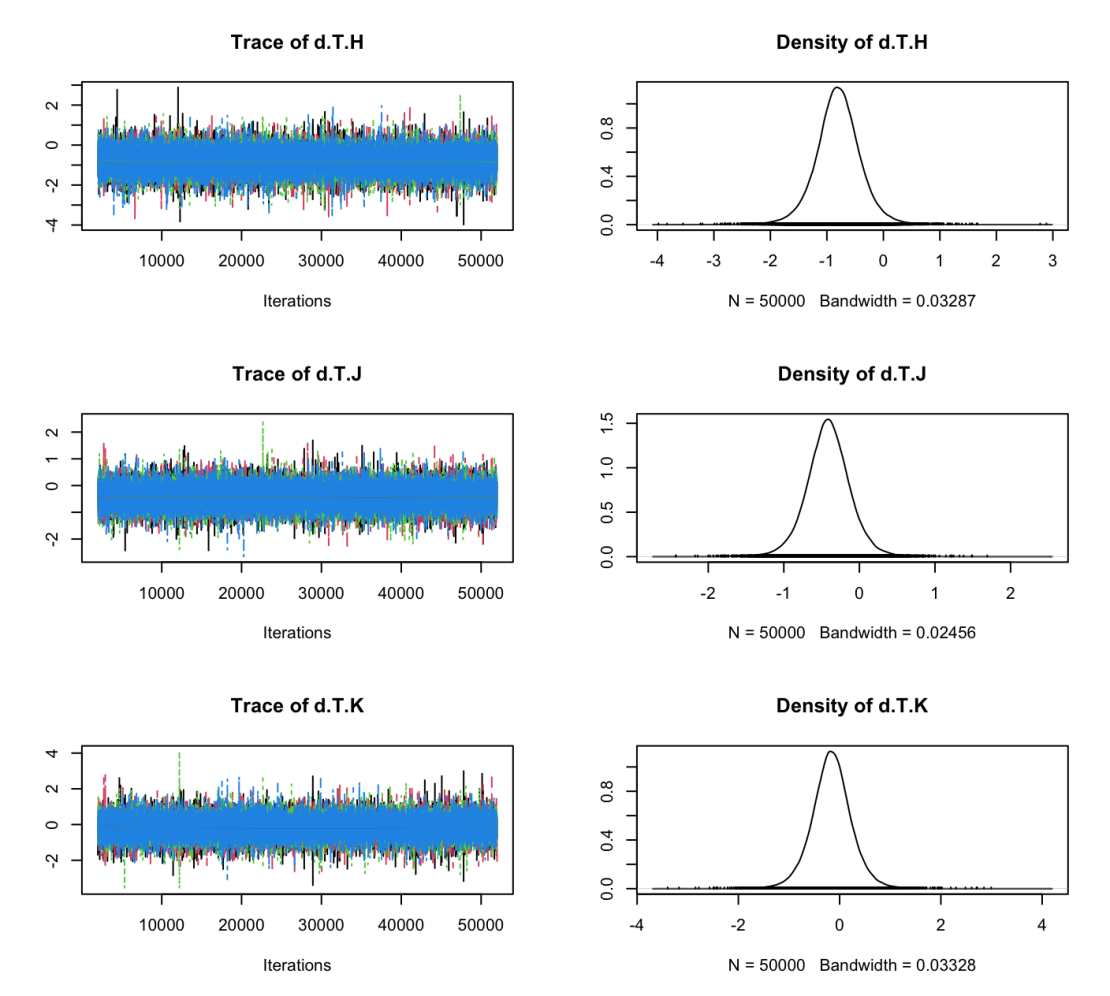


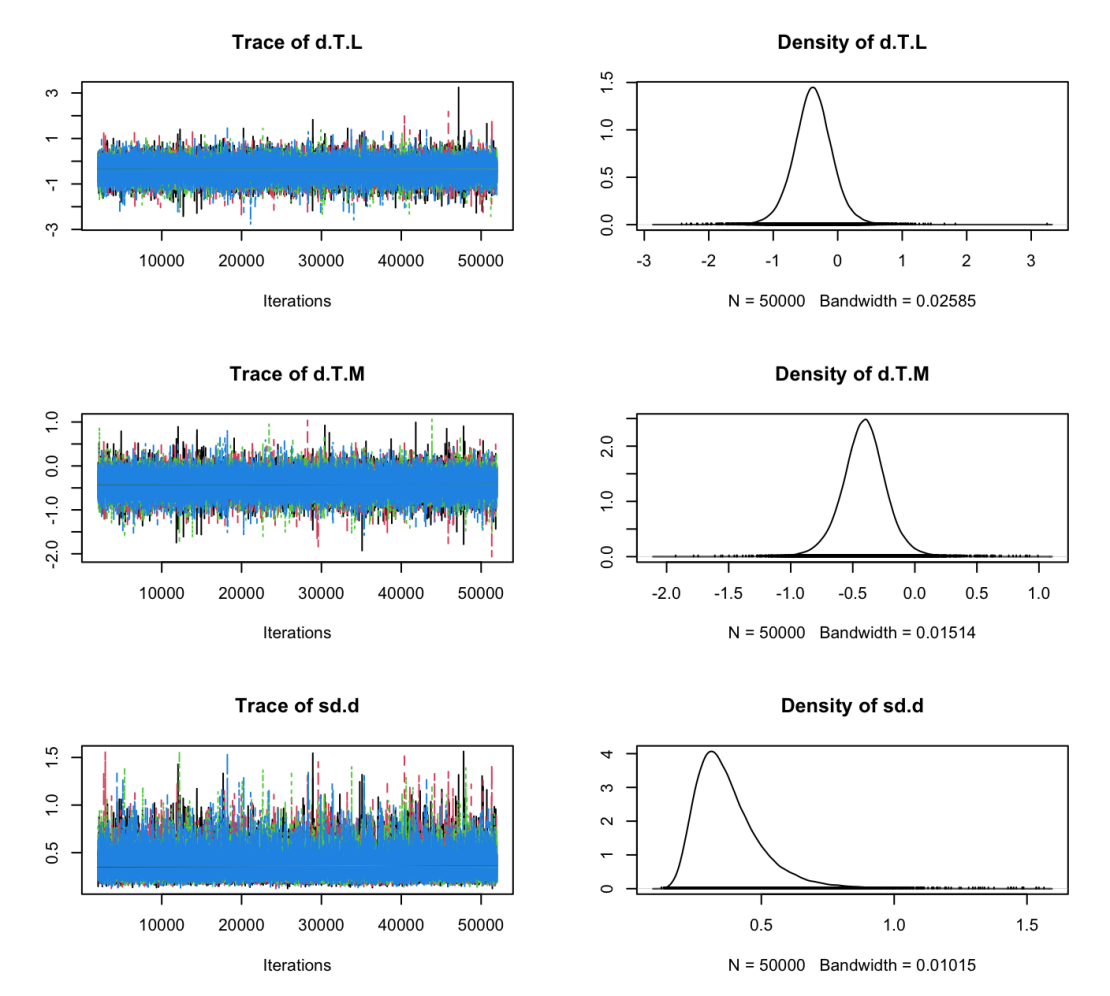


**Figure 3** **Trajectory diagram and density diagram:LDL-c**


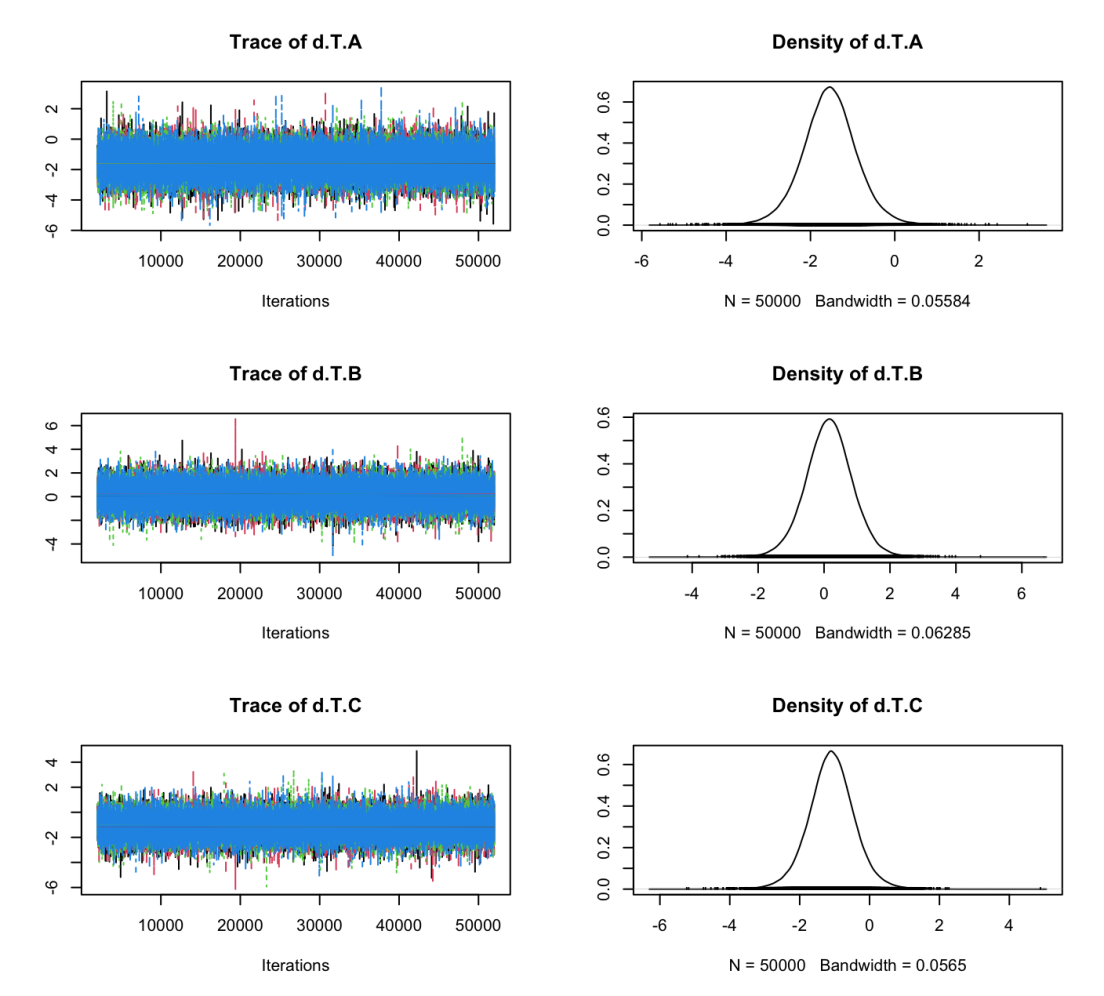


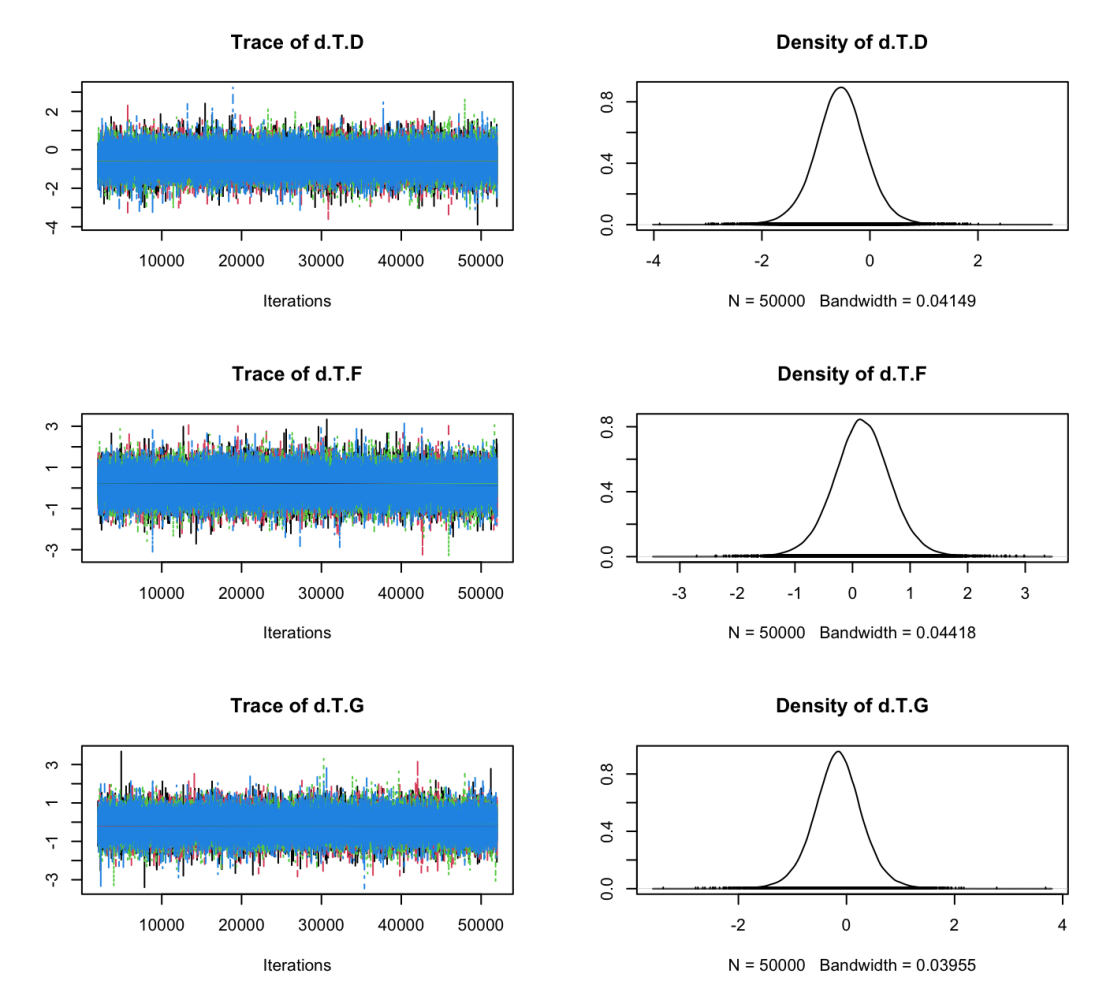


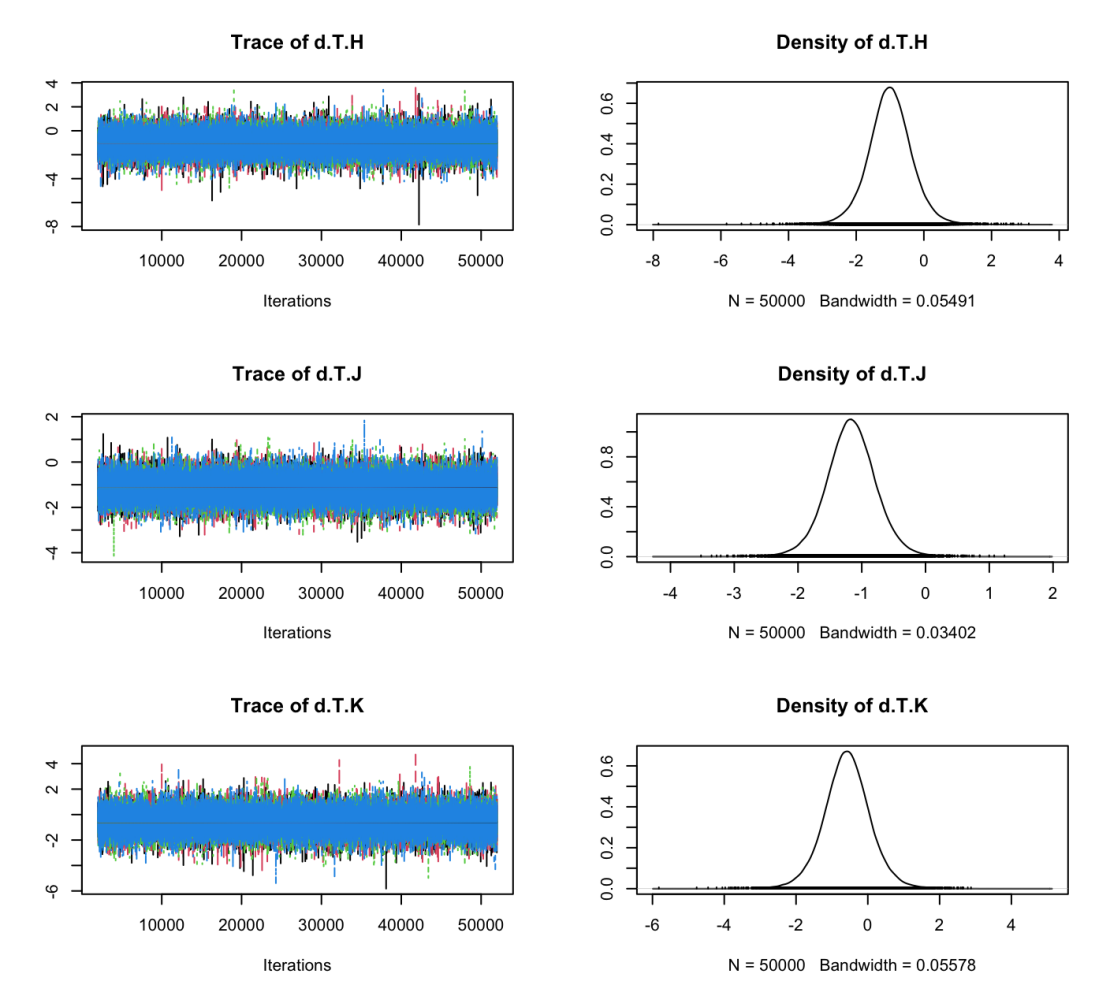


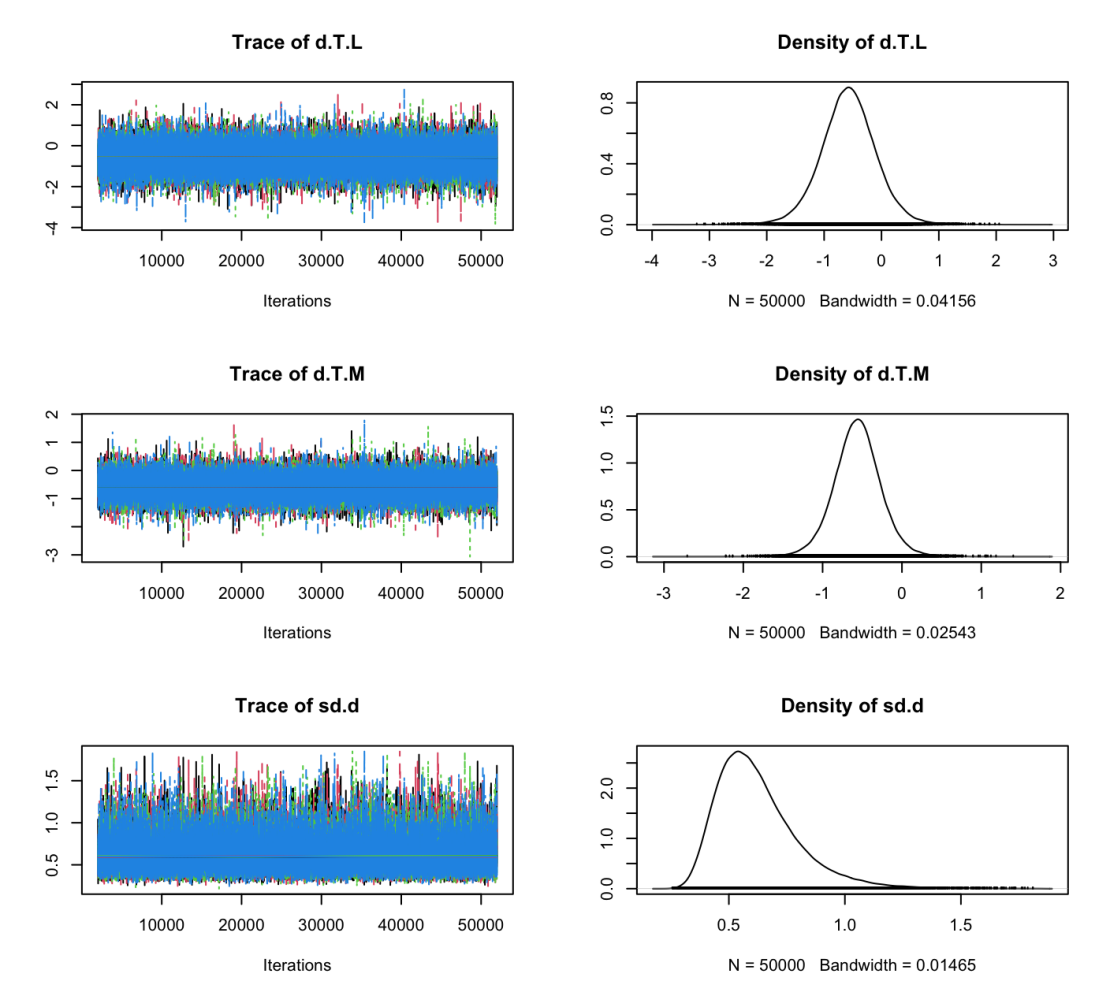


**Figure 4** **Trajectory diagram and density diagram:TC**


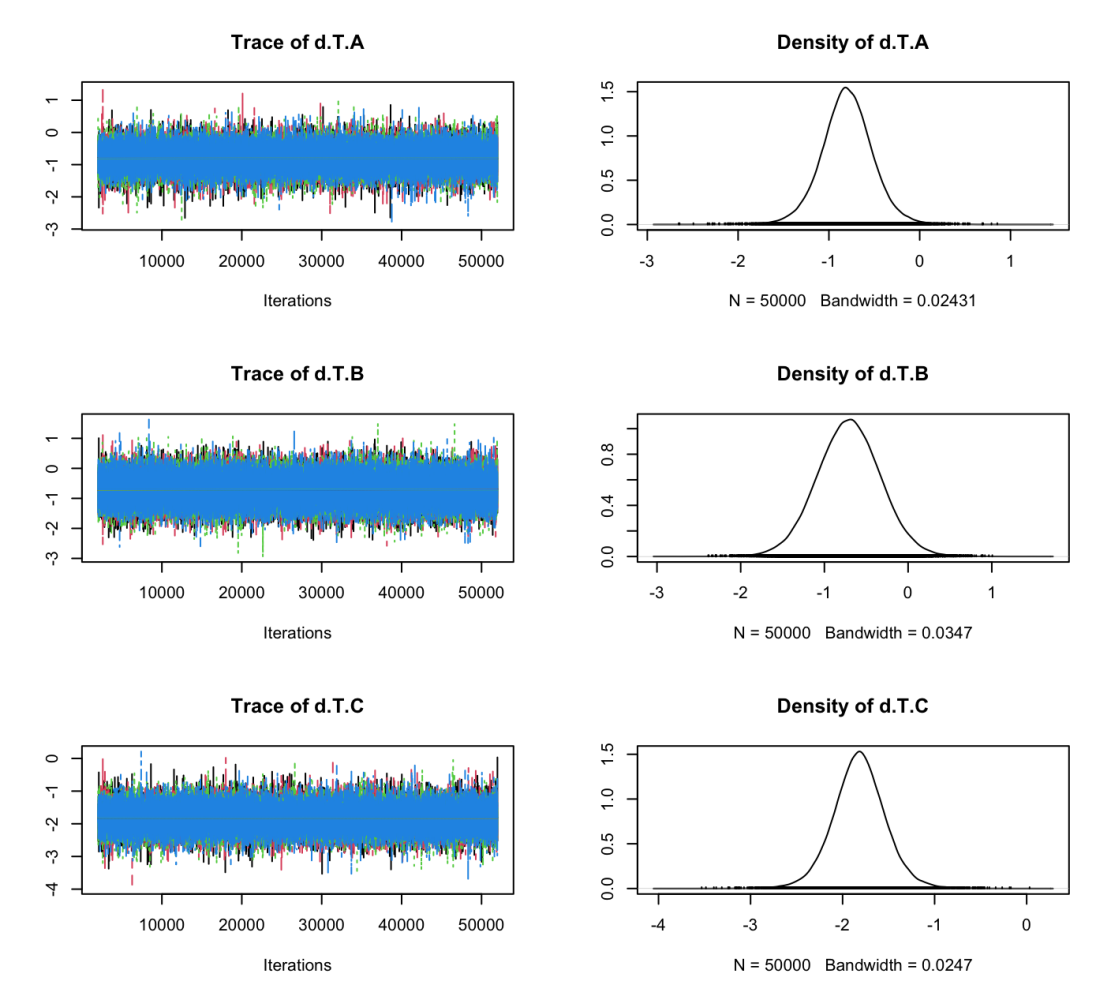


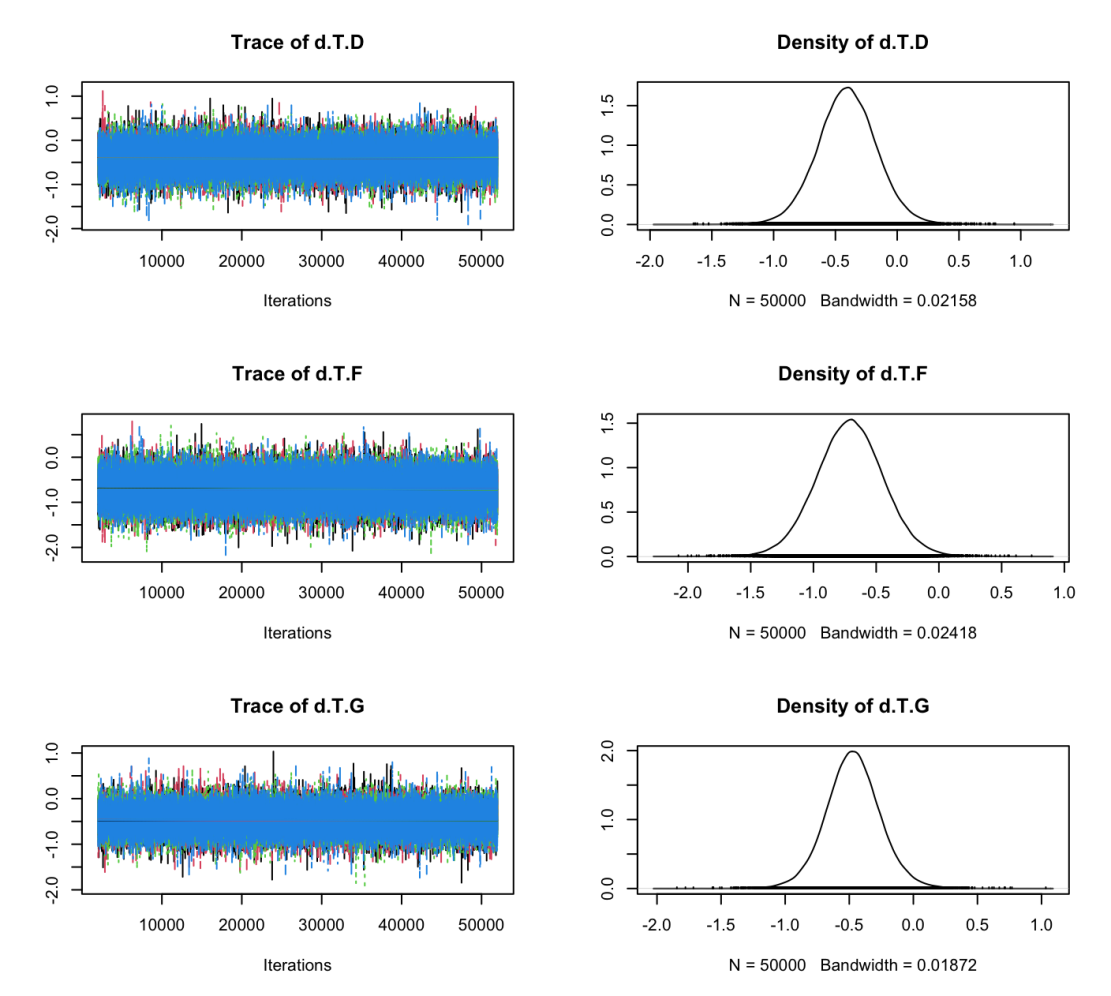


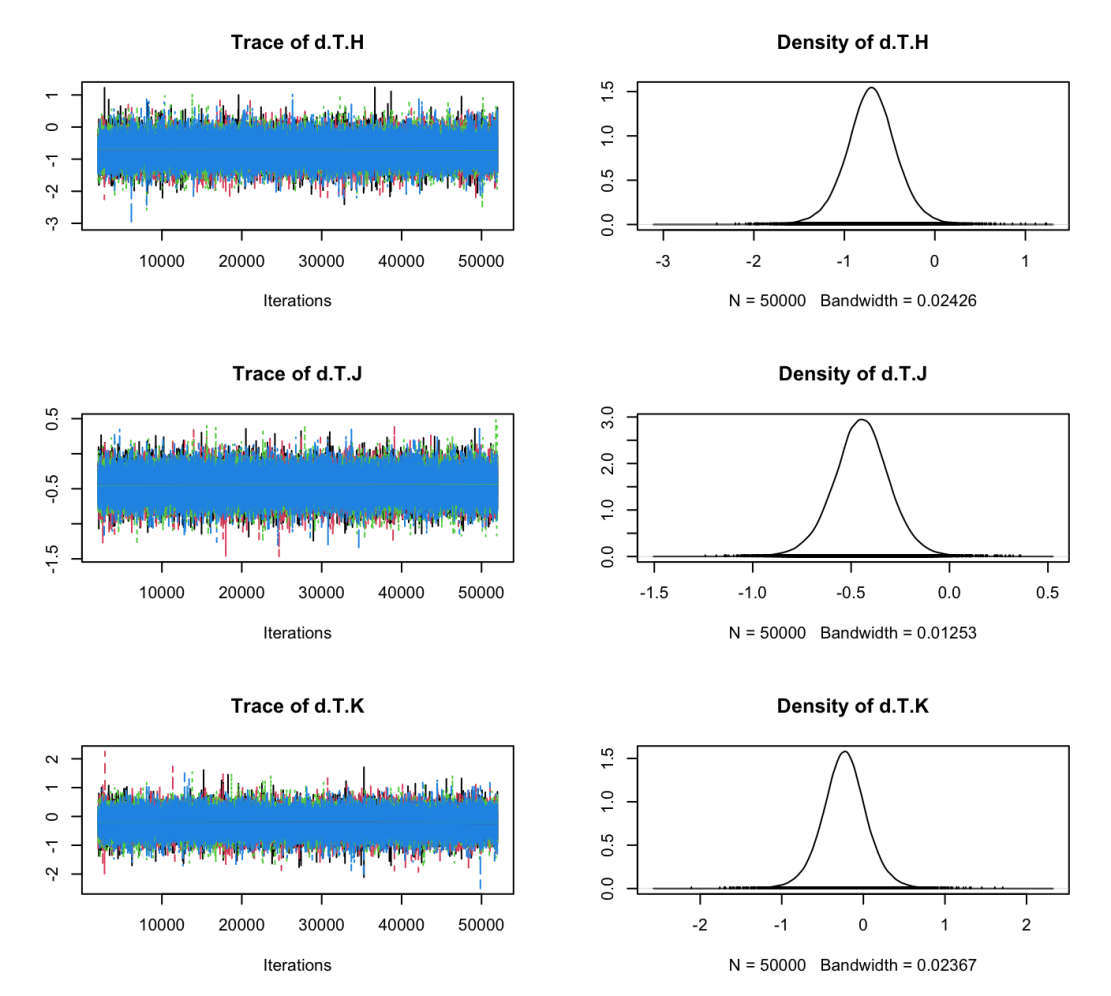


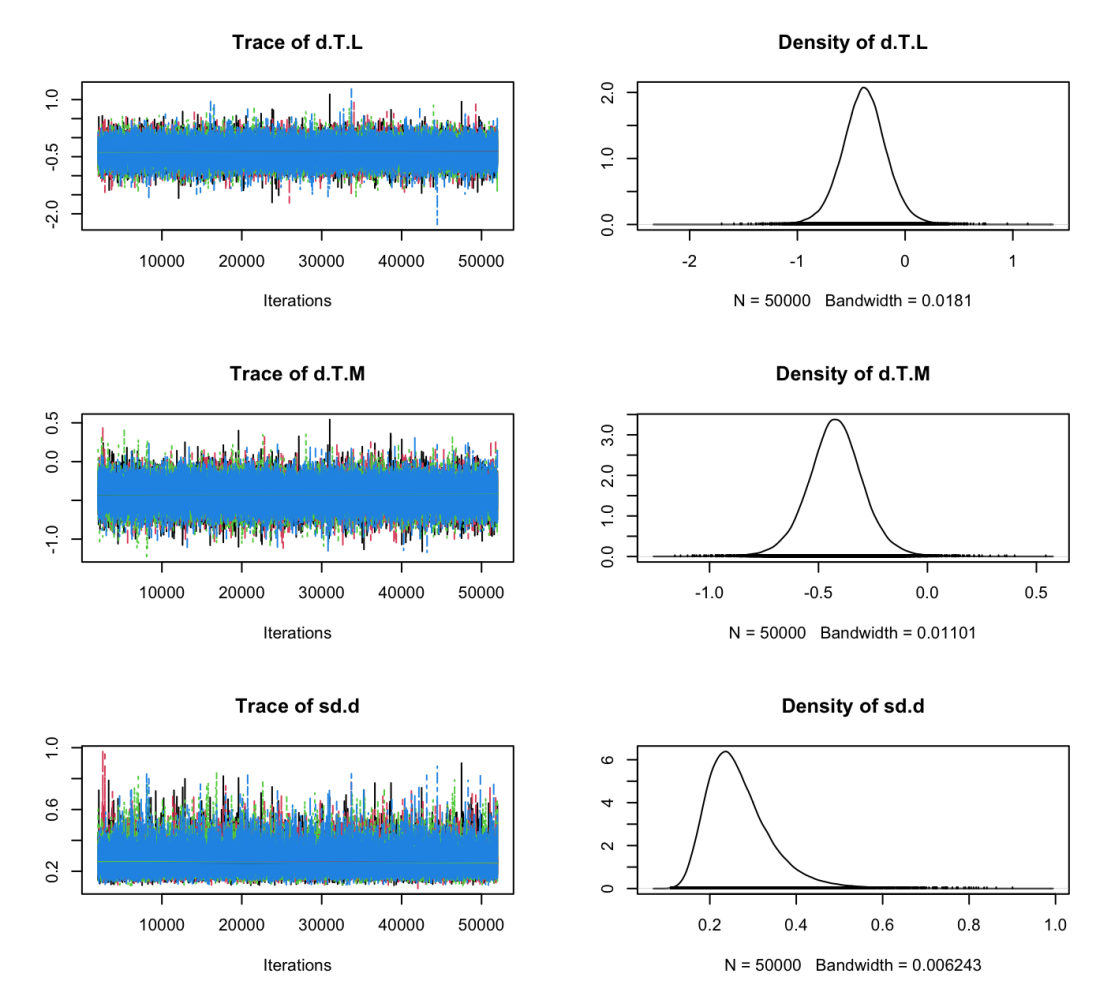


**Figure 5** **Trajectory diagram and density diagram:TG**
