# Supplementary material for: Effectiveness and safety of eleven Chinese patent medicines combined with atorvastatin in the treatment of hyperlipidemia: a network meta-analysis of randomized controlled trials
Source: Front Endocrinol (Lausanne). 2025 Mar 24;16:1523553. doi: 10.3389/fendo.2025.1523553 (PMC11973096; doi:10.3389/fendo.2025.1523553)
Supplement: Supplementary file 7 [file DataSheet7.docx]

**Supplement 7**

diagnostic graph


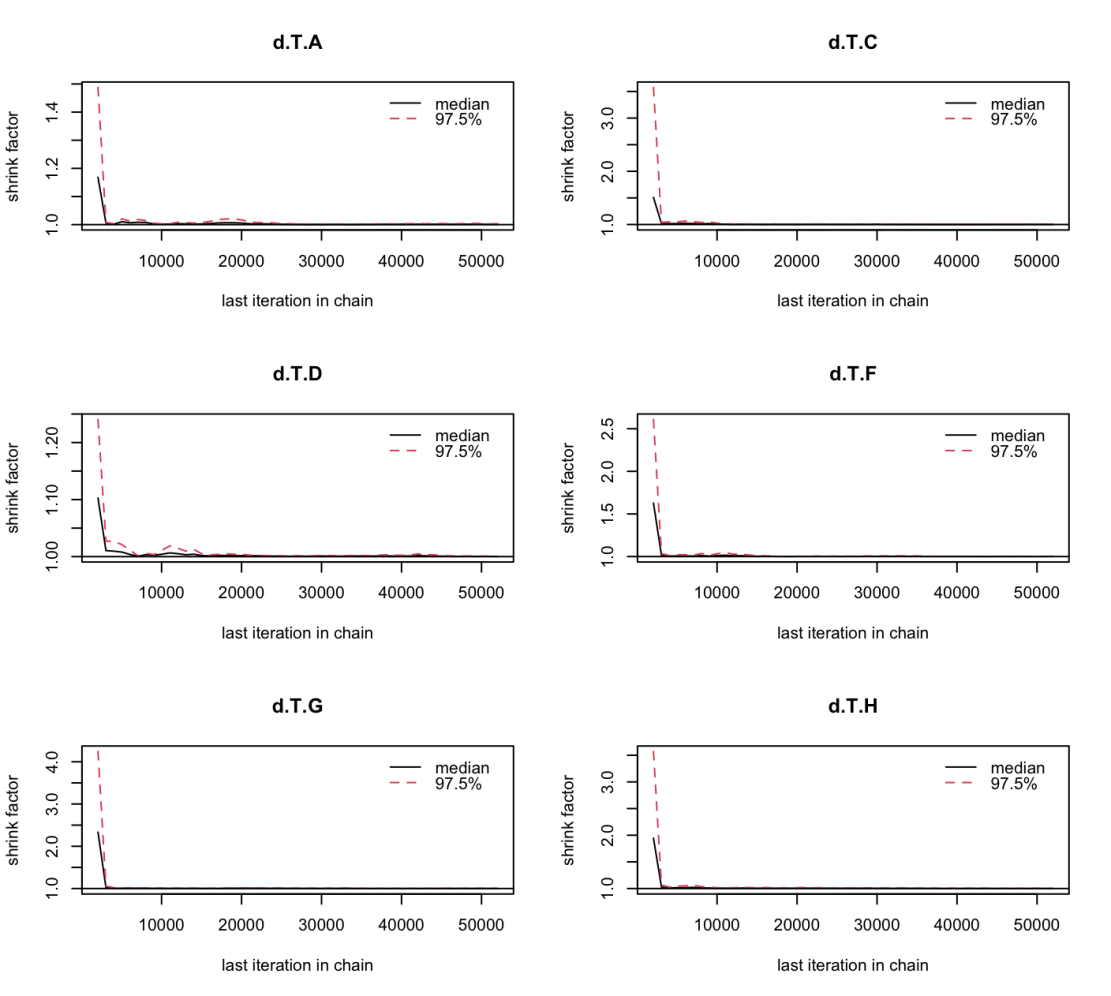


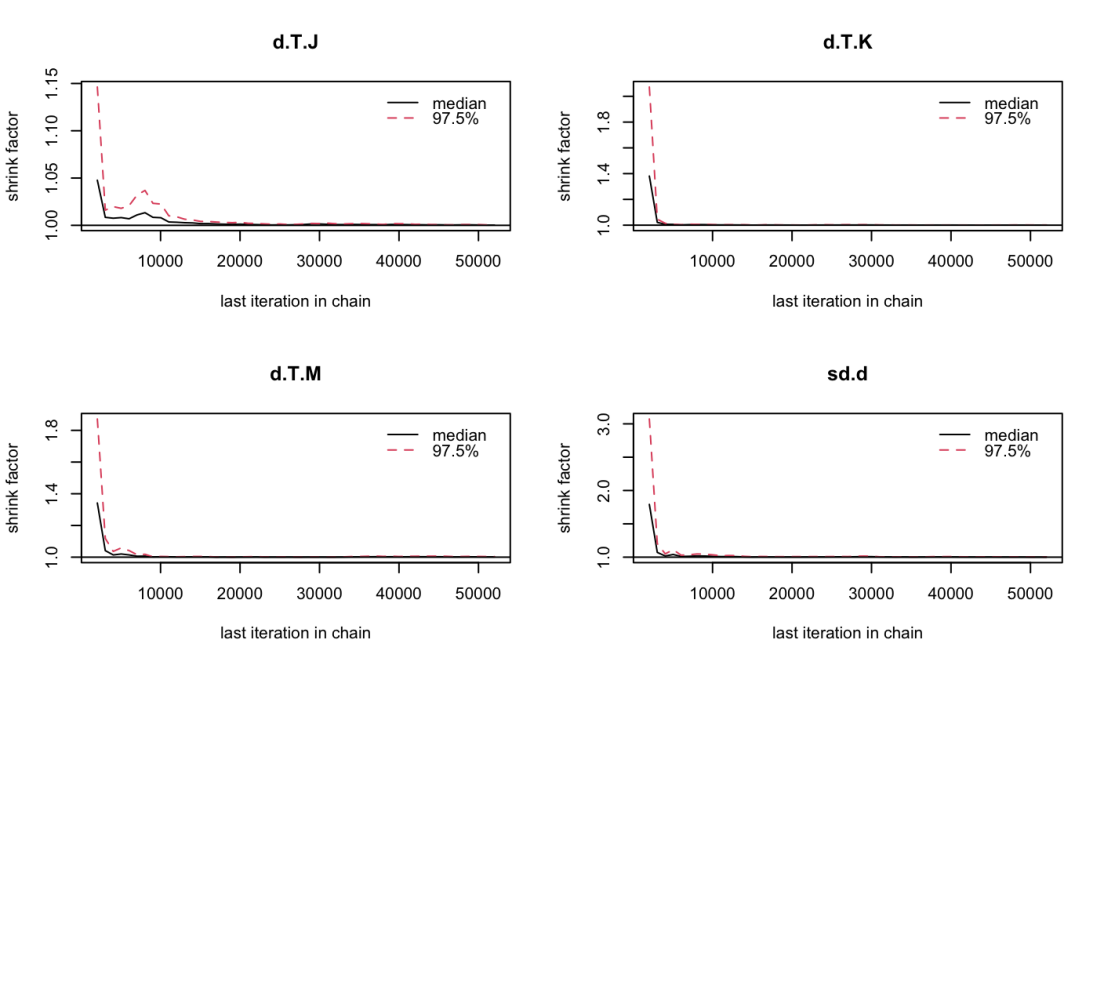


**Figure 1 diagnostic graph:clinical effectiveness**


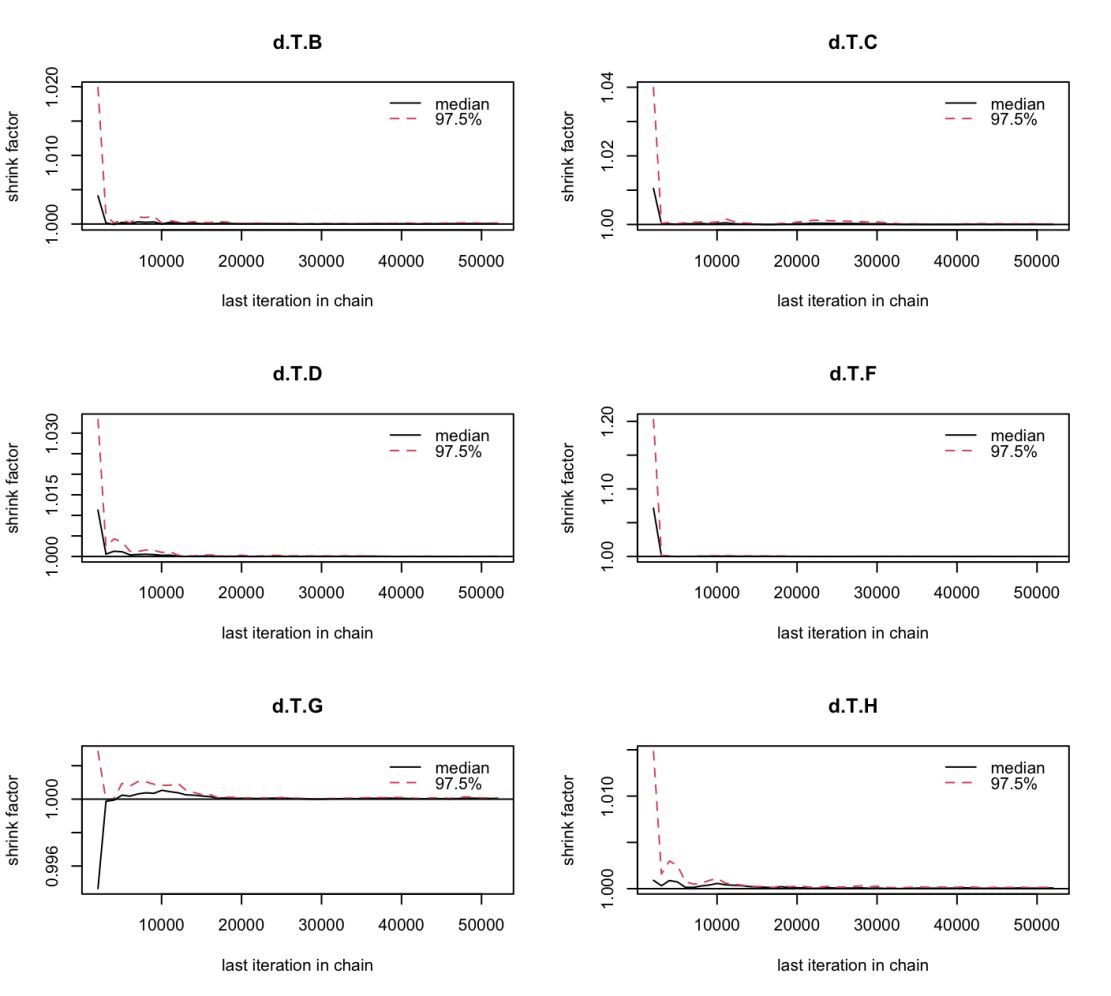


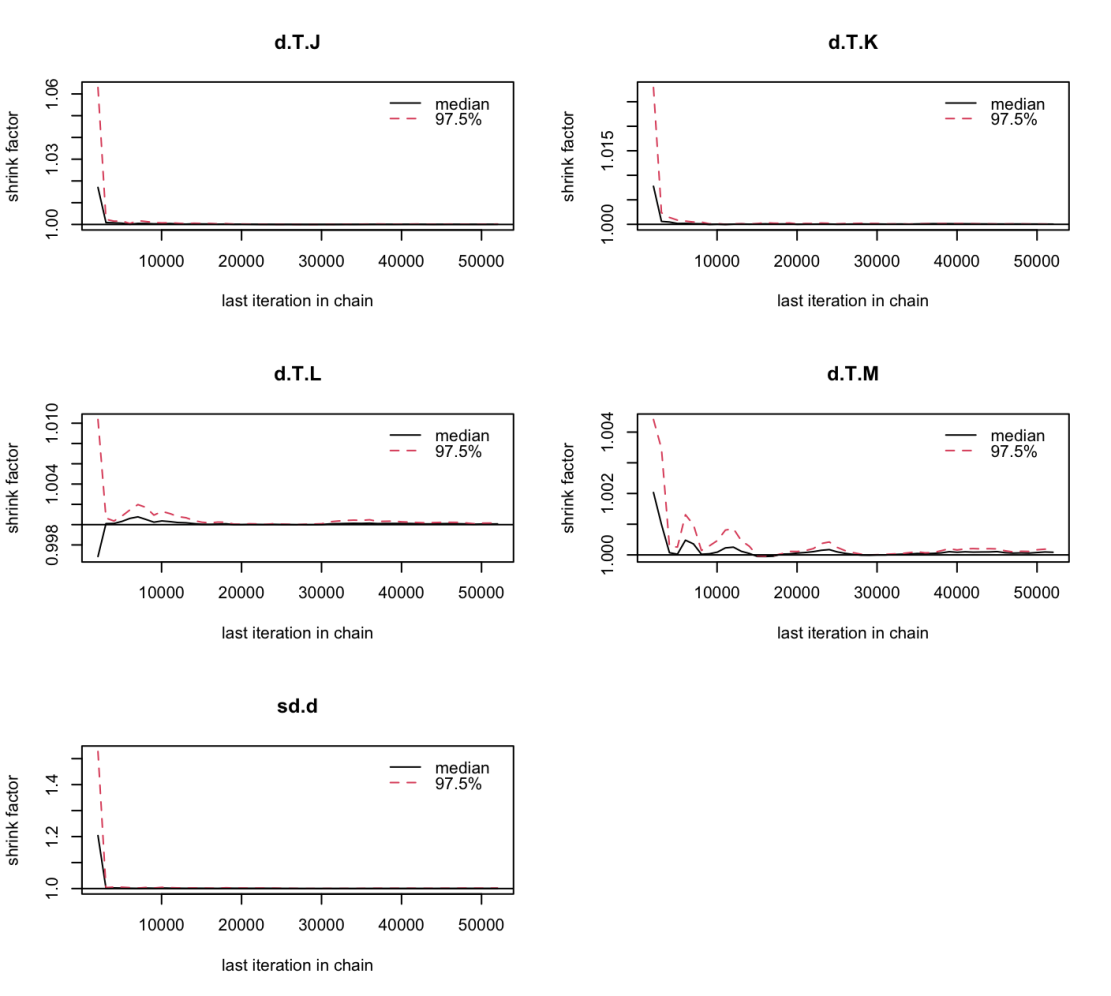


**Figure 2 diagnostic graph:HDL-c**


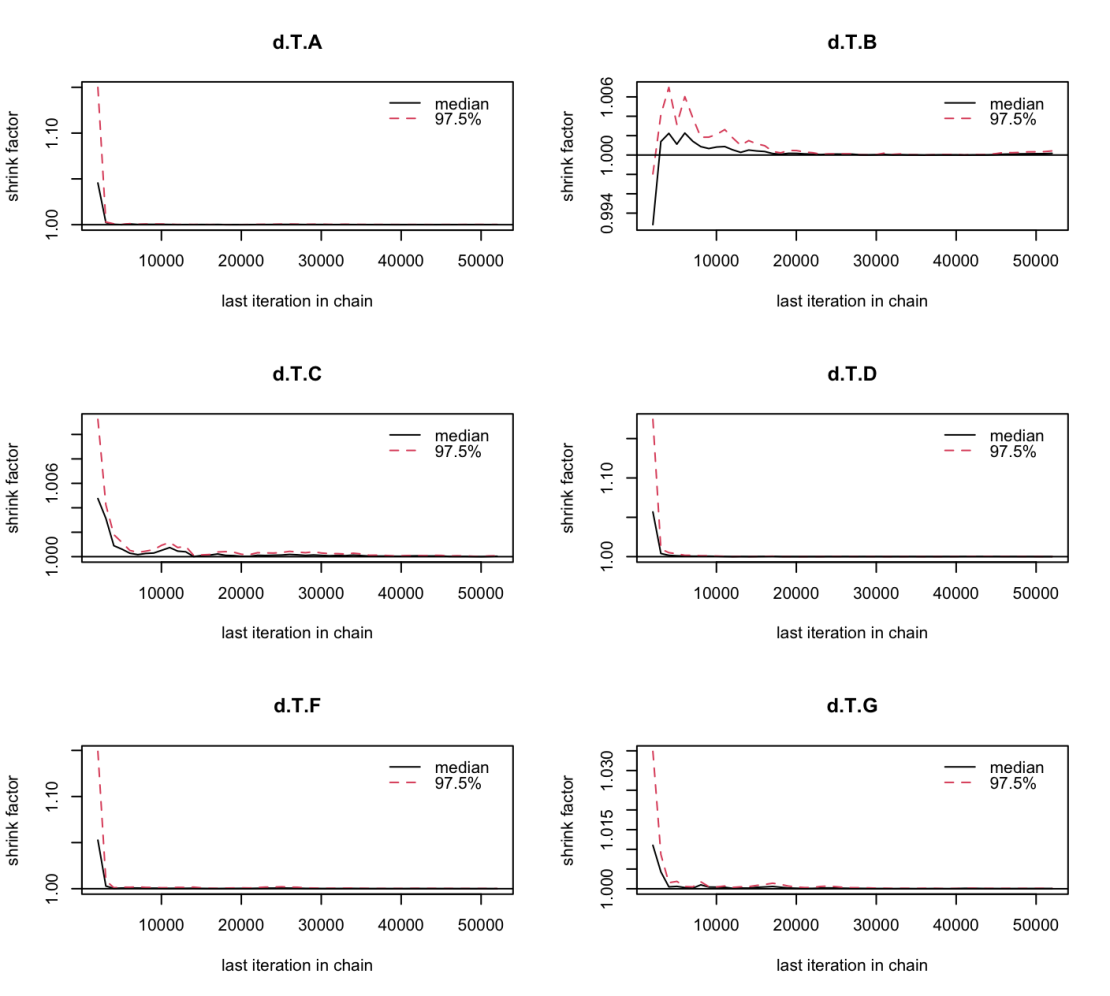


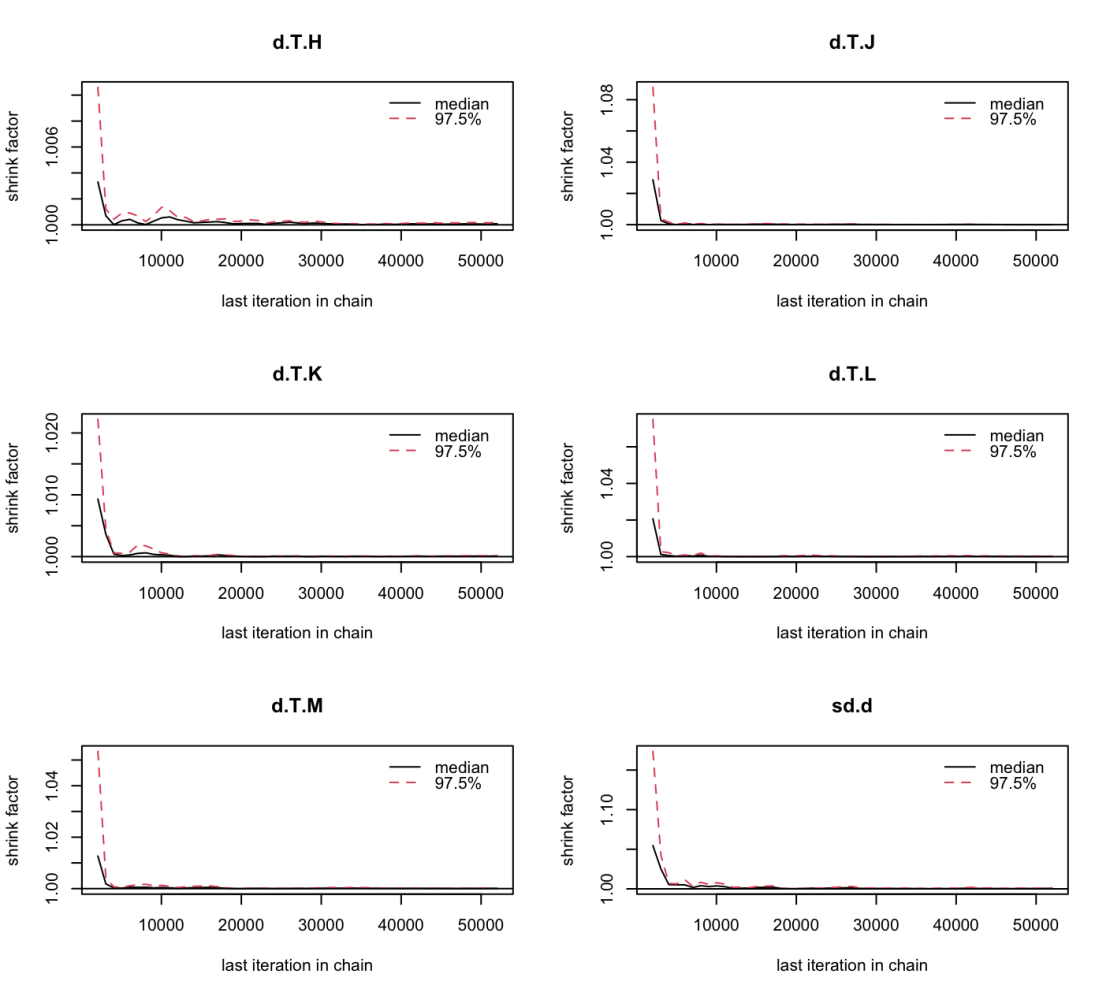


**Figure 3 diagnostic graph:LDL-c**


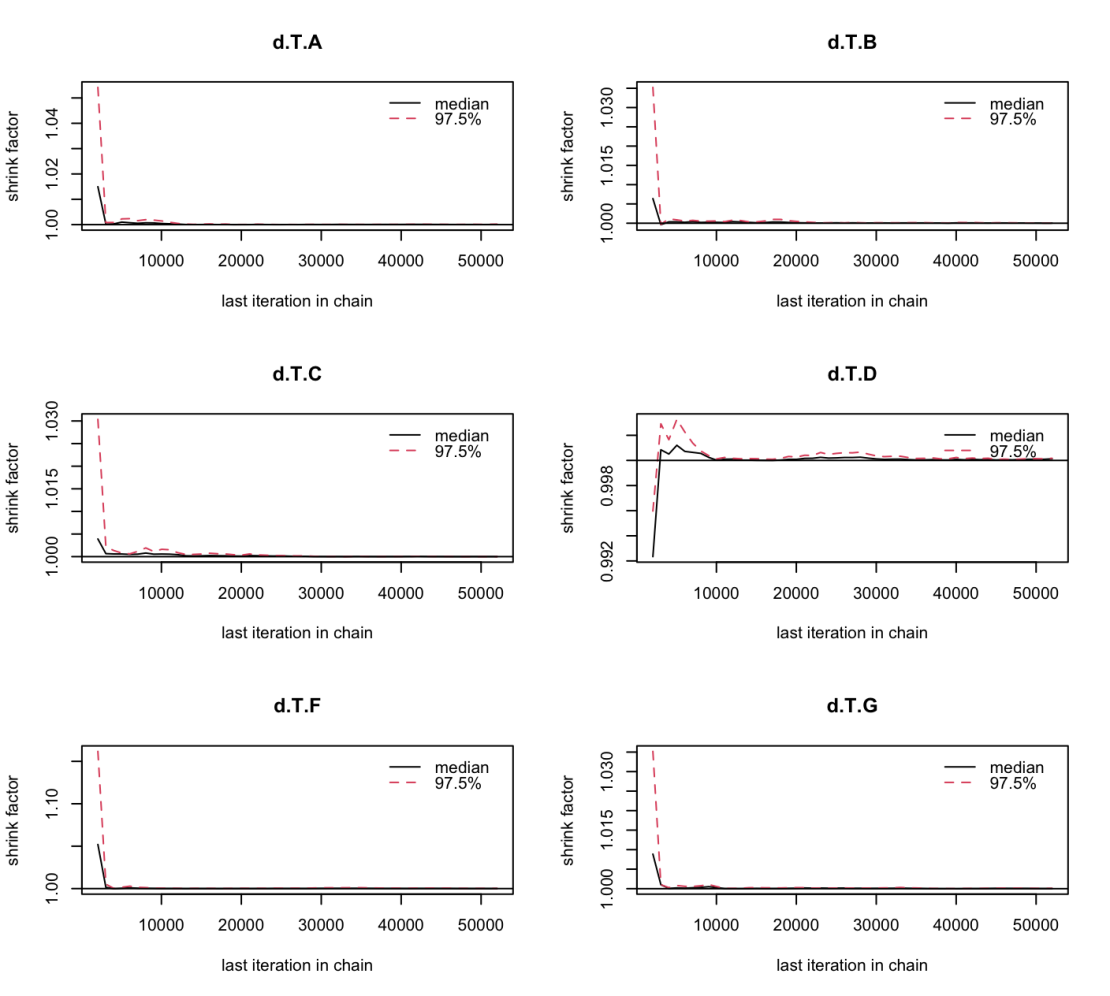


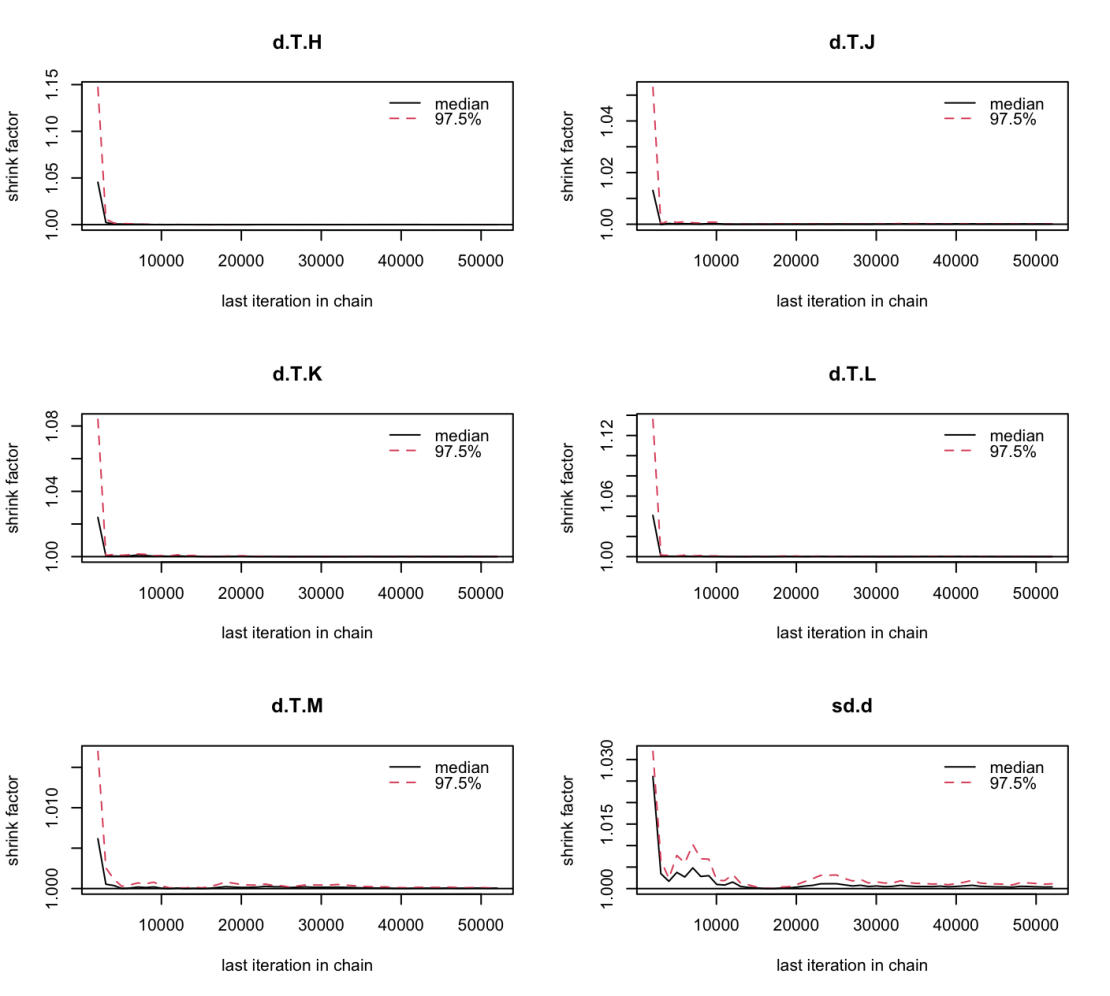


**Figure 4 diagnostic graph:TC**


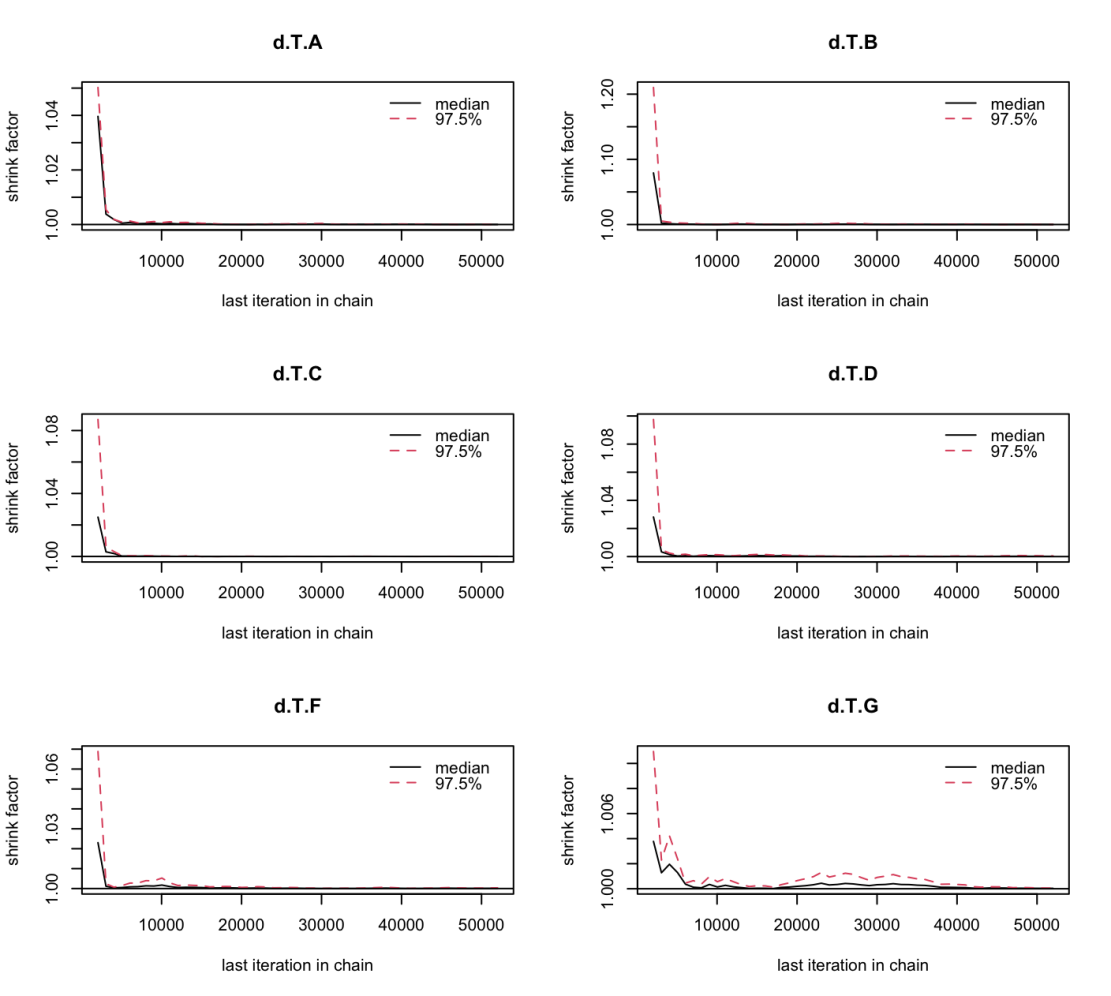


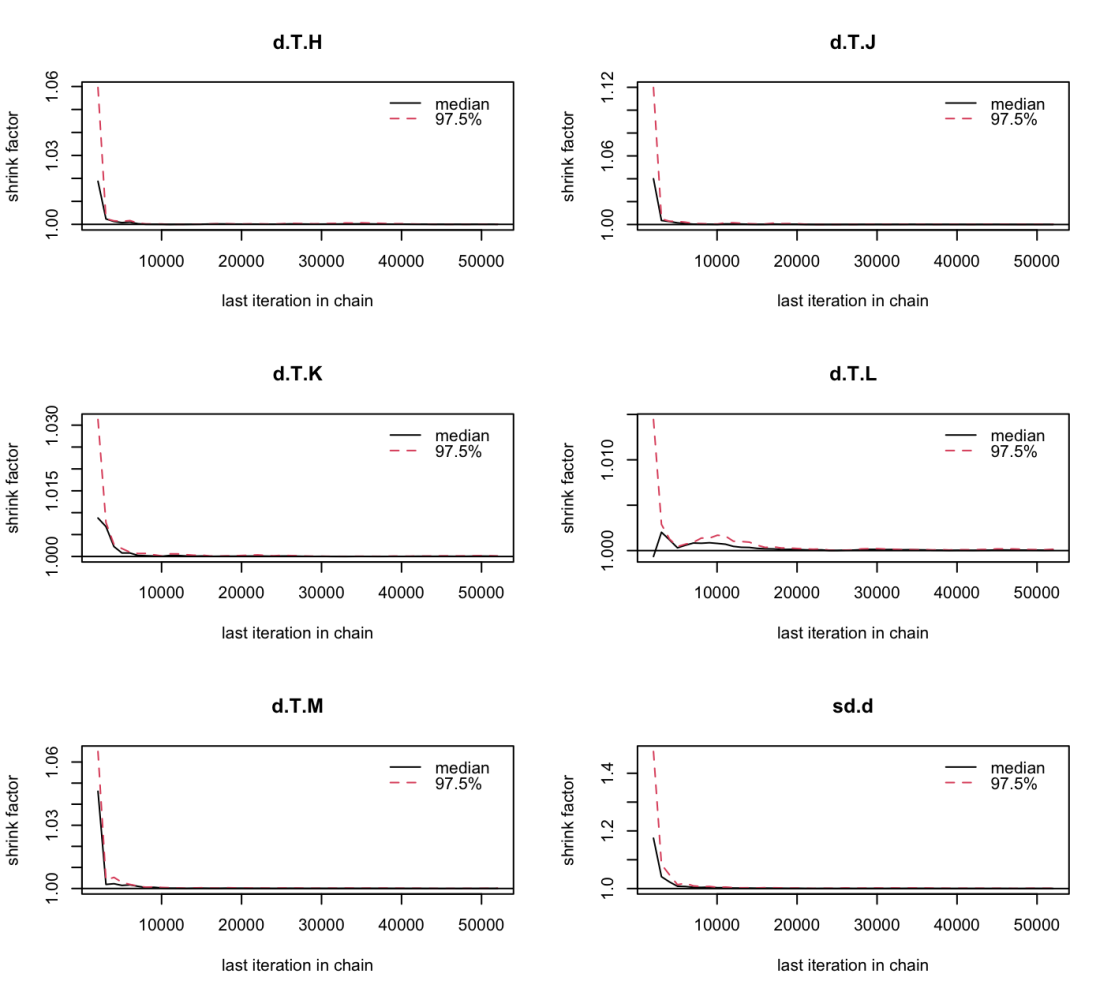


**Figure 5 diagnostic graph:TG**
