# Supplementary material for: Effectiveness and safety of eleven Chinese patent medicines combined with atorvastatin in the treatment of hyperlipidemia: a network meta-analysis of randomized controlled trials
Source: Front Endocrinol (Lausanne). 2025 Mar 24;16:1523553. doi: 10.3389/fendo.2025.1523553 (PMC11973096; doi:10.3389/fendo.2025.1523553)
Supplement: Supplementary file 8 [file DataSheet8.docx]

**Supplement 8**

Clinical data of 138 patients with mixed hyperlipidemia were analyzed

Table1 Clinical data of 138 patients with mixed hyperlipidemia were analyzed

| **Item** | **Combination group （69 cases）** | **Single statin group （69 cases）** | **P** |
| --- | --- | --- | --- |
| age（year，$\overline{\text{x}}\pm\text{s}$） | 55.16±9.77 | 53.72±10.62 | 0.4069 |
| sex[cases（%）] |  |  |  |
| males | 38（55.07） | 35（50.72） | 0.6089 |
| female | 31（44.93） | 34（49.28） |  |
| body mass index（$\overline{\text{x}}\pm\text{s}$） | 26.14±3.22 | 26.88±3.25 | 0.1807 |
| smoking history[cases（%）] |  |  |  |
| NO | 44（63.77） | 45（65.22） | 0.8588 |
| Yes | 25（36.23） | 24（34.78） |  |
| History of hypertension[cases（%）] |  |  |  |
| NO | 44（63.77） | 38（55.07） | 0.2983 |
| Yes | 25（36.23） | 31（44.93） |  |
| history of diabetes[cases（%）] |  |  |  |
| NO | 58（84.06） | 61（88.41） | 0.4586 |
| Yes | 11（15.94） | 8（11.59） |  |
| History of hyperlipidemia（year，$\overline{\text{x}}\pm\text{s}$） | 3.97±5.05 | 3.34±4.38 | 0.3478 |
| blood fat（mmol/L,$\overline{\text{x}}\pm\text{s}$） |  |  |  |
| TG | 3.39±0.97 | 3.28±0.84 | 0.4546 |
| TC | 6.33±0.90 | 6.15±0.87 | 0.2463 |
| LDL-C | 4.19±0.73 | 4.11±0.65 | 0.4974 |
| HDL-C | 1.13±0.28 | 1.17±0.30 | 0.3758 |

Table2 Comparison of blood lipid changes at different time points before and after treatment in the two groups of patients（mmol/L,$\overline{\text{x}}\pm\text{s}$）

| **Group** | **Cases** | **Time** | **TG** | **TC** | **LDL-C** | **HDL-C** |
| --- | --- | --- | --- | --- | --- | --- |
| Combination group | 60 | before treatment | 3.46±1.00 | 6.34±0.94 | 4.23±0.72 | 1.13±0.29 |
|  |  | Treatment for 4 weeks | 2.50±1.42 | 4.76±1.20 | 2.83±0.79 | 1.19±0.29 |
|  |  | Treatment for 8 weeks | 2.20±0.84 | 4.62±0.98 | 2.84±0.88 | 1.19±0.22 |
| Single statin group | 62 | before treatment | 3.25±0.79 | 6.14±0.88 | 4.12±0.62 | 1.18±0.30 |
|  |  | Treatment for 4 weeks | 2.34±1.38 | 4.55±0.97 | 2.73±0.82 | 1.16±0.30 |
|  |  | Treatment for 8 weeks | 2.52±1.78 | 4.53±0.76 | 2.71±0.66 | 1.21±0.30 |

Note:Dropout:**Combination group(missing visit:1 case、falling off due to adverse events:1 case、exceeded visit time:3 cases、violation of blood lipid inclusion criteria:4 cases)**

**Single statin group(missing visit:2 cases、falling off due to adverse events:2 cases、exceeded visit time:1 case、violation of blood lipid inclusion criteria:2 cases)**
